# Calpain2 Upregulation Regulates EMT-Mediated Pancreatic Cancer Metastasis *via* the Wnt/β-Catenin Signaling Pathway

**DOI:** 10.3389/fmed.2022.783592

**Published:** 2022-05-30

**Authors:** Xiulan Peng, Rui Yang, Jia Song, Xia Wang, Weiguo Dong

**Affiliations:** ^1^Department of Oncology, The Second Affiliated Hospital of Jianghan University, Wuhan, China; ^2^Department of Vascular Surgery, The Second Affiliated Hospital of Jianghan University, Wuhan, China; ^3^Departments of Institute, The Third Affiliated Teaching Hospital of Xinjiang Medical University, Affiliated Cancer Hospital, Ürümqi, China; ^4^Department of Pharmacy, The Second Affiliated Hospital of Jianghan University, Wuhan, China; ^5^Department of Gastroenterology, Renmin Hospital of Wuhan University, Wuhan, China

**Keywords:** calpains2, epithelial-mesenchymal transition, pancreatic cancer, Wnt/β-catenin pathway, therapeutic target

## Abstract

Calpains2 (CAPN2) is a calcium-dependent, non-lysosomal cysteine protease that plays critical roles in normal cellular functions and pathological processes, including tumorigenesis, cancer progression, and metastasis. However, the role and underlying regulatory mechanisms of CAPN2 in pancreatic cancer (PC) are still unknown. We found that CAPN2 is highly expressed in PC tissues and associated with poor PC prognosis by using The Cancer Genome Atlas (TCGA) datasets, Gene Expression Omnibus (GEO) datasets, and PC tissue arrays. CAPN2 downregulation significantly inhibited cell proliferation, migration, and invasion and regulated Wnt/β-catenin signaling pathway-mediated epithelial-mesenchymal transition (EMT) in PC cells. Our findings highlight the significance of CAPN2 in tumor regression and, thus, indicate that CAPN2 could be a promising target for PC treatment.

## Introduction

Pancreatic cancer (PC) is one of the leading causes of cancer-related mortality and one of the most lethal malignant neoplasms worldwide, leading to an estimated 432,242 deaths per year worldwide (1). Although previous progress has been made in the management of patients with pancreatic cancer, the 5-year survival is still extremely poor due to cancer metastasis (2, 3). Therefore, it is necessary to elucidate the molecular mechanisms involved in the metastasis of PC to improve clinical outcomes.

The calpain (CAPNS) family is a calcium dependent, non-lysosomal cysteine protease that has been proven to play pivotal roles in a wide range of cellular and molecular functions and physiological processes (4, 5). Calpain 2 (CAPN2) is one of the most widely investigated members of this family (6), and it is expressed throughout the body, including the heart, nervous system, reproductive system, and gastrointestinal tract (7). The functions of CAPN2 are mainly mediated by Ca^2+^ autoproteolysis, phosphorylation, and intracellular distribution (8, 9). CAPN2 requires millimolar-range concentrations of Ca^2+^ and membrane association for their intracellular activity (8). In various pathological states, abnormally elevated concentrations of calcium can activate CAPN2, which is involved in cellular signaling, cytoskeletal remodeling, cell survival, apoptosis, and cancer pathogenesis and progression (10, 11).

Accumulating experimental and clinical studies have shown that activation of CAPN2 plays a critical role in tumorigenesis and progression in various tumors, including ovarian cancer (12), colorectal cancer (13), gastric cancer (14), hepatocellular carcinoma (15), non-small cell lung cancer (16), and prostate cancer (17). In addition, the calpain inhibitor calpeptin can suppress malignant behavior in PC (18). However, the precise role and the potential underlying mechanism of CAPN2 in PC have not yet been investigated.

Epithelial-mesenchymal transition (EMT) is a process in which tumor cells lose their epithelial characteristics and gain mesenchymal features that enable them to migrate and invade distant sites (19). Abundant evidence has indicated that EMT is related to tumor cell progression, migration, metastasis, and therapy and is a critical step in the invasion and metastasis of PC (20–22). EMT is characterized by the upregulated expression of mesenchymal markers and extracellular matrix components (N-cadherin, vimentin, collagens, and fibronectin) and reduced expression of epithelial markers (E-cadherin and α- and γ-catenin) (23). There are several signaling pathways that regulate EMT, including Wnt/β-catenin, TGF-β, EGF, Hedgehog, and Notch, among which the Wnt/β-catenin signaling pathway plays crucial roles (24, 25). E-cadherin is repressed by several Wnt/β-catenin signaling pathway members, such as Snail, Twist, and Slug. Activation of the Wnt signaling pathway can inhibit β-catenin phosphorylation, thereby facilitating β-catenin translocation to the nucleus and binding to transcription factors to promote EMT (26).

In this study, we investigated the prognostic significance of CAPN2 expression in pancreatic cancer tissues. Furthermore, the relationship of CAPN2 messenger RNA (mRNA) and protein in pancreatic cancer cells and pancreatic HTERT-HPNE cells was studied. Finally, we analyzed the relationship between CAPN2 and EMT and the Wnt/β-catenin pathway using CAPN2 knockdown pancreatic cell lines.

## Materials and Methods

### Gene Expression Data From Public Databases

The mRNA level of CAPN2 in pancreatic cancer and matched adjacent normal pancreatic tissues was analyzed using The Cancer Genome Atlas (TCGA) and GEO (GSE15471, GSE16515, GSE28735, GSE71729, and GSE71989) databases (https://www.ncbi.nlm.nih.gov/geo/). The prognostic value of CAPN2 mRNA levels in patients with pancreatic cancer was investigated using the Kaplan–Meier Plotter (http://kmplot.com/analysis/) database.

### Human Tissue Arrays

Human tissue arrays (HPanA120Su02) that contained 64 PC tissues and 54 matched adjacent normal pancreatic tissues were obtained from Shanghai Outdo Biotech Co. Ltd. These arrays included sex, age, survival time, pathology, tumor-node-metastasis (TNM) stage, and tumor grade data. This study was approved by the Institutional Review Board (IRB) of Renmin Hospital of Wuhan University.

### Immunohistochemistry

Tissue sections of tumors in the tissue arrays were deparaffinized, washed with 3% H_2_O_2_, and then subjected to antigen retrieval with citric acid (pH, 6). After incubation with the anti-CAPN2 protein antibody (1:200 dilution, HPA024470, Sigma Aldrich), the tissue sections were developed using 3,3'-diaminobenzidine (DAB) and counterstained with hematoxylin. In the negative control group, phosphate-buffered saline (PBS) was used as the primary antibody. Immunohistochemistry (IHC) staining was assessed separately by two experienced pathologists using a semiquantitative scoring system according to the intensity of staining and the percentage of positive cells. The staining intensity was scored as follows: 0 (negative), 1 (weak), 2 (moderate), and 3 (strong). The percentage of positive-stained cells was 0 (0%), 1 (<25%), 2 (25–50%), 3 (50–75%), and 4 (>75%). The final staining scores were calculated by multiplying the staining intensity and the percentage of positive-stained cells. The median score was used to categorize the low or high CAPN2 protein expression groups.

### Cell Line Cultures

Three human pancreatic cancer cell lines, Sw1990, Capan-2, and Panc-1, and the normal human pancreatic duct cell line HTERT-HPNE were obtained from the China Centre for Type Culture Collection (Wuhan, China). The pancreatic cancer lines were cultured in DMEM/F-12 (1:1) (HyClone, Logan, UT, USA) with 10% fetal bovine serum (FBS) (Gibco, Thermo Fisher Scientific, Waltham, MA, USA) at 37°C in an incubator containing 5% CO_2_. The HTERT-HPNE cell line was maintained in icell-h102-001b with 10% fetal calf serum (Gibco-BRL, Grand Island, NY, USA) and cultured at 37°C with 5% CO_2_.

### CAPN2-Specific Small Interfering RNA (siRNA) Constructs and Chemicals

We purchased CAPN2 silencing siRNAs from GenePharma (Suzhou, China). The CAPN2-specific siRNAs were as follows: siCAPN2#1, 5′- GAGGCCATCACGTTTCAGA−3′; siCAPN2#2, 5′-TCACCAGCGATACCTACAA-3′; siCAPN2#3, 5′- TGACCAGACGGCATGAAGA-3′; and siNC, GCATAGTTCACTTTCACCTGGGTCT. We then transfected Panc-1 cells with control and CAPN2-silencing siRNAs using Lipofectamine 3,000 (Invitrogen, USA) according to the manufacturer's instructions. CHIR-99021 was purchased from MedChemExpress (HY-10182).

### Western Blot

We extracted total proteins from pancreatic cell lines using radioimmunoprecipitation assay (RIPA) lysis buffer (AS1004, ASPEN, Wuhan). Proteins were extracted from the four pancreatic cell lines Sw1990, Capan-2, Panc-1, and HTERT-HPNE. Total protein fractions were separated on 10% sodium dodecyl sulfate–polyacrylamide (SDS–PAGE) gels. Then, the proteins were subjected to electrophoresis followed by primary and secondary antibody treatment. Finally, the proteins were transferred to polyvinylidene difluoride (PVDF) membranes, and the protein bands were visualized under enhanced chemiluminescence. The levels of CAPN2 protein were normalized to GAPDH on the same PVDF membranes (Merck Millipore, Burlington, MA, USA).

### Immunofluorescence Staining

The cells were fixed with 4% paraformaldehyde (AS1018, Aspen, Wuhan) for 20 min. Then, the fixed cells were blocked with 10% bovine serum albumin (AS1018, Aspen, Wuhan) for 30 min, followed by incubation at 4°C overnight with the following primary antibodies: anti-E-cadherin (20874-1-AP, San Ying, Wuhan), anti-N-cadherin (Abcam, Ab18203), and anti-β-catenin (20874-1-AP, San Ying, Wuhan). After incubating the cells with CY3- or FITC-conjugated goat anti–mouse or goat anti–rabbit IgG (AS-1111, Aspen, Wuhan) secondary antibody at 37°C for 40 min, they were captured using fluorescence microscopy.

### RT–qPCR

Total RNA was extracted from pancreatic cell lines in the downregulation and control groups using TRIzol reagent as previously described (27). Complementary DNA was created using AMV reverse transcriptase (GIBCO, Galthersburg, MD, USA). The 2^−ΔΔCt^ method was used to quantify the relative expression of CAPN2 normalized to the endogenous GAPDH control, and each sample was analyzed in triplicates. The primers for CAPN-2 were 5′-CCAAAATGGATGGGAACTGG-3′ (F) and 5′-ATAGATGCCAAAGCCGATGG-3′ (R) and 5′-CATCATCCCTGCCTCTACTGG′ (F) and 5′-GTGGGTGTCGCTGTTGAAGTC-3′ (R) for GAPDH.

### CCK8 Assay

The CCK-8 assay was used to estimate the viability of PC cells. We seeded 10^5^ cells per well of the experimental and control pancreatic cells into 96-well-plates for 24, 48, and 72 h. Then, we added 10 μL of CCK-8 reagent (C0038, Biyuntian Biotechnology Company) to each well and performed incubation at 37°C for another 2 h. The absorbance at a wavelength of 450 nm was measured using a plate reader.

### Wound Scratch Assay

The PC cells were seeded into 6-well-plates and incubated overnight. A sterile 100-μl pipette tip was used to scratch a wound in a monolayer of pancreatic cells, followed by mild PBS washing. The wound healing or cell migration ability of the pancreatic cells was recorded by capturing individual sections at 0, 6, 12, and 24 h time points.

### Transwell Migration and Invasion Assay

The migration and invasion of PC cells were determined using the Transwell assay. For the invasion assay, the polycarbonate membrane was coated in Transwell chambers with Matrigel (Corning, USA). A total of 2 x 10^4^ cells in serum-free medium were transferred into the top chamber, and medium with serum was added to the bottom chamber. After incubation at 37°C for 24 h, the non-invading cells on the top side of the membrane were removed by scrubbing, and the migrating or invading cells were fixed with 4% paraformaldehyde (PFA), and then stained with.1% crystal violet. Light microscopy was utilized to analyze and count the migrated or invaded cells.

### Flow Cytometry Analysis of Cellular Apoptosis

The apoptosis of each group of PC cells was estimated with an apoptosis detection kit (AO2001-02P-G, Sanjian, Tianjin). We centrifuged 5 × 10^5^ cells, washed them three times with PBS and resuspended them in binding buffer. Thereafter, 5 μl of Annexin V-FITC and propidium iodide (PI) were added to the cells, followed by incubation at 37°C in the dark. Finally, the cells were analyzed with flow cytometry (BD Biosciences). The cellular apoptosis data were analyzed by FlowJo V10 software (Tree Star, San Francisco, CA, United States).

### Statistical Analysis

The SPSS version 20.0 (IBM Inc., Chicago, IL, USA) and GraphPad Prism 5 (GraphPad Software, CA, USA) software was used to perform the data analyses. Student's *t*-tests were applied to assess the differences between the two groups. Pearson's χ2 tests were utilized to evaluate the correlation between CAPN2 expression and clinicopathological data. Kaplan–Meier plotter was used to evaluate the prognostic significance of CAPN2. The independent risk factors for survival were analyzed using a Cox regression model. A *P*-value of < 0.05 was considered statistically significant.

## Results

### CAPN2 mRNA Levels Are Significantly Upregulated in PC Tissues

**Figure 1:**
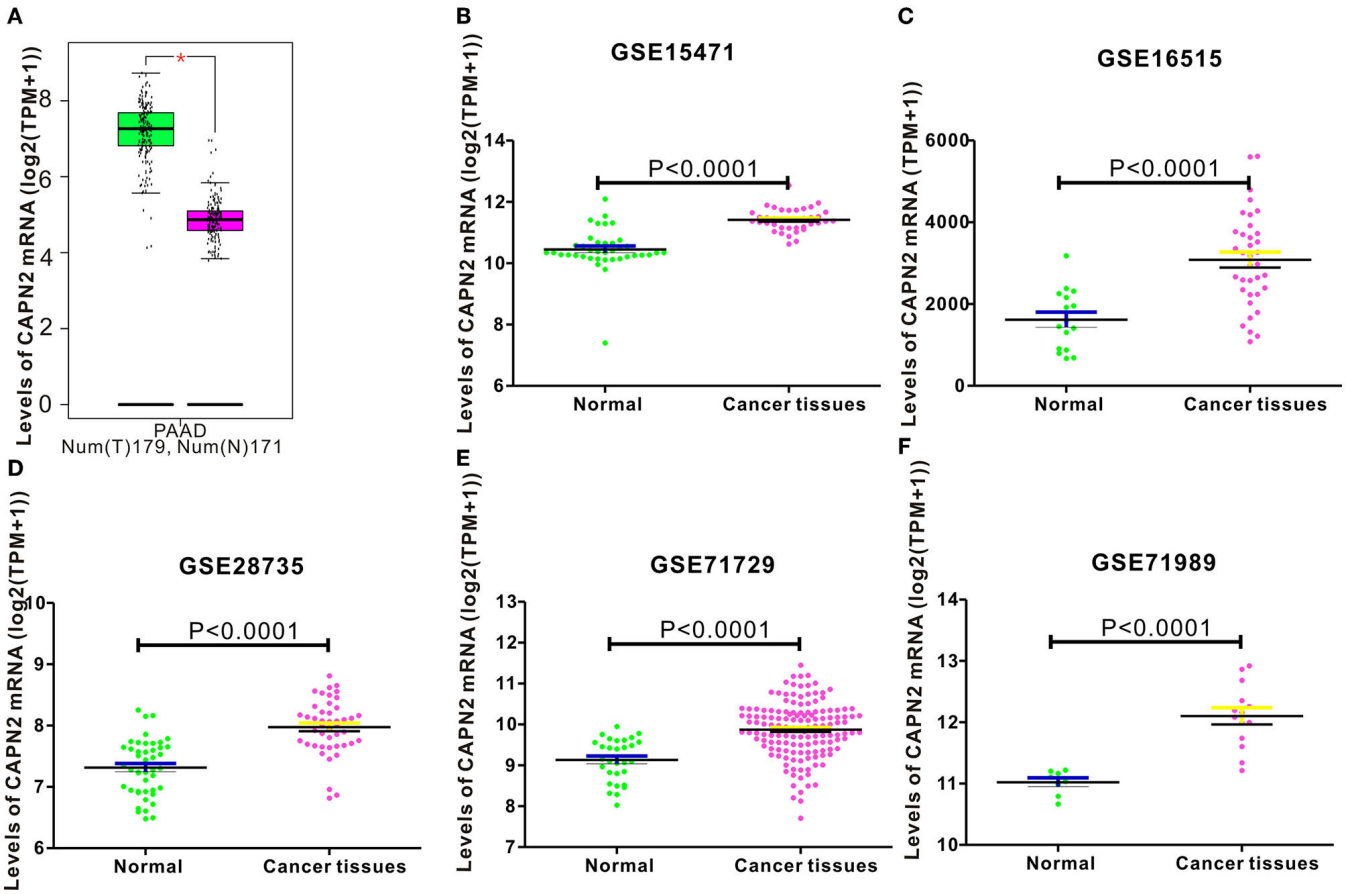
Analysis of CAPN2 mRNA expression in normal and pancreatic cancer (PC) tissues from public databases. CAPN2 mRNA levels were significantly lower (*P* < 0.0001) in normal pancreatic tissue than in pancreatic cancer tissue samples in the **(A)** STAD dataset (Normal = 171; Tumor = 179) from the TCGA database; **(B)** GSE15471 (Normal = 39; Tumor = 39); **(C)** GSE16515 (Normal = 16; Tumor =36); **(D)** GSE28735 (Normal = 45; Tumor = 45); **(E)** GSE71729 (Normal = 30; Tumor = 145); **(F)** GSE71989 (Normal = 8; Tumor = 14) datasets from the GEO database.

**Figure 2:**
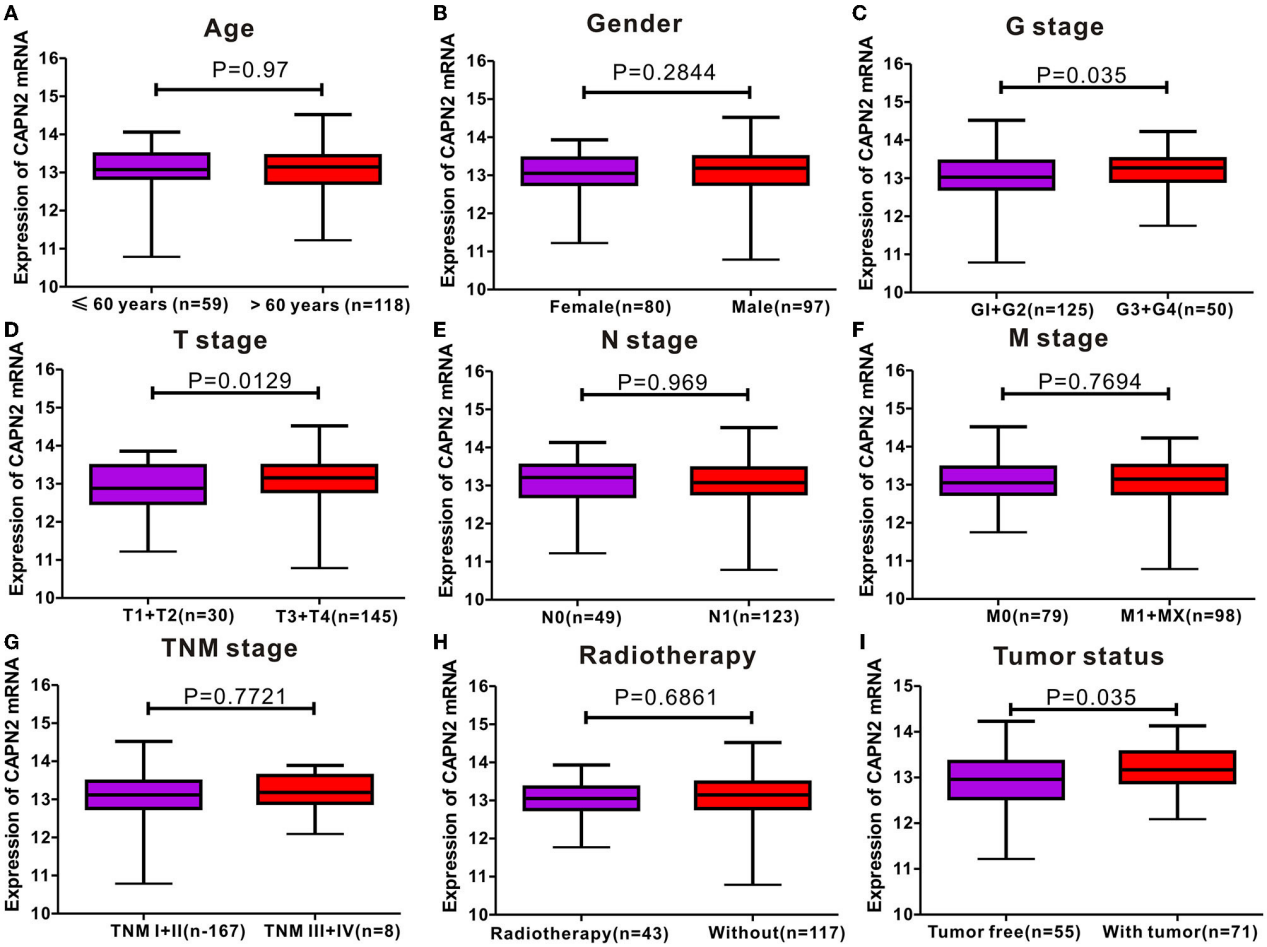
Correlation analyses between CAPN2 mRNA levels and different clinicopathological characteristics of patients with PC. The association between CAPN2 levels and clinicopathological characteristics of patients with PC, including **(A)** age (*P* = 0.97); **(B)** sex (*P* = 0.2844); **(C)** tumor grade **(G)** stage (*P* = 0.035); **(D)** tumor (T) stage (*P* = 0.0129); **(E)** node N stage (*P* = 0.969); **(F)** metastasis M stage (*P* = 0.7694); **(G)** tumor node metastasis (TNM) stage (*P* = 0.7721); **(H)** radiotherapy (*P* = 0.6861); **(I)** tumor status (*P* = 0.035).

**Figure 3:**
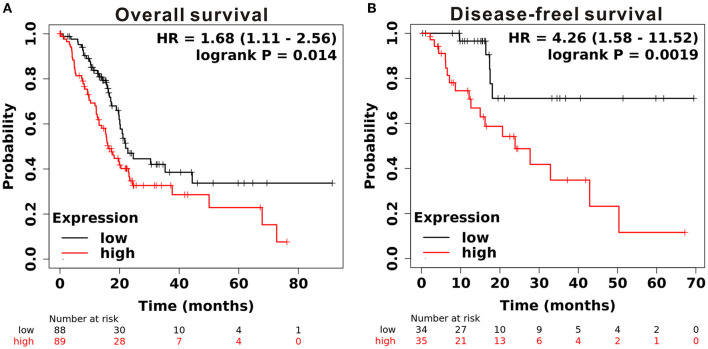
Prognostic significance of CAPN2 mRNA levels in patients with PC. **(A)** Patients with high CAPN2 mRNA levels showed poorer OS than patients with low CAPN2 levels in the Kaplan–Meier Plotter database (*N* = 177, HR = 1.68, *P* = 0.014). **(B)** Patients with high CAPN2 mRNA levels showed poorer RFS than patients with low CAPN2 mRNA levels in the Kaplan–Meier Plotter database (*N* = 69, HR = 4.26, *P* = 0.0019).

To determine the expression level of CAPN2 in PC, we first used the open Gene Expression Omnibus (GEO) database, and The Cancer Genome Atlas (TCGA) dataset were used to analyze the mRNA expression of CAPN2 in PC. Analysis of data obtained from these datasets showed significantly higher CAPN2 mRNA expression in PC tissues than in adjacent normal tissues (Figure 1). Furthermore, we explored the correlation between CAPN2 mRNA levels and clinicopathological characteristics using the TCGA-PAAD dataset and revealed that patients with PC with high CAPN2 mRNA levels showed poorer tumor grade, T stage, and tumor status than patients with PC with lower CAPN2 levels (Figure 2). Moreover, we assessed the prognostic value of CAPN2 in PC by analyzing the association of CAPN2 mRNA expression with overall survival (OS) and relapse-free survival (RFS) using TCGA datasets. The results showed that high CAPN2 mRNA levels were associated with poorer OS (HR = 1.68, 95% CI = 1.11–2.56, *P* = 0.014, Figure 3A) and PFS (HR = 4.26, 95% CI = 1.58–11.52, *P* = 0.0019, Figure 3B) than low CAPN2 expression in patients with PC.

### CAPN2 Protein Is Highly Expressed in PC Tissues

**Figure 4:**
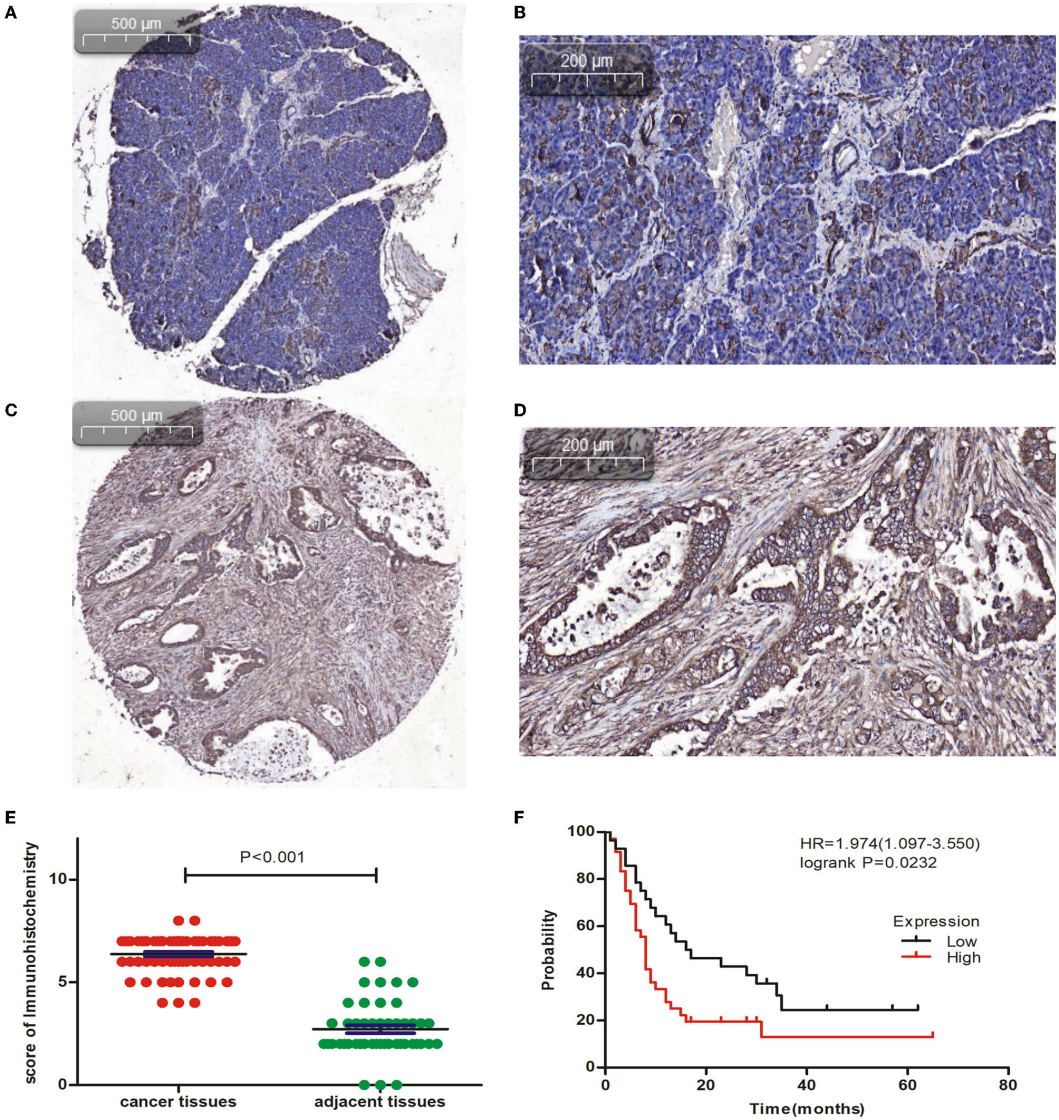
Immunohistochemical analysis of CAPN2 expression in human PC tissues. **(A,B)** Representative IHC staining images of CAPN2 protein in noncancerous pancreatic tissues and **(C,D)** PC tissues at X5 and X20 magnification, respectively. **(E)** Comparison of the CAPN2 expression score shows that CAPN2 protein levels are significantly higher (*P* < 0.001) in PC tissues (*N* = 64) than in noncancerous pancreatic tissues (*N* = 54). **(F)** Kaplan–Meier analysis shows that high expression of CAPN2 is associated with poorer OS than low expression of CAPN2 in patients with PC (HR = 1.974, *P* = 0.0232).

The IHC analysis of 64 PC specimens showed that the cytoplasmic expression of CAPN2 was significantly higher in PC tissues than in non-cancerous pancreatic tissues (Figures 4A–D). As shown in Figure 4E, positive CAPN2 protein staining was significantly higher in the PC tissues than in the adjacent normal pancreatic tissues (*P* < 0.001). Table 1 shows the association between CAPN2 protein levels and the clinicopathological parameters in 64 patients with PC. CAPN2 protein expression was significantly higher in patients with a higher tumor grade (*P* = 0.0482), but showed no association with sex, age, T stage, N stage, M stage, or TNM stage. Furthermore, patients with PC with high CAPN2 protein expression showed poorer OS than patients with PC with low CAPN2 expression, as analyzed by Kaplan–Meier survival analysis (Figure 4F). Univariable Cox analysis showed that high CAPN2 protein expression was a prognostic factor in patients with PC (Table 2). Multivariate Cox analysis demonstrated that tumor grade was an independent prognostic factor (HR = 2.888; 95% CI = 1.338–6.234; *P* = 0.007) in patients with PC after adjusting for the N and TNM stages (Table 2). Taken together, our data demonstrate that high CAPN2 protein expression is associated with poorer survival rates in patients with PC.

**Table 1:** Correlation between CAPN2 expression and clinicopathologic features of 64 patients with pancreatic carcinoma.

**Clinical features**		**CAPN2 expression**		***P*-value**
		**Low (*n* = 29)**	**High (*n* = 35)**	
Age	≥60	13	28	0.063
	<60	14	9	
Gender	Male	8	18	0.339
	Female	21	17	
Histologic grade	G1/G2	24	21	0.048
	G3/G4	5	14	
AJCC stage	Stage I/II	25	28	0.892
	Stage III/IV/X	4	7	
T stage	T1/T2	7	7	0.967
	T3/T4/Tx	21	25	
N stage	N0	21	18	0.252
	N1/NX	8	17	
M stage	M0	27	32	0.872
	M1/MX	2	3	

**Table 2:** Univariable and multivariable Cox analysis of CAPN2 expression for OS.

**Variables**	**Univariable**			**Multivariable**		
	**HR**	**95%CI**	***P*-value**	**HR**	**95%CI**	***P*-value**
**Age (years)**						
≥60	1.188	0.662–2.133	0.563			
<60	Ref.	-	-			
**Gender**						
Male	1.477	0.811–2.688	0.202			
Female	Ref.	-	-			
**Histologic grade**						
G1+G2	Ref.	-	-			
G3+G4	3.758	2.004–7.048	<0.001	2.888	1.338–6.234	0.007
**T stage**						
T1+T2	Ref.	-	-			
T3+T4	1.649	0.792–3.434	0.182			
**N stage**						
N0+NX	Ref.	-	-			
N1	1.594	0.864–2.941	0.136			
**M stage**						
M0+MX	Ref.	-	-	-	-	-
M1	3.314	1.141–9.632	0.028	1.703	0.452–6.413	0.431
**AJCC stage**						
I+II	Ref.	-	-	-	-	-
III+IV	3.447	1.178–10.032	0.024			
**CAPN2 expression**						
Low	Ref.	-	-	-	-	-
High	0.445	0.245–0.809	0.008	0.583	0.283–1.202	0.144

### CAPN2 mRNA and Protein Are Upregulated in Pancreatic Cancer Cell Lines

**Figure 5:**
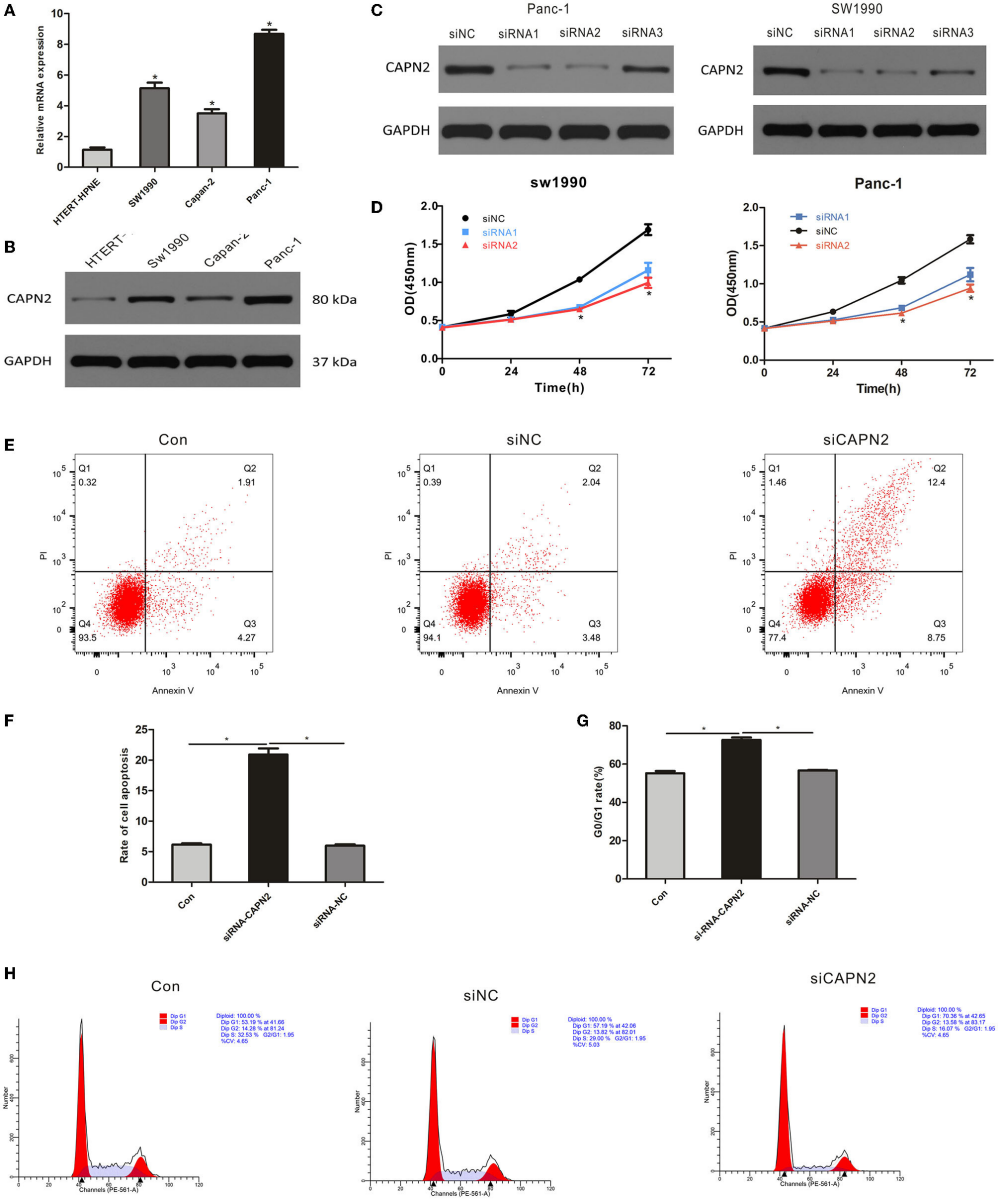
CAPN2 expression regulates the proliferation and survival of PC cell lines **(A,B)** qRTPCR and Western blot analysis of CAPN2 expression in the three PC cell lines Sw1990, Capan-2, and Panc-1 and the normal human pancreatic duct cell line HTERT-HPNE. **(C)** Knockdown effects of CAPN2 were confirmed by Western blotting analyses. **(D)** Proliferation of CAPN2-depleted cells and control cells at 24 h, 48 h, and 72 h was determined by the CCK-8 assay. **(E,F)** The effect of CAPN2 on the apoptotic rate in PC cells at 48 h was determined by flow cytometry analysis. Representative images and the quantitative analysis of the apoptotic rate in siCAPN2 #2-transfected Panc-1 cells and control cells. **(G,H)** Cell cycle analysis results from CAPN2 knockdown and control Panc-1 cells (48 h). Representative images and quantitative analysis of G0/G1-phase arrest are shown here. *P < 0.05.

The protein and mRNA levels of CAPN2 were evaluated in three pancreatic cancer cell lines, Sw1990, Capan-2, and Panc-1, and a normal human pancreatic duct cell line by Western blotting and RT–qPCR. The results showed that the expressions of CAPN2 protein and mRNA were significantly higher in PC cell lines than in the HTERT-HPNE cell line (Figures 5A,B). Among the PC cell lines, Panc-1 cells showed the highest CAPN2 expression, and Capan-2 cells showed the lowest CAPN2 expression. We selected Panc-1 and SW1990 cells for subsequent experiments.

### CAPN2 Regulates the Proliferation and Survival of PC Cells

To determine the potential biological function of CAPN2 in PC cells, we transfected siRNAs to knock down CAPN2 in Panc-1 and SW1990 cells. The transfection efficiency was verified in Panc-1 and SW1990 cells using Western blot analysis, which showed that Panc-1 and SW1990 cells transfected with siCAPN2 #1 and #2 showed significant CAPN2 downregulation compared to siCAPN2 #3 and siNC (Figure 5C). Therefore, we selected Panc-1 and SW1990 cells transfected with siCAPN2#1 and #2 for further experiments. The data from the Cell Counting Kit-8 (CCK-8) assays showed that CAPN2 knockdown significantly inhibited the proliferation of PC cells (Figure 5D). Furthermore, we analyzed the cell cycle distribution and apoptosis using flow cytometry, and discovered that G0/G1-phase cells accumulated and that the apoptosis rate significantly increased following CAPN2 knockdown in Panc-1 cells (Figures 5E–H). These data suggest that CAPN2 depletion plays a suppressive role in the proliferation and survival of PC cells.

### CAPN2 Regulates PC Cell Migration and Invasion

**Figure 6:**
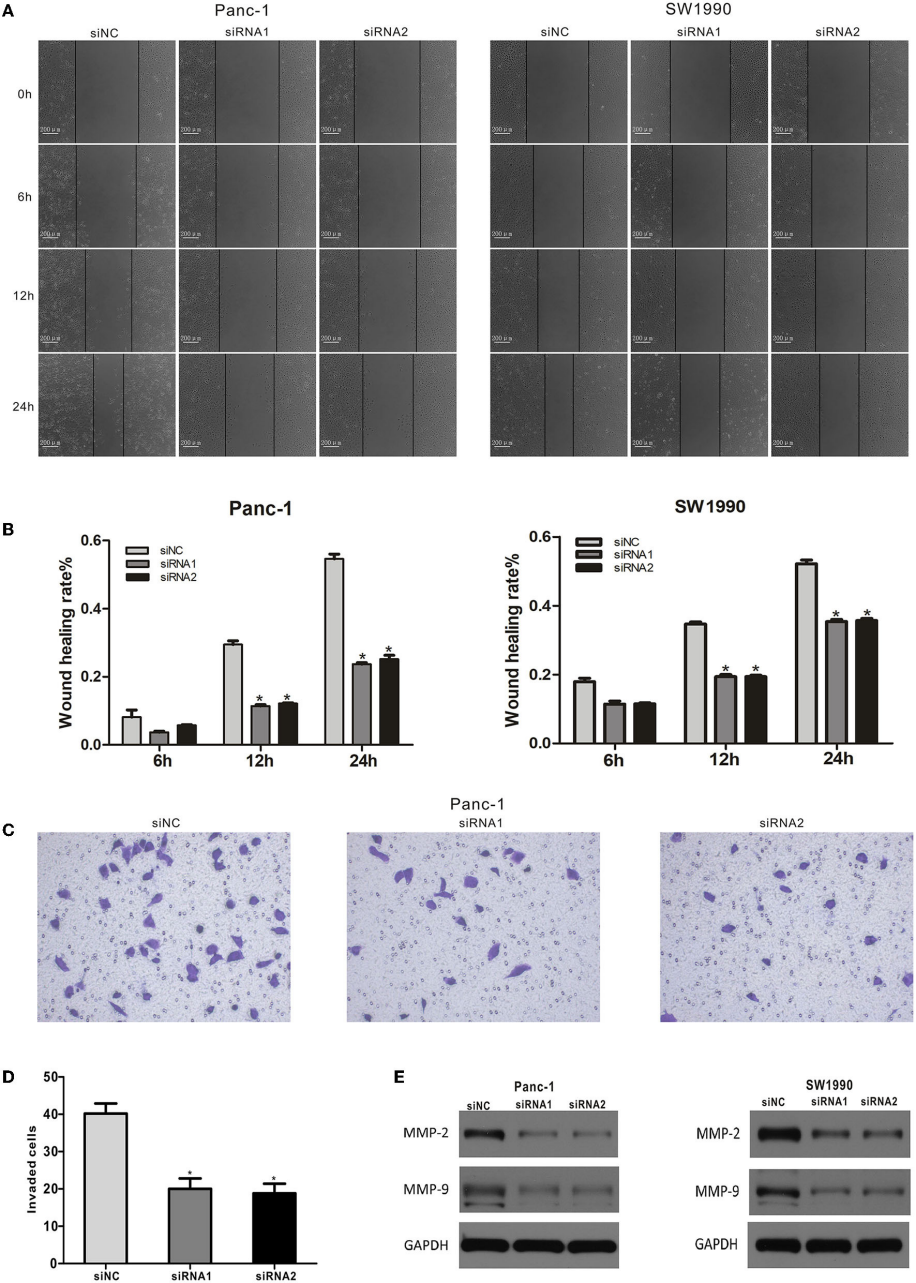
CAPN2 expression regulates the migration and invasion of PC cells *in vitro*. **(A,B)** The migratory abilities of siRNA1,2-transfected Panc-1 cells and SW1990 cells were detected by a wound healing assay. Representative images and the quantitative analysis of the distance between wound edges at 6, 12, and 24 h. **(C,D)** Cell migratory abilities were detected by the Transwell migration assay. Quantitative analysis and representative images of the number of migrating cells in siRNA#2-transfected Panc-1 cells and control cells. **(E)** Western blot analysis shows that MMP9 and MMP2 protein levels were significantly reduced in CAPN2-silenced Panc-1 cells and SW1990 cells compared with that in control cells. *P < 0.05.

We used a wound healing assay to assess the effects of CAPN2 silencing on the migration activity of PC cells. The results indicated that decreased expression of CAPN2 reduced the migration of PC cells compared with the corresponding controls (Figures 6A,B). In addition, the Transwell invasion assay revealed significantly decreased invasiveness after CAPN2 silencing in Panc-1 cells compared with the control (Figures 6C,D).

The expression of matrix metalloproteinases (MMPs) is a key regulator of the migration potential of cancer cells (28). Therefore, we performed Western blotting to detect the expression levels of MMP2 and MMP9 in Panc-1 cells. The Western blot data showed that CAPN2 knockdown markedly decreased MMP2 and MMP9 levels (Figure 6E). Taken together, these data indicated that CAPN2 expression regulated PC cell migration and invasion.

### CAPN2 Regulates the Epithelial-Mesenchymal Transition of PC Cells

**Figure 7:**
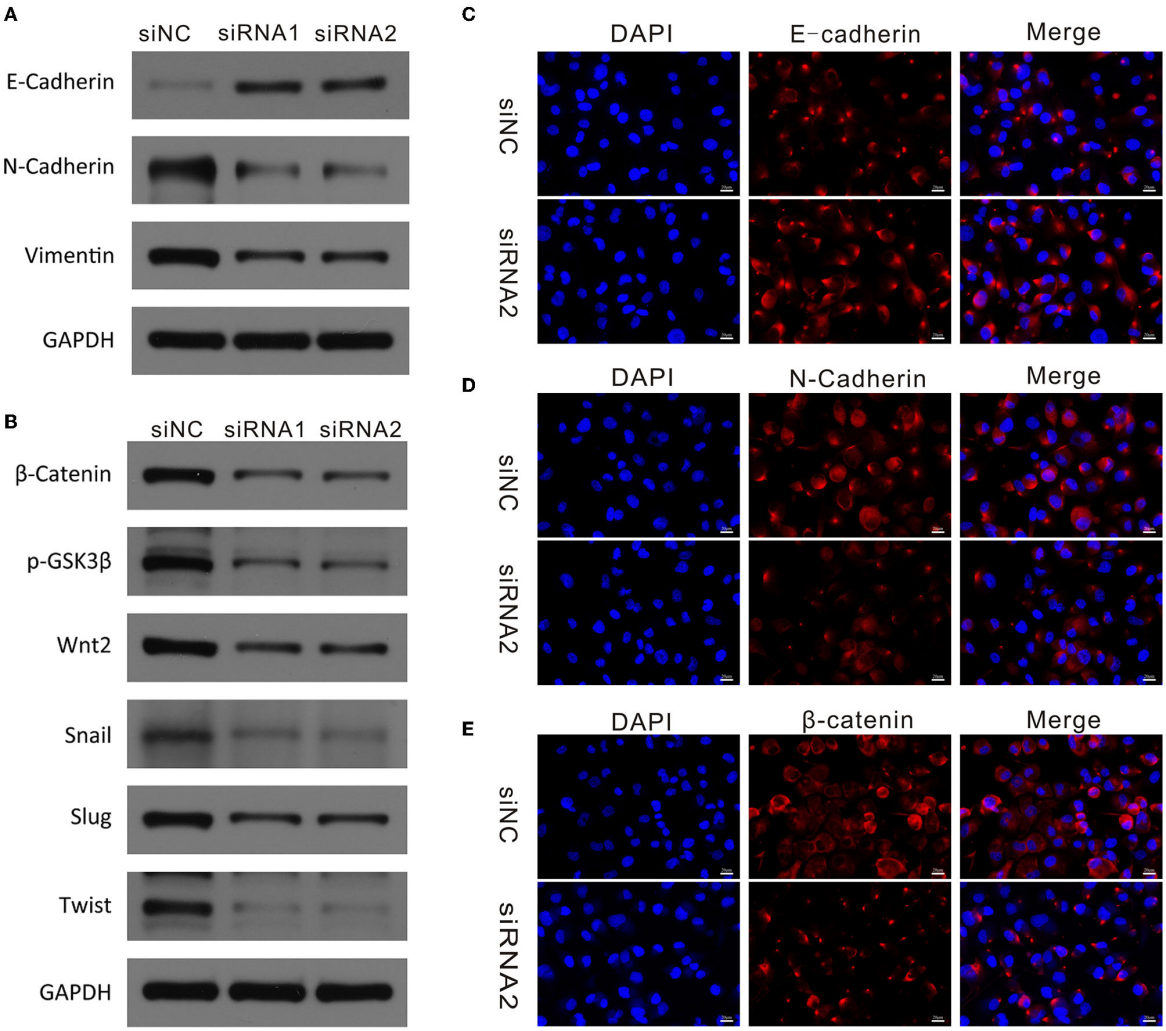
CAPN2 regulates EMT markers and the Wnt/β-catenin pathway in PC. **(A)** Western blot analysis shows reduced N-cadherin and vimentin and increased E-cadherin expression in CAPN2-silenced Panc-1 cells and SW1990 cells compared with that in control cells. **(B)** Western blot analysis shows reduced levels of β-catenin, Wnt2, p-GSK-3β, Snail1, Slug, and TWIST in CAPN2-silenced Panc-1 cells and SW1990 cells compared with that in control cells. **(C–E)** Immunofluorescence staining of E-cadherin, N-cadherin and β-catenin in CAPN2-silenced Panc-1 cells compared with that in control cells.

Next, we explored whether downregulation of CAPN2 was responsible for changes in EMT. It is well-known that E-cadherin (epithelial marker), N-cadherin (mesenchymal marker), and vimentin are specific markers for EMT. Western blot analysis showed that siCAPN2 reversed the significant increase in the expression of E-cadherin and the decrease in the protein expression of N-cadherin and vimentin in PC cells compared with control cells (Figure 7A). We confirmed these results using immunofluorescence assays (Figures 7C,D). These results suggest that EMT was regulated by CAPN2 in PC cells.

### CAPN2 Regulates the Wnt/β-Catenin Pathway in PC Cells

Previous studies have demonstrated that the Wnt/β-catenin signaling pathway plays a vital role in regulating EMT and cell metastasis in cancer (29, 30). Therefore, we investigated whether CAPN2 regulates the classical Wnt/β-catenin pathway. We examined the effect of siCAPN2 on key proteins involved in the Wnt/β-catenin signaling pathway in PC cells. Western blot analysis revealed that β-catenin, Wnt2, Snail1, Slug, and TWIST protein levels were significantly reduced in CAPN2-silenced Panc-1 cells compared with the corresponding controls (Figure 7B). However, no change in the protein expression level of GSK3β was observed with siCAPN2. As previously reported, the degradation of β-catenin is mostly controlled by GSK-3β phosphorylation, which can inhibit GSK-3β activity (31). Then, we examined the protein level of p-GSK-3β and found that CAPN2 downregulation resulted in a decreased level of GSK-3β Ser-9 phosphorylation (Figure 7B). We also tested the effect of CAPN2 knockdown on the expression of β-catenin by using an immunofluorescence assay and found that CAPN2 depletion impaired the accumulation of β-catenin (Figure 7E). Overall, these results suggested that CAPN2 inhibited the Wnt/β-catenin pathway in PC cells.

### CAPN2 Induces EMT and Metastasis Through Wnt/β-Catenin Signaling

**Figure 8:**
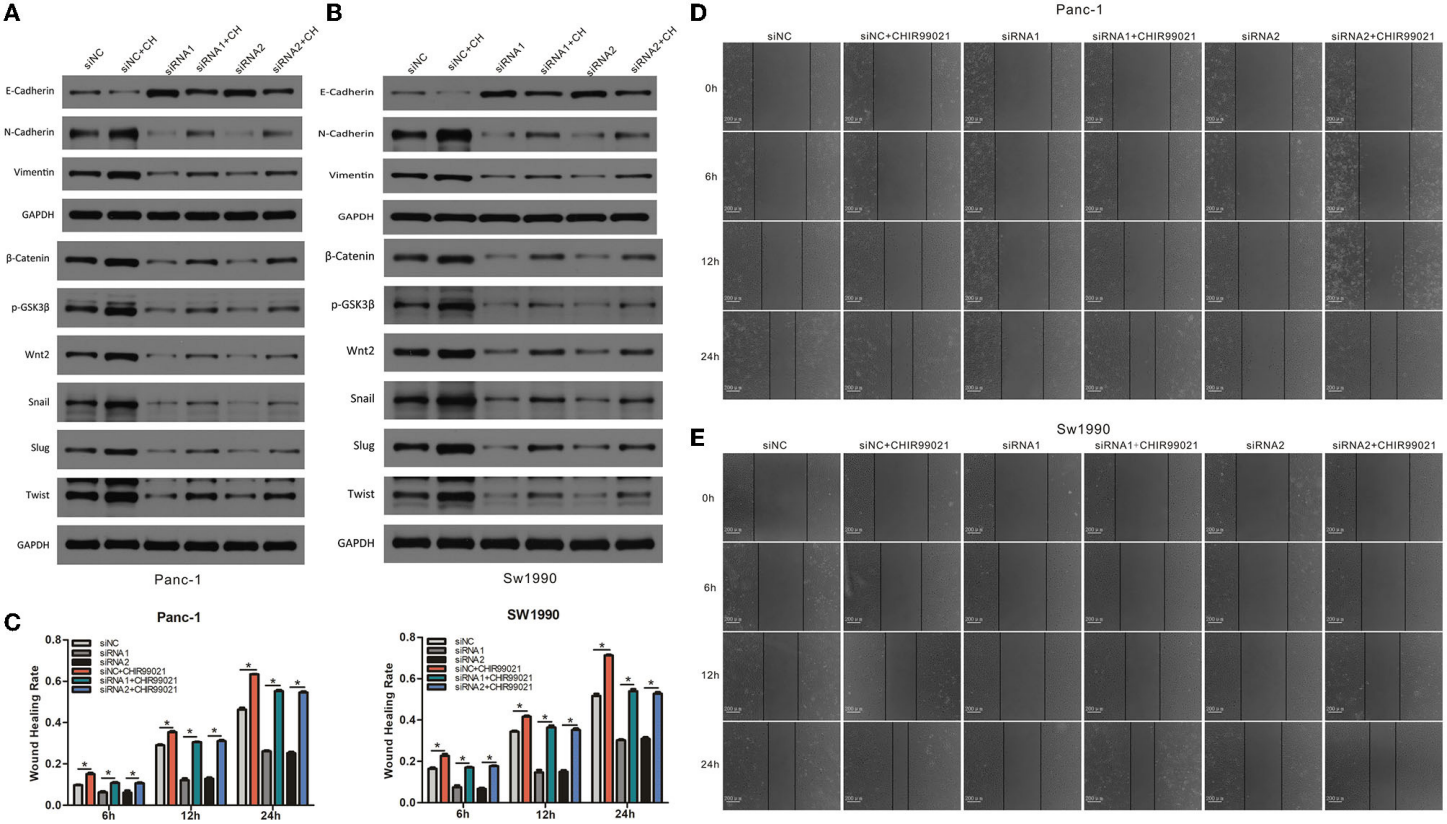
CAPN2 induces EMT and metastasis through Wnt/β-catenin signaling. **(A,B)** Western blot analysis shows increased levels of Wnt2, Snail1, Slug, TWIST, vimentin, and N-cadherin and reduced E-cadherin expression in CAPN2-silenced PC cells compared with that in control cells treated with CHIR-99021 (6 μM, 24 h). **(C–E)** Wound healing assays showed increased cell invasion capacities in CAPN2-silenced PC cells compared with that of control cells treated with CHIR-99021 (6 μM, 24 h). *P < 0.05.

To explore whether the Wnt/β-catenin signaling pathway functions in the EMT, migration and invasion of PC cells, we first examined the effect of Wnt/β-catenin activation. CHIR-99021 is a specific Wnt/β-catenin signaling pathway activator (18). The results showed that CHIR-99021 treatment caused an increase in the β-catenin level in PC cells (Figures 8A,B). Furthermore, activation of Wnt/β-catenin signaling by CHIR-99021 increased the levels of Wnt2, Snail1, Slug, TWIST, vimentin, and N-cadherin (Figures 8A,B). Then, to investigate the function of Wnt/β-catenin signaling in CAPN2-mediated EMT and cell invasion, we performed rescue experiments by activating Wnt/β-catenin signaling in cells with CAPN2 depletion. In CAPN2-depleted cells, CHIR-99021 rescued the impairment of EMT progression and Wnt/β-catenin signaling (Figures 8D,E). Consistent with these findings, in the presence of CHIR-99021, the invasive ability of CAPN2-depleted cells was elevated in PANC-1 and SW1990 cells by the wound healing assay (Figures 8C–E). Taken together, these results indicated that the Wnt/β-catenin signaling pathway may act as a downstream mediator of CAPN2 to control carcinogenesis in human PC.

## Discussion

In this study, we conducted a comprehensive exploration of the role of CAPN2 in the tumorigenesis and progression of PC. We found that the expression of CAPN2 in PC tissues was significantly higher than that in normal adjacent pancreatic tissues. CAPN2 knockdown regulates EMT and metastasis in PC via the Wnt/β-catenin signaling pathway.

Calpain 2 (CAPN2), also known as m-calpain, is composed of an 80-kDa catalytic subunit and a 28-kDa regulatory subunit, which can help maintain its biological activity (9). Studies have reported aberrant expression of CAPN2 in various tumors, and it has a pivotal role in tumorigenesis, disease progression, and clinical outcomes. Miao et al. revealed that the expression of CAPN2 was associated with tumor stage and histological grade (32). The CAPN2 expression level correlates with poor clinical outcome (12). Our observation in PC is in accordance with these reports. We analyzed the expression of CAPN2 mRNA in PC and normal pancreatic tissues from two public databases (TCGA and GEO). The results showed that CAPN2 mRNA levels were significantly higher in PC tissues than in normal pancreatic tissues. Patients with PC with high CAPN2 mRNA levels have a poorer prognosis than those with low CAPN2 mRNA levels in TCGA datasets. Moreover, IHC showed that high CAPN2 protein expression correlated with poorer prognosis in patients with PC. Our observation in PC is in accordance with a study in ovarian cancer patients (12). These data suggest that CAPN2 is a potential prognostic indicator and therapeutic target in patients with PC.

Several studies have shown that downregulation of CAPN2 expression inhibits tumor cell growth and metastasis. For instance, depletion of CAPN2 inhibits cell metastasis and proliferation in renal cell carcinoma (20). Another study demonstrated impaired invasion and metastasis of CAPN2 downregulation in non-small cell lung cancer (NSCLC) (33). SiRNA-mediated downregulation of CAPN2 expression significantly suppressed adhesion, migration, and invasion in HCC cells (15). Although Yoshida et al. demonstrated that calpeptin can reduce the expression of CAPN2 and suppress the tumor progression of PC in a mouse xenograft model (18), to explore the possible functional significance of CAPN2 in PC cells, we knocked down CAPN2 *via* siRNA in Panc-1 and SW1990 cells. We observed that downregulation of CAPN2 not only suppressed cell proliferation, but also inhibited tumor invasion and metastasis in PC cells. The results from PC tissues show some disagreement with the *in vitro* results in the M or N stage. This contradiction is probably due to the small number of cases and the fact that the gene expression level and the protein level are not the same.

Migration and invasion of tumor cells enabled by EMT are necessary conditions for cancer metastasis. Depletion of CAPN2 could inhibit the expression of N-cadherin, vimentin and MMP9, and overexpression of CAPN2 could enhance N-cadherin, vimentin and MMP9 protein levels in renal cell carcinoma (32). Downregulation of CAPN2 expression significantly attenuated MMP-2 and MMP-9 secretion in HCC (15). In addition, inhibition of CAPN2 prevented both focal adhesion disassembly and cell migration (34). These findings suggested that EMT may be a critical mechanism for CAPN2-mediated cancer cell invasion and metastasis. Emerging studies have indicated that the EMT process is a critical step for PC metastasis and progression (20–22). Furthermore, MMPs belong to the family of zinc-dependent endopeptidases, which can reduce the extracellular matrix (ECM) and, thus, have marked effects on tumor progression, invasion, and metastasis (35, 36). Our results showed that knockdown of CAPN2 decreased the expression of N-cadherin, vimentin and MMP9, while augmenting the expression of the epithelial marker E-cadherin in PC. The results provide evidence that CAPN2 disruption prevents PC cell migration and invasion, possibly by interfering with the EMT program and ECM degradation.

The Wnt/β-catenin pathway plays a pivotal role in regulating various cellular activities, such as embryonic development, tissue self-renewal, EMT, and cell invasion (28, 37). The canonical Wnt/β-catenin pathway is triggered when Wnt ligand proteins bind to Frizzled and LRP family receptors on the cell surface (38). Activation of the Wnt/β-catenin pathway increases the nuclear translocation of β-catenin through the protein 1 (Axin1) complex. Nuclear β-catenin can form a complex with the lymphoid enhancer-binding factor/T-cell factor family to regulate Wnt target genes (39). Moreover, activation of the Wnt/β-catenin pathway also phosphorylates GSK3β at serine 9 and, then, inactivates the β-catenin destruction complex, thereby increasing transcription of its target gene SNAIL1 (40). Our results showed that CAPN2 knockdown can inhibit canonical Wnt/β-catenin signaling in PC cells, which was confirmed by the decreased levels of β-catenin and target proteins of the Wnt/β-catenin pathway, including p-GSK-3β, Wnt2, Slug, Snail, and TWIST proteins. Furthermore, CHIR-99021 reversed PC cell functions and EMT markers induced by CAPN2 depletion. We demonstrated that CAPN2 positively activates the Wnt/β-catenin pathway to regulate EMT in PC by upregulating β-catenin and multiple downstream genes of the Wnt/β-catenin signaling pathway. Therefore, these results suggest that CAPN2 regulates the Wnt/β-catenin signaling pathway and modulates the progression of PC.

To our knowledge, our current study examined, for the first time, the clinical significance and molecular function of CAPN2 in PC. However, our study has several limitations. First, the study cohort of patients with PC was relatively small. Second, functional experiments were not performed to verify that CAPN2 regulates the β-catenin signaling pathway. These issues need to be taken into consideration in future investigations. Larger populations of patients and further molecular studies are still needed in future investigations.

## Conclusion

This study demonstrates that CAPN2 was significantly increased in PC tissues and cells and that overexpression of CAPN2 was associated with poorer prognosis. Furthermore, CAPN2 regulates EMT and metastasis through the Wnt/β-catenin signaling pathway in PC. These results indicate a new perspective of CAPN2 as a potential prognostic and therapeutic target in patients with PC.

## Data Availability Statement

The original contributions presented in the study are included in the article/Supplementary Material, further inquiries can be directed to the corresponding author/s.

## Ethics Statement

The studies involving human participants were reviewed and approved by the Institutional Review Board (IRB) of Renmin Hospital of Wuhan University. The patients/participants provided their written informed consent to participate in this study.

## Author Contributions

WD and XP designed the work and approved the final version. XP, RY, JS, and XW performed the experiments. XP, RY, and JS collected the data regarding the paper and wrote the manuscript. RY, JS, and XW analyzed the data. All authors contributed to the article and approved the submitted version.

## Funding

This study was supported by Wuhan Municipal Health Commission (No. WX21D42), Health and Family Planning Foundation of Wuhan (No. WX16A08), and Science and Technology Innovation Talent Program of Urumqi.

## Conflict of Interest

The authors declare that the research was conducted in the absence of any commercial or financial relationships that could be construed as a potential conflict of interest.

## Publisher's Note

All claims expressed in this article are solely those of the authors and do not necessarily represent those of their affiliated organizations, or those of the publisher, the editors and the reviewers. Any product that may be evaluated in this article, or claim that may be made by its manufacturer, is not guaranteed or endorsed by the publisher.
