# Supplementary figures and images for: Calpain2 Upregulation Regulates EMT-Mediated Pancreatic Cancer Metastasis via the Wnt/β-Catenin Signaling Pathway
Source: Front Med (Lausanne). 2022 May 30;9:783592. doi: 10.3389/fmed.2022.783592 (PMC9189366; doi:10.3389/fmed.2022.783592)

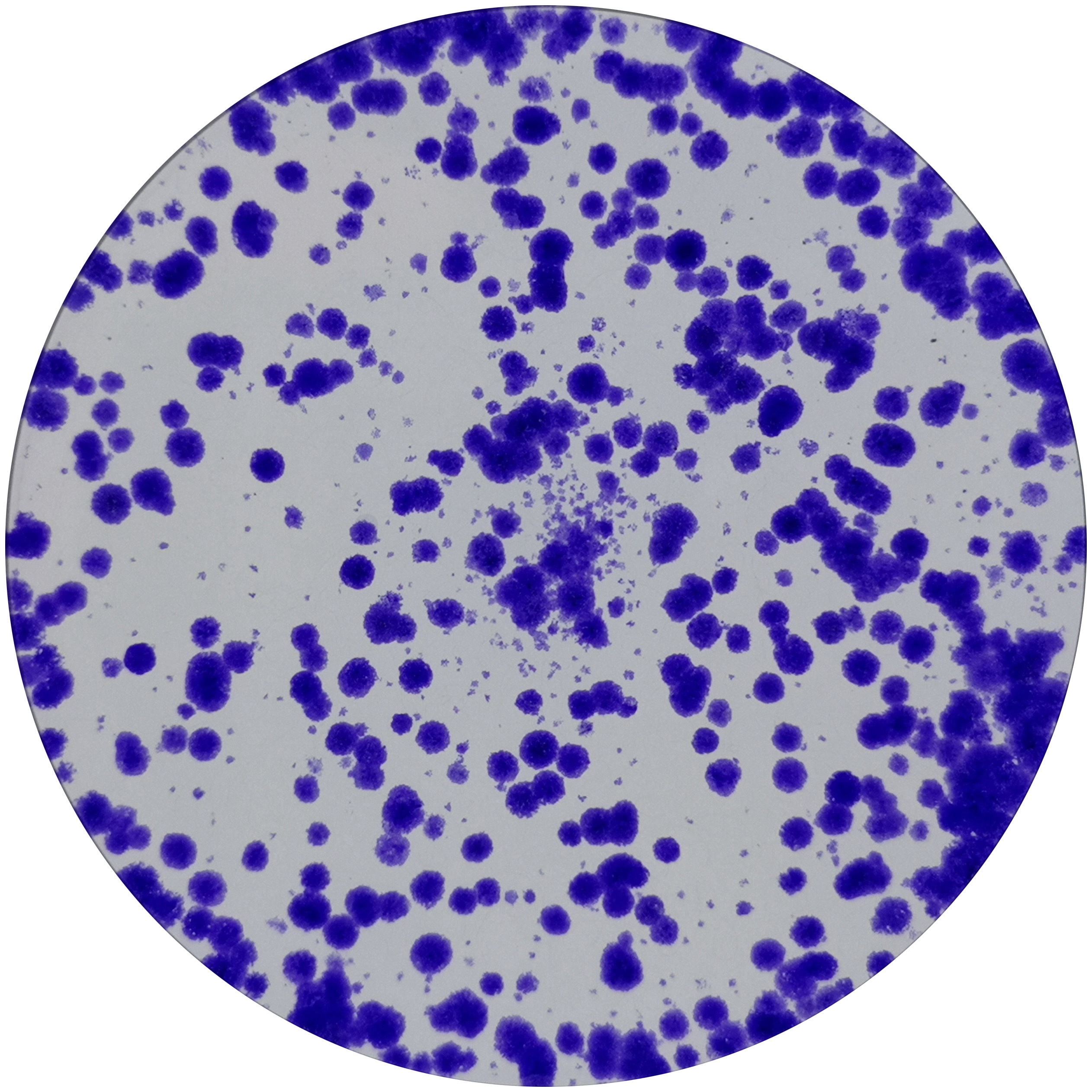

Supplement: Supplementary file 1 [file Data_Sheet_1.ZIP › Raw data1/Colony formation assay/A2-3.jpg]

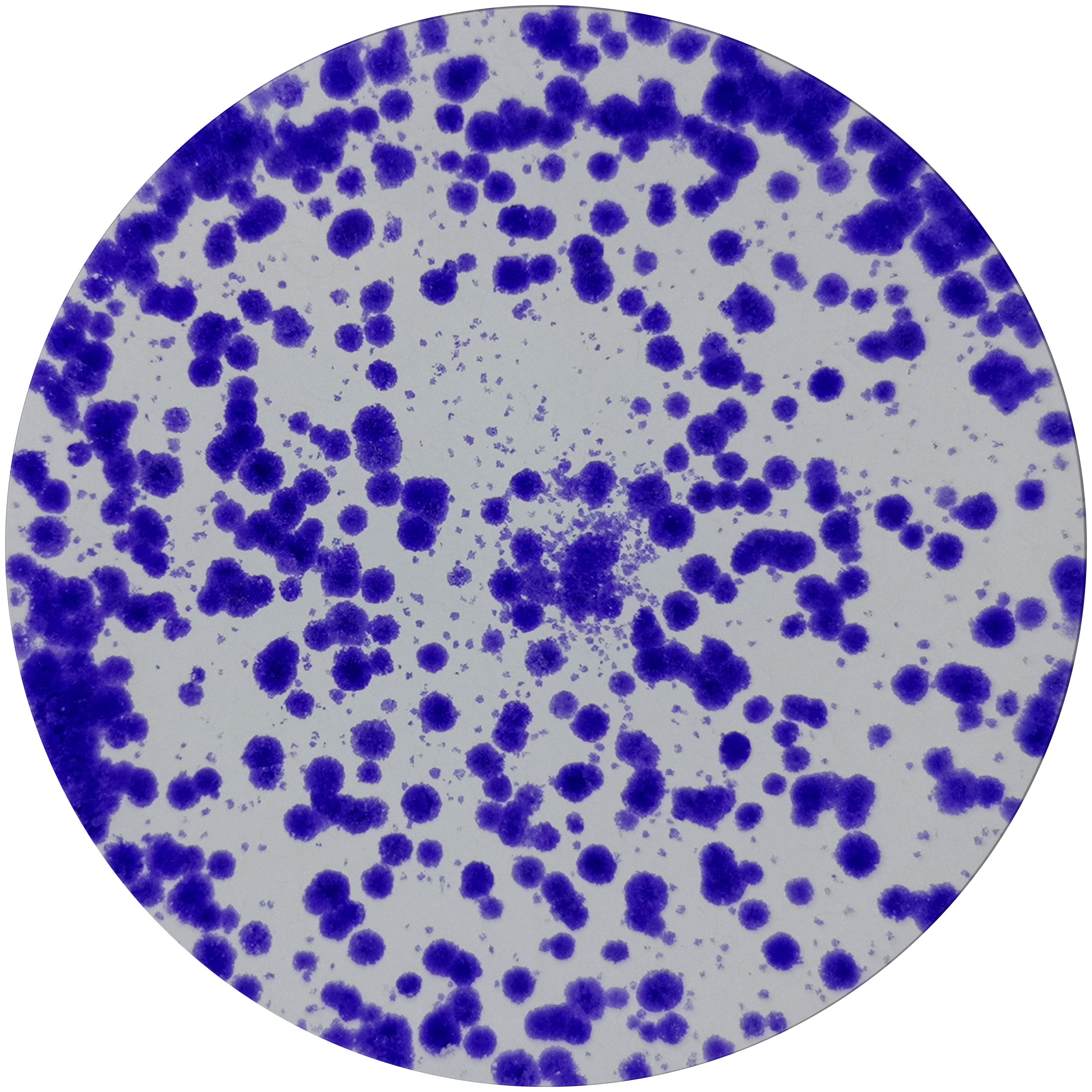

Supplement: Supplementary file 1 [file Data_Sheet_1.ZIP › Raw data1/Colony formation assay/B2-3.jpg]

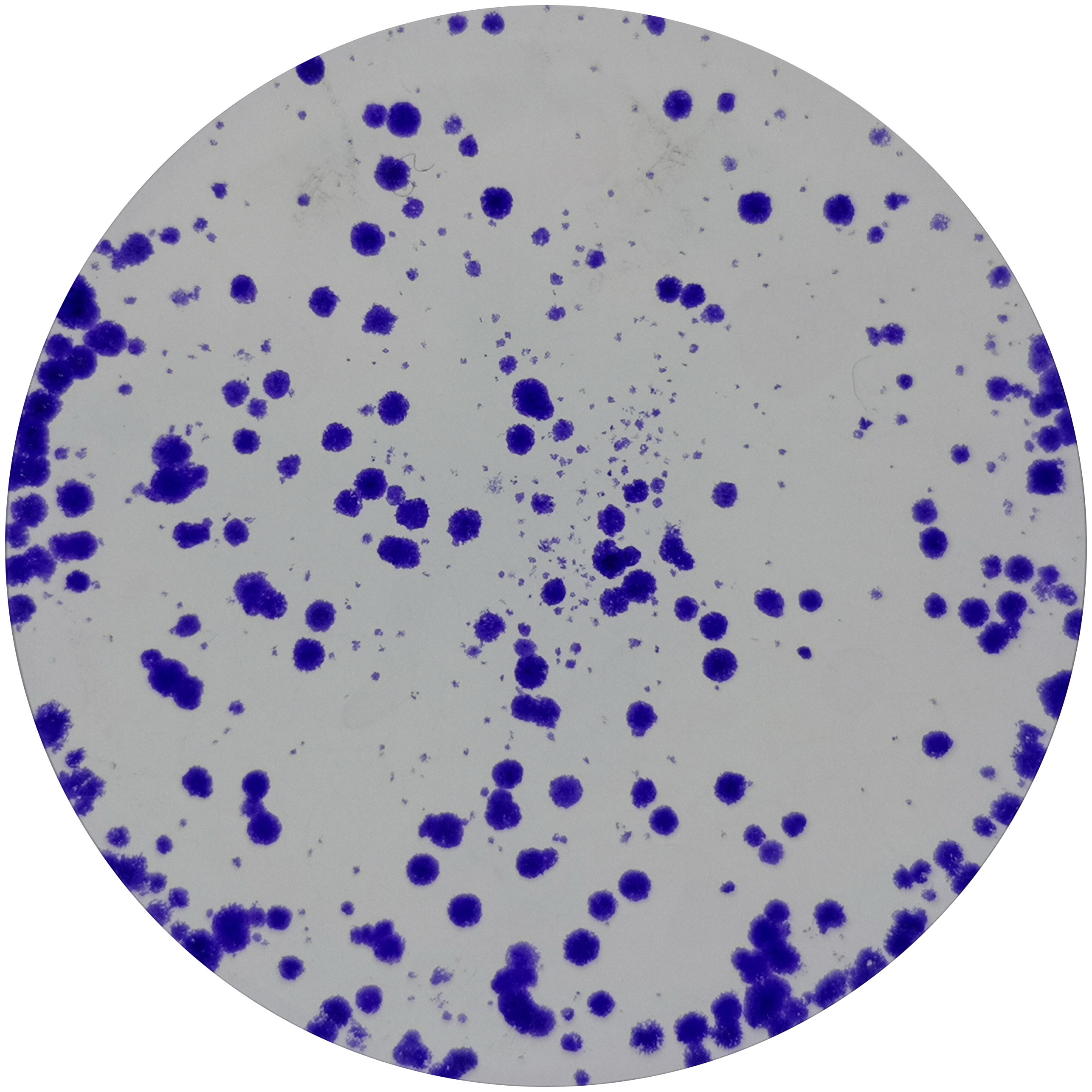

Supplement: Supplementary file 1 [file Data_Sheet_1.ZIP › Raw data1/Colony formation assay/C2-3.jpg]

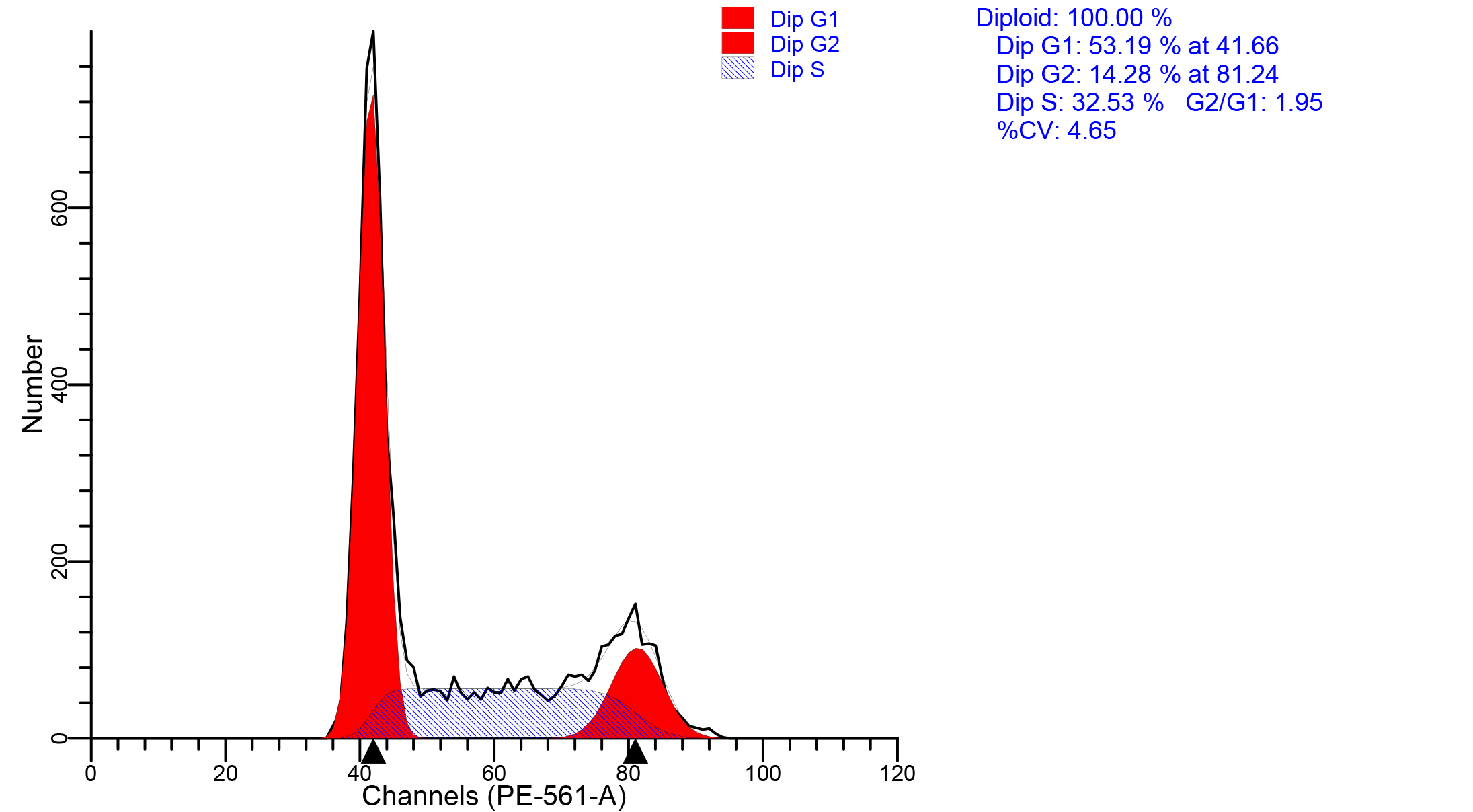

Supplement: Supplementary file 1 [file Data_Sheet_1.ZIP › Raw data1/Flow cytometry analysis/control1.tif]

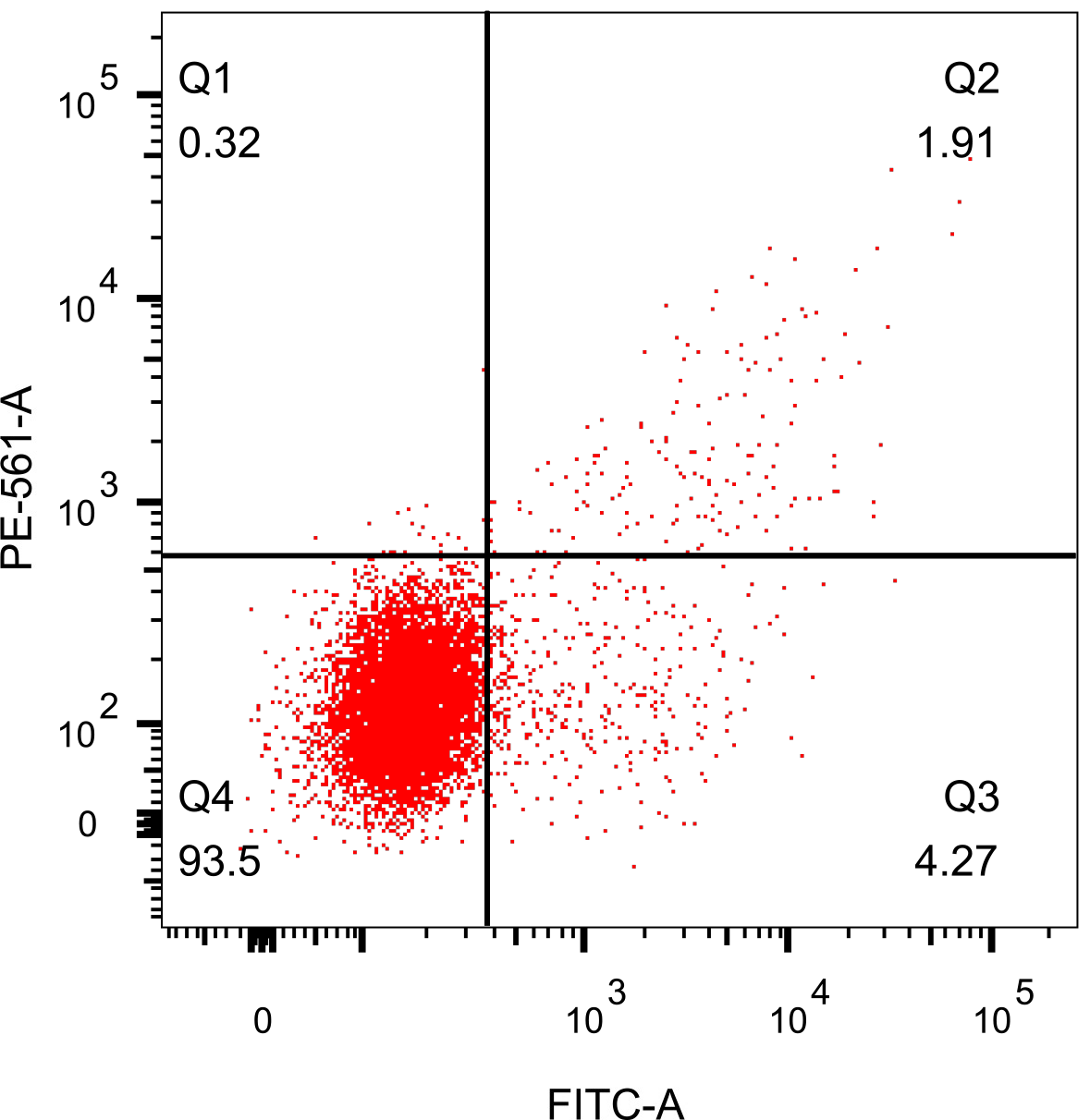

Supplement: Supplementary file 1 [file Data_Sheet_1.ZIP › Raw data1/Flow cytometry analysis/control2.tif]

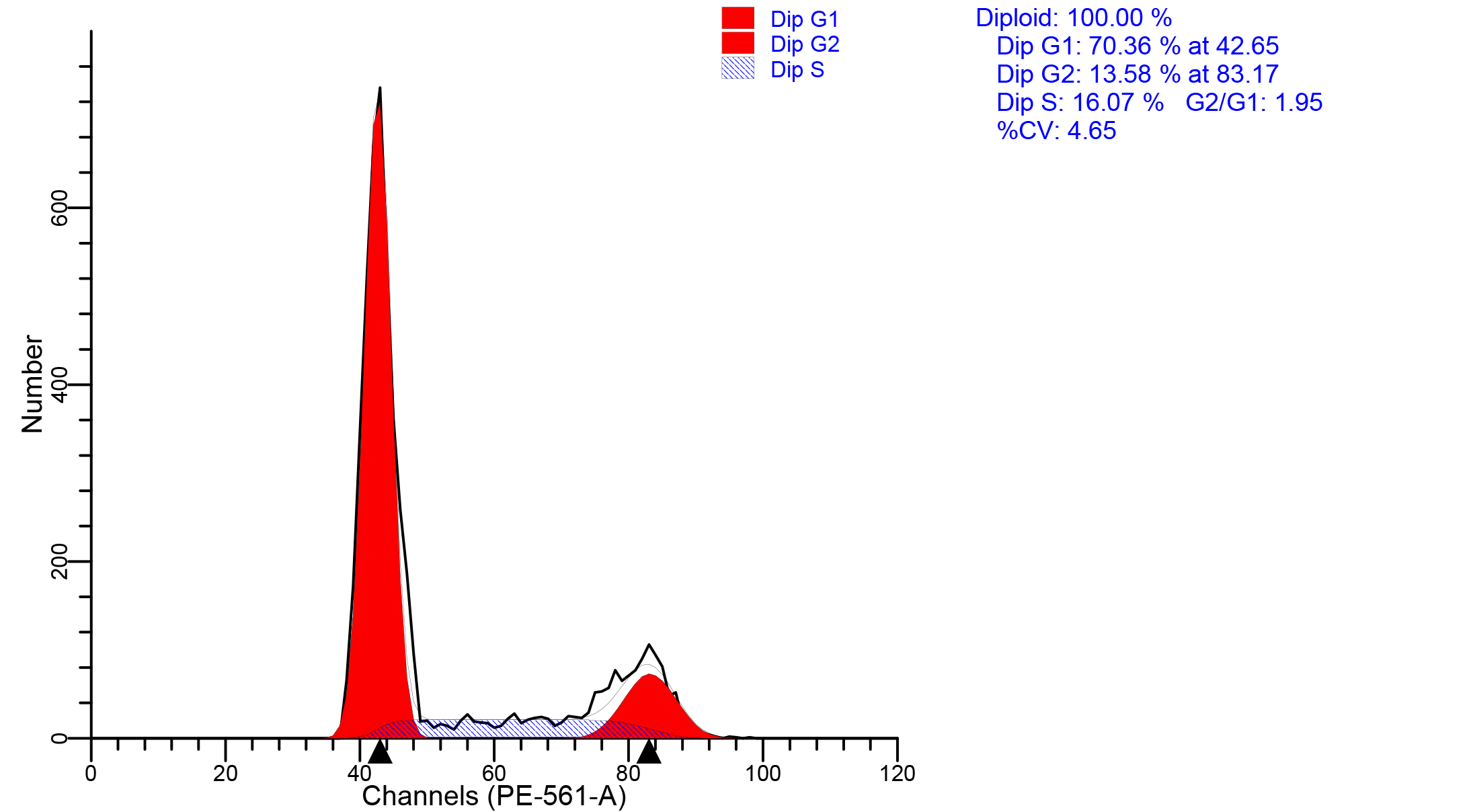

Supplement: Supplementary file 1 [file Data_Sheet_1.ZIP › Raw data1/Flow cytometry analysis/siCAPN2-1.tif]

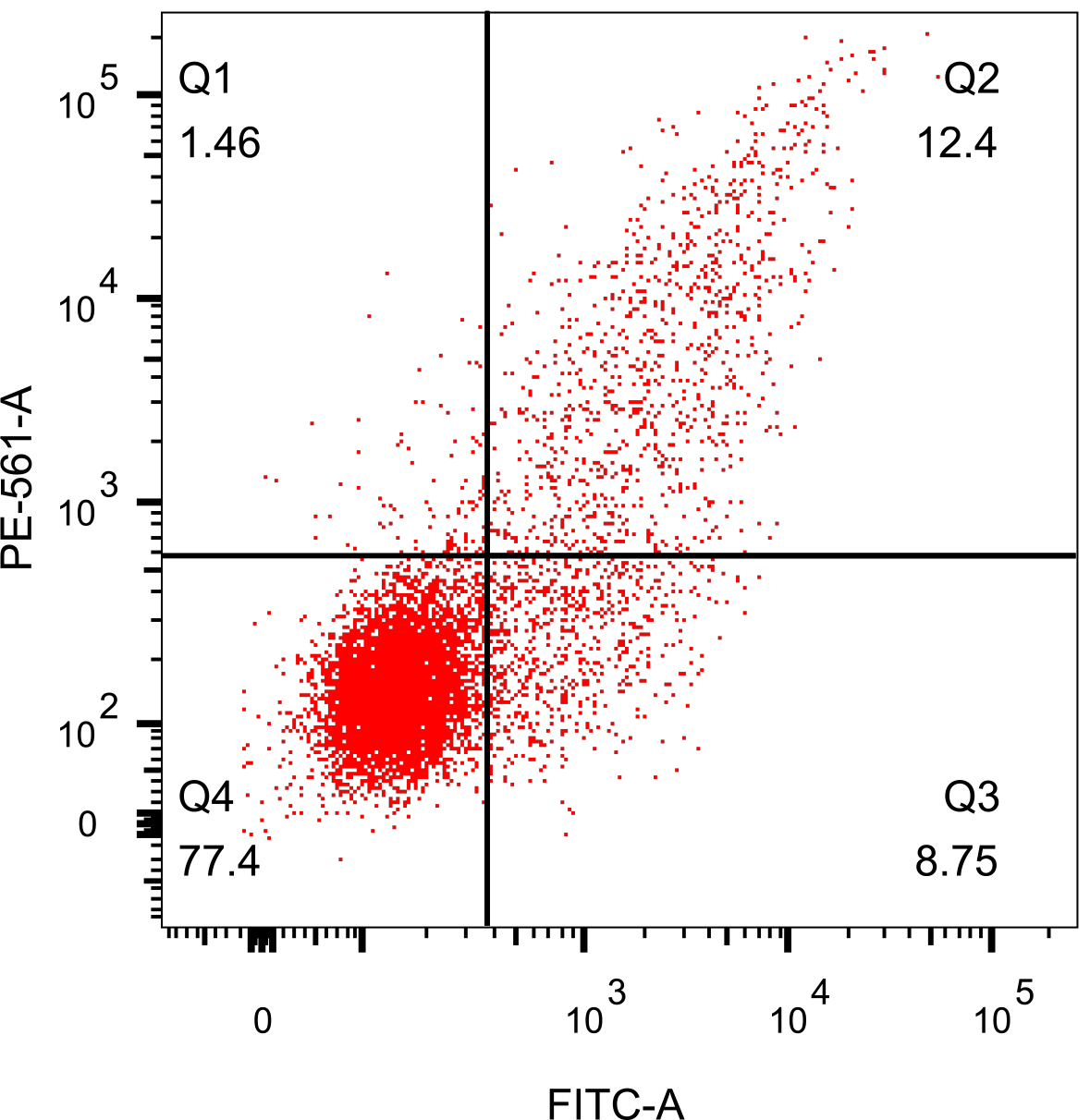

Supplement: Supplementary file 1 [file Data_Sheet_1.ZIP › Raw data1/Flow cytometry analysis/siCAPN2-2.tif]

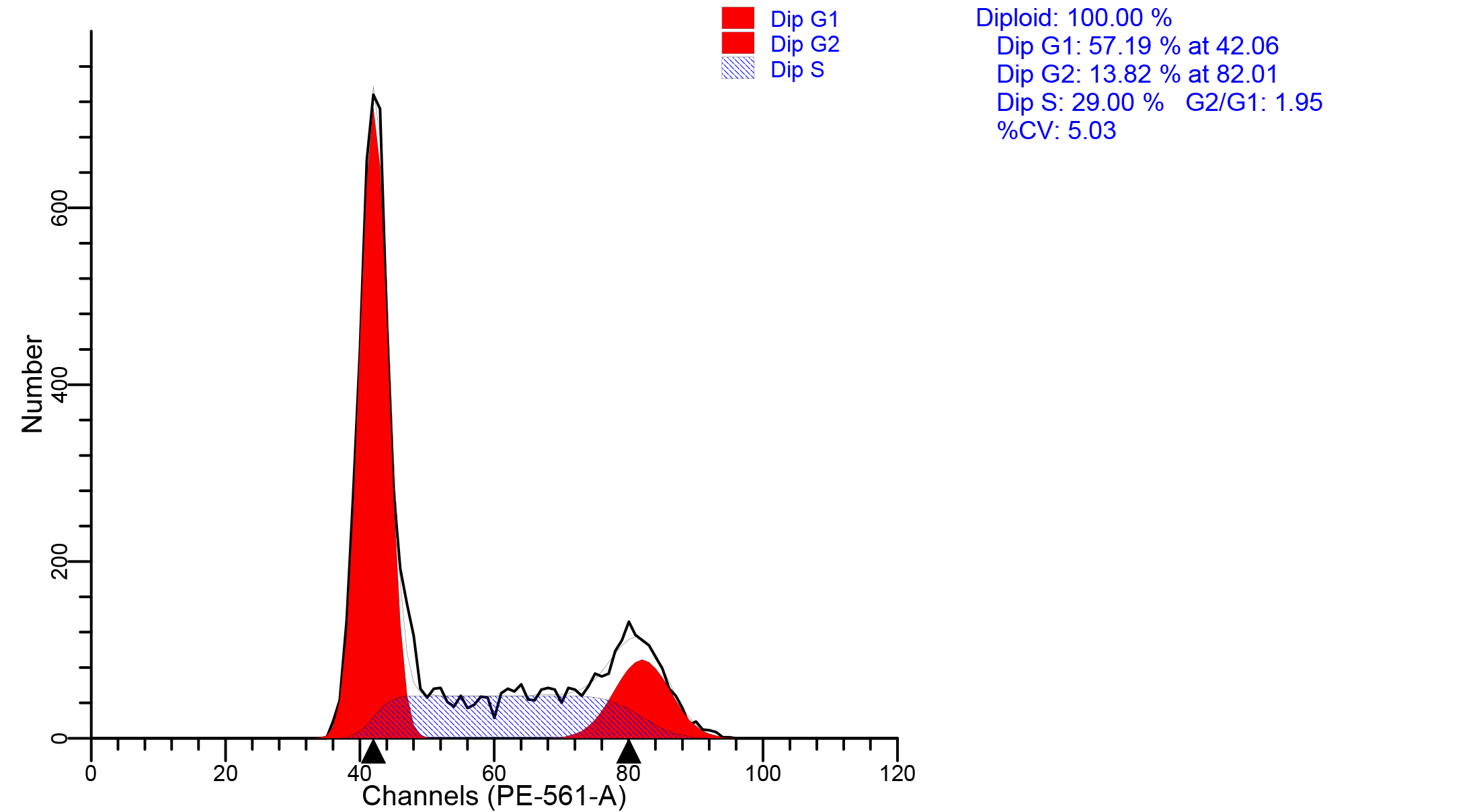

Supplement: Supplementary file 1 [file Data_Sheet_1.ZIP › Raw data1/Flow cytometry analysis/siNC-1.tif]

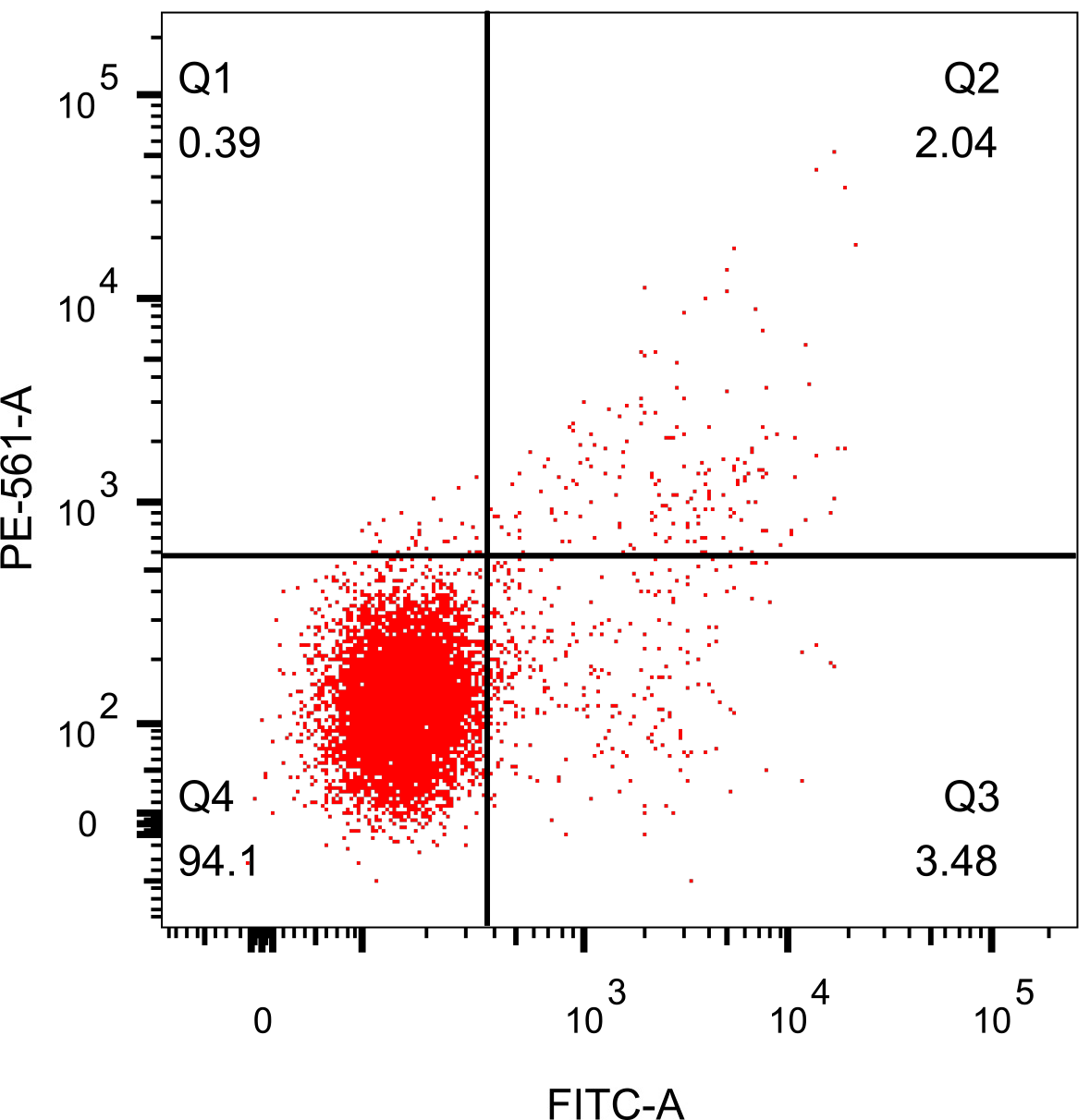

Supplement: Supplementary file 1 [file Data_Sheet_1.ZIP › Raw data1/Flow cytometry analysis/siNC-2.tif]

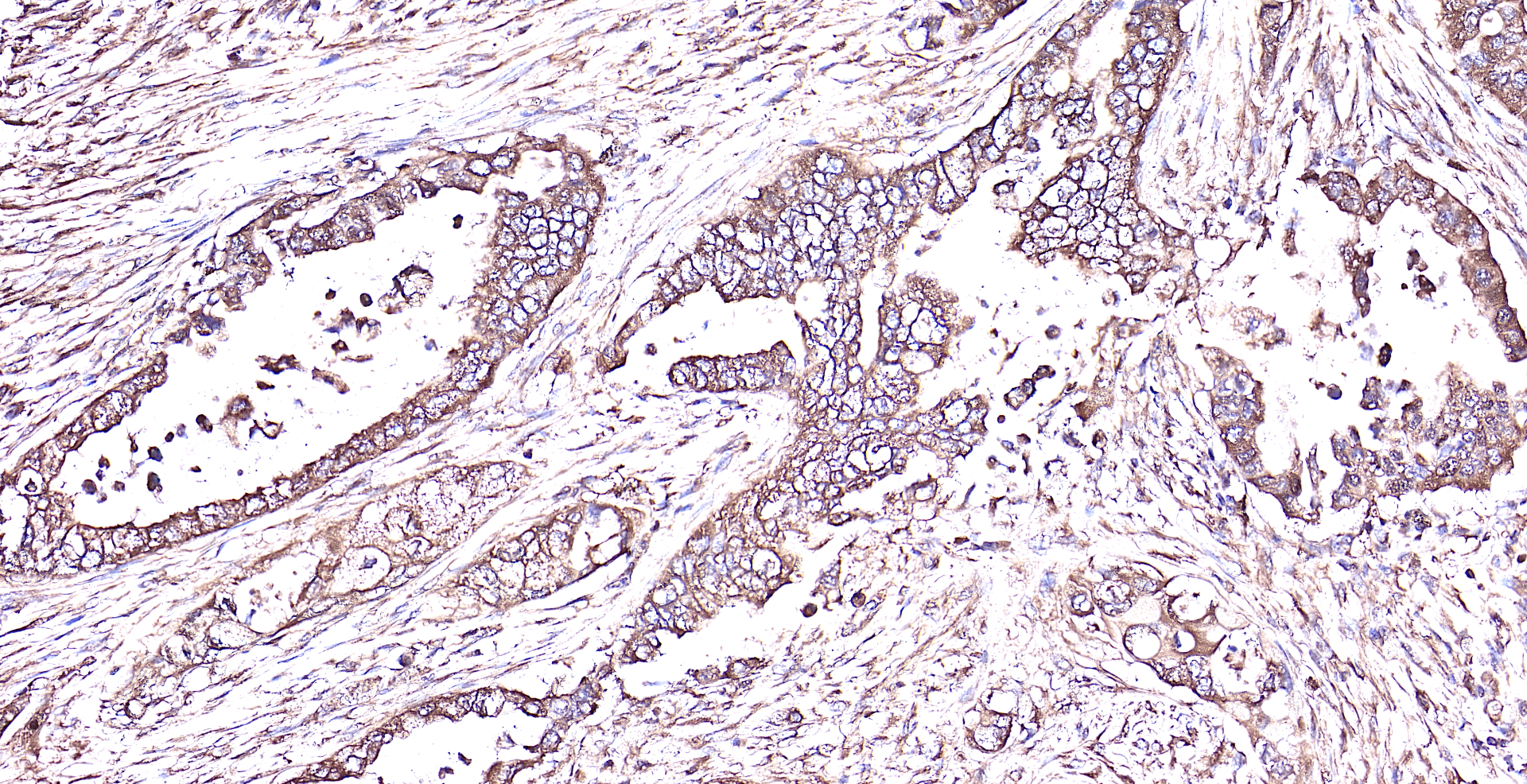

Supplement: Supplementary file 1 [file Data_Sheet_1.ZIP › Raw data1/Immunohistochemistry/A05-20.jpg]

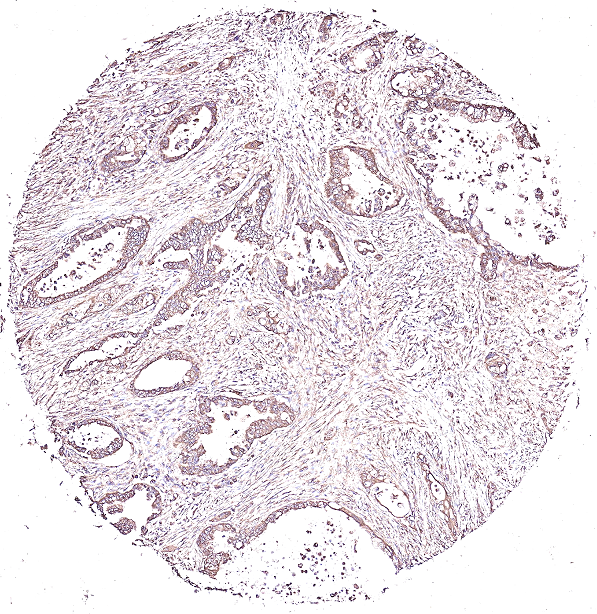

Supplement: Supplementary file 1 [file Data_Sheet_1.ZIP › Raw data1/Immunohistochemistry/A05.png]

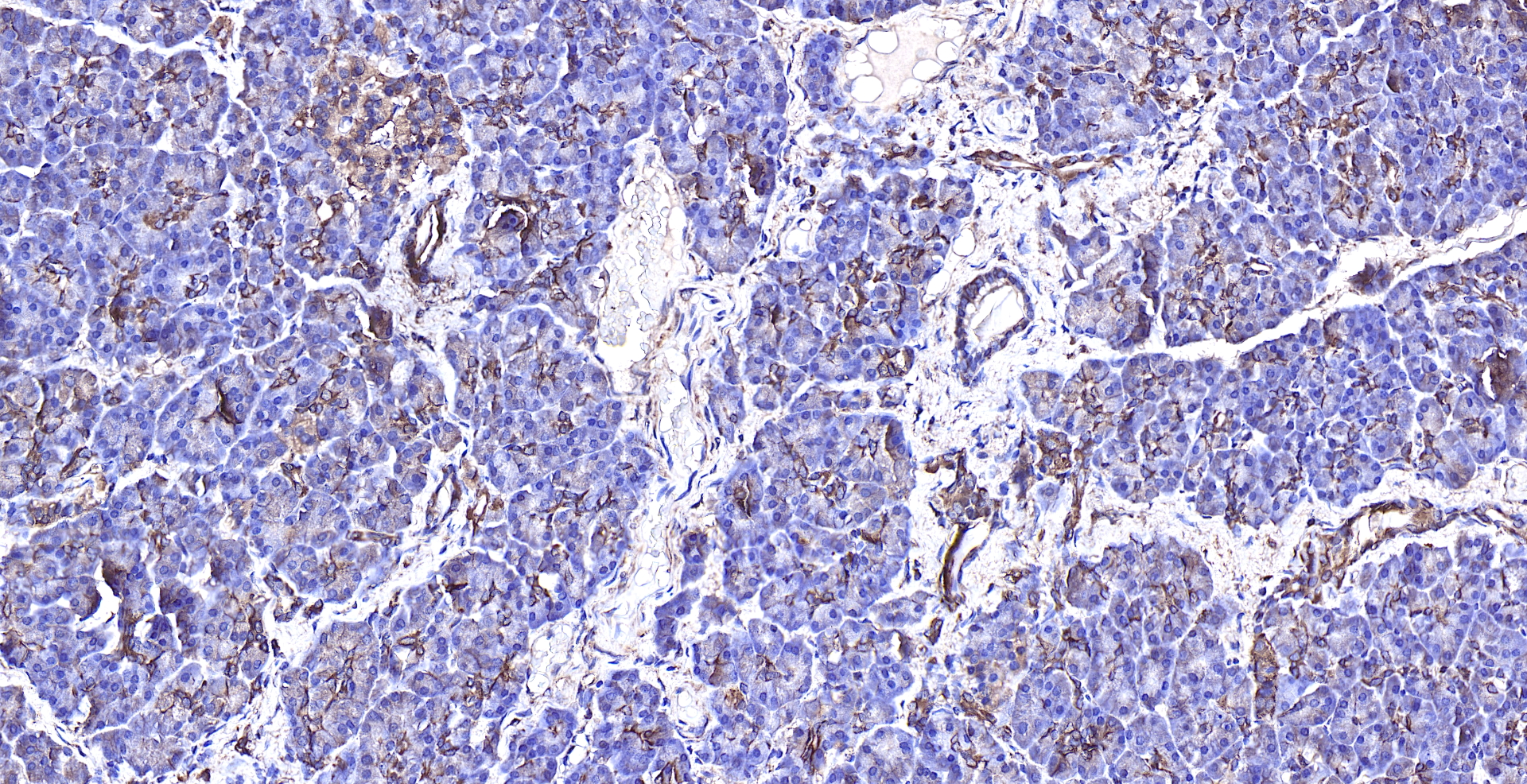

Supplement: Supplementary file 1 [file Data_Sheet_1.ZIP › Raw data1/Immunohistochemistry/B10-20.jpg]

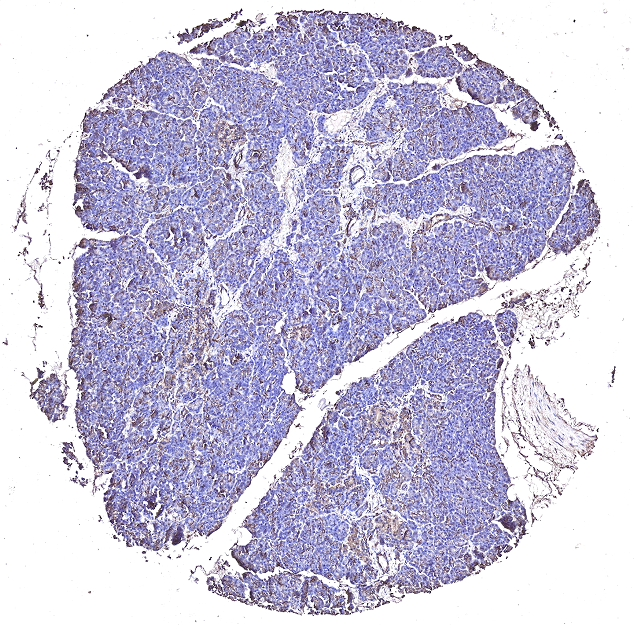

Supplement: Supplementary file 1 [file Data_Sheet_1.ZIP › Raw data1/Immunohistochemistry/B10.png]

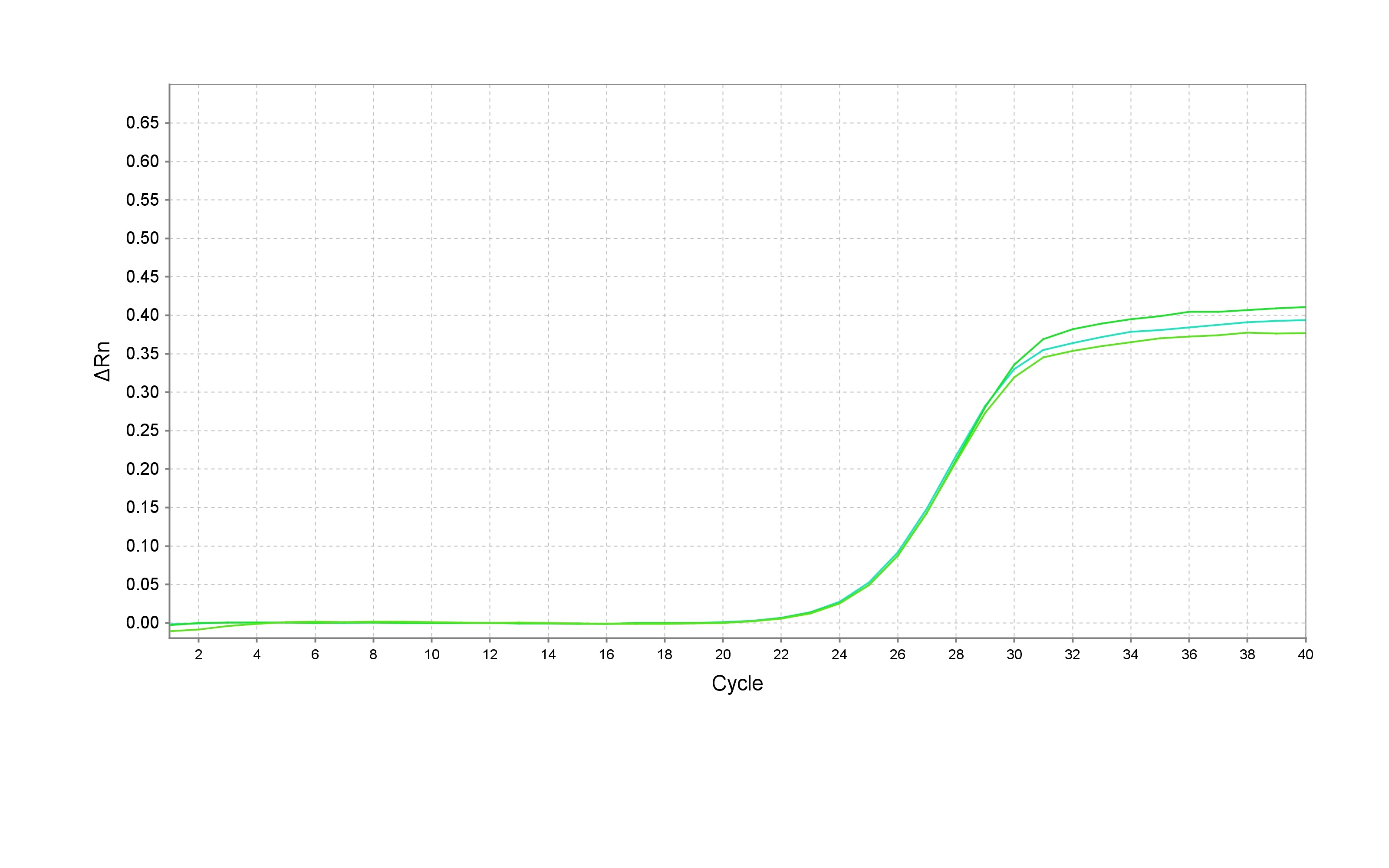

Supplement: Supplementary file 1 [file Data_Sheet_1.ZIP › Raw data1/RT-qPCR/└⌐╘÷╟·╧▀/CANP2 (A1 ó┘).jpg]

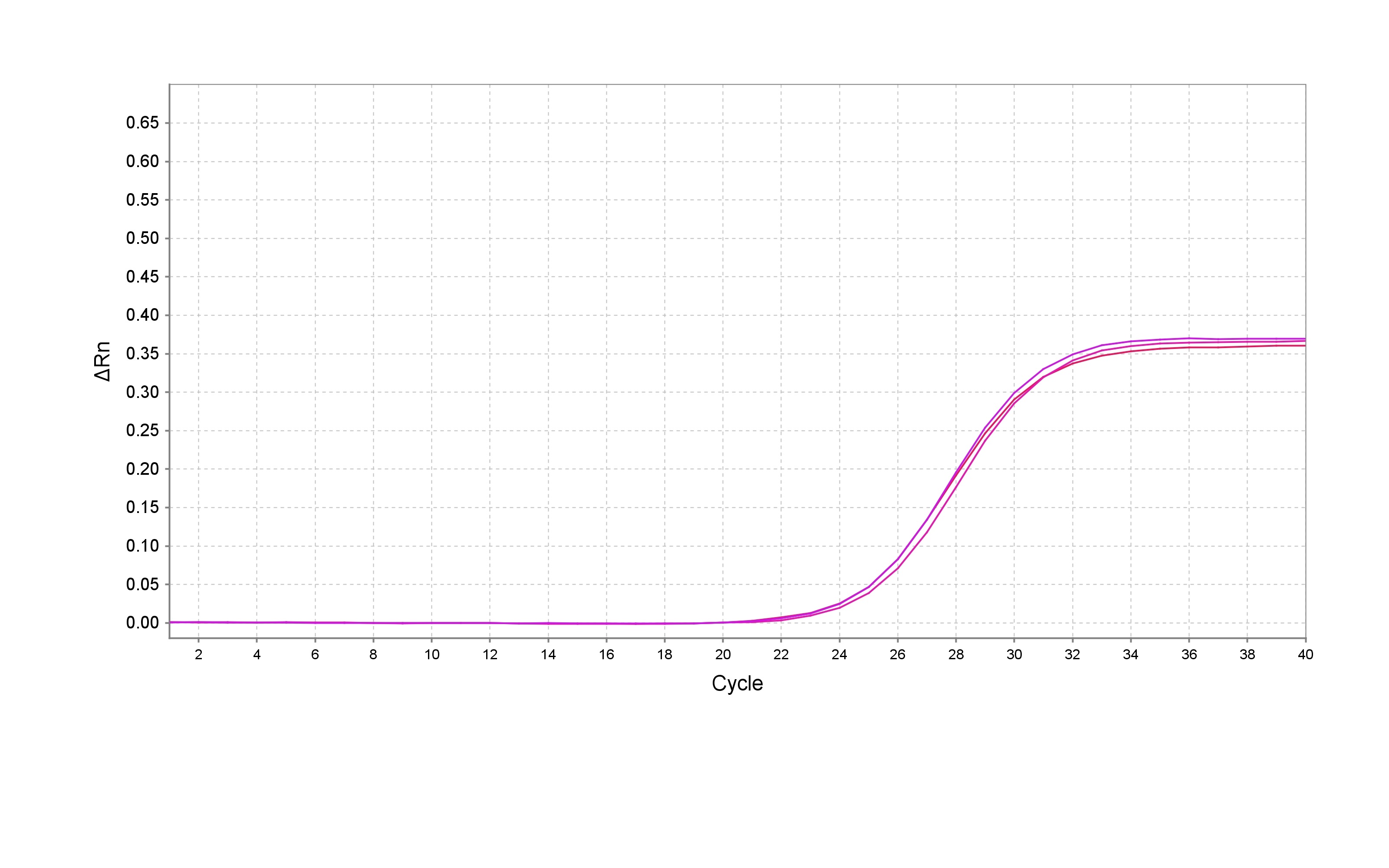

Supplement: Supplementary file 1 [file Data_Sheet_1.ZIP › Raw data1/RT-qPCR/└⌐╘÷╟·╧▀/CANP2 (A1 ó┌).jpg]

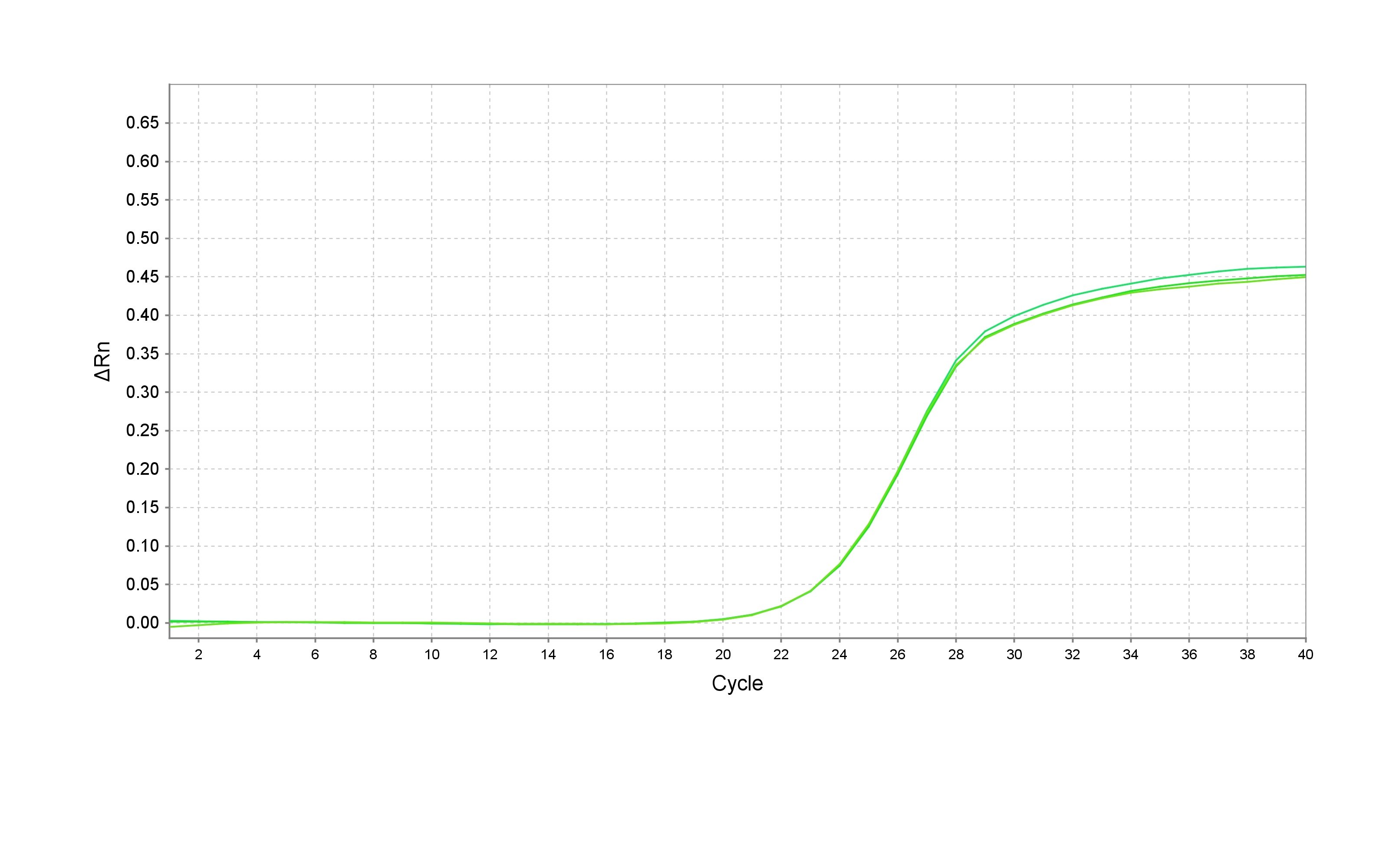

Supplement: Supplementary file 1 [file Data_Sheet_1.ZIP › Raw data1/RT-qPCR/└⌐╘÷╟·╧▀/CANP2 (A1 ó█).jpg]

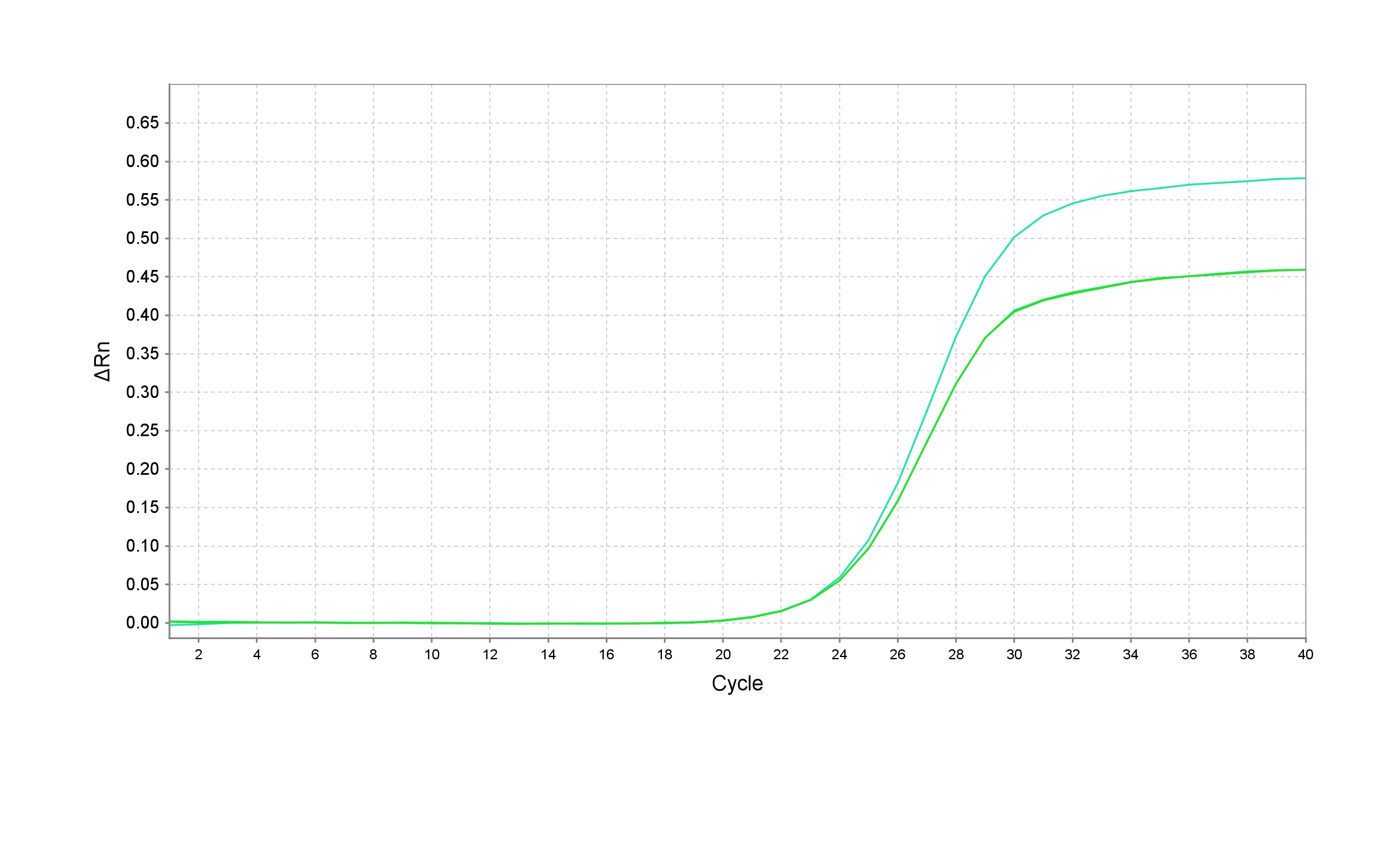

Supplement: Supplementary file 1 [file Data_Sheet_1.ZIP › Raw data1/RT-qPCR/└⌐╘÷╟·╧▀/CANP2 (B1 ó┘).jpg]

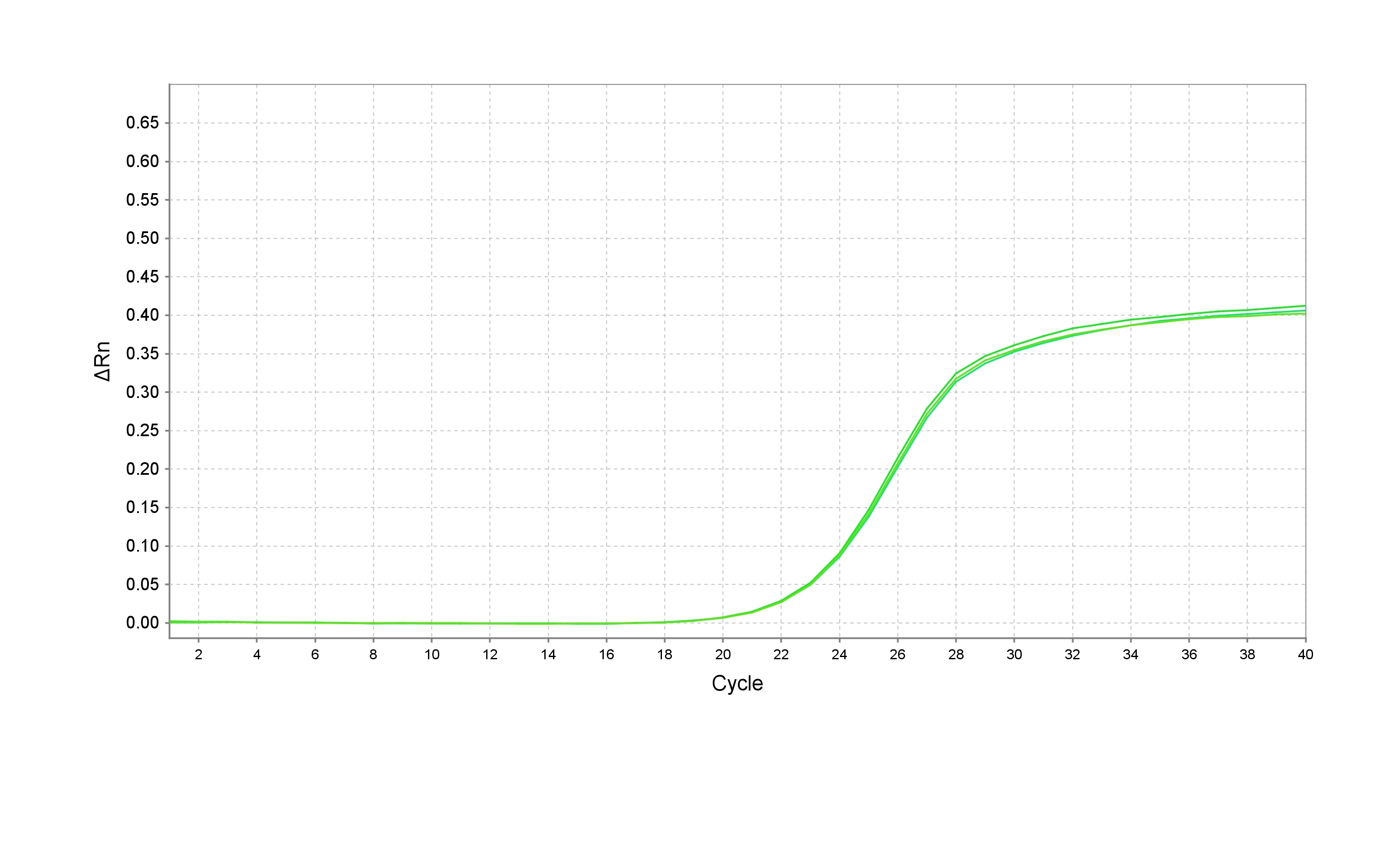

Supplement: Supplementary file 1 [file Data_Sheet_1.ZIP › Raw data1/RT-qPCR/└⌐╘÷╟·╧▀/CANP2 (B1 ó┌).jpg]

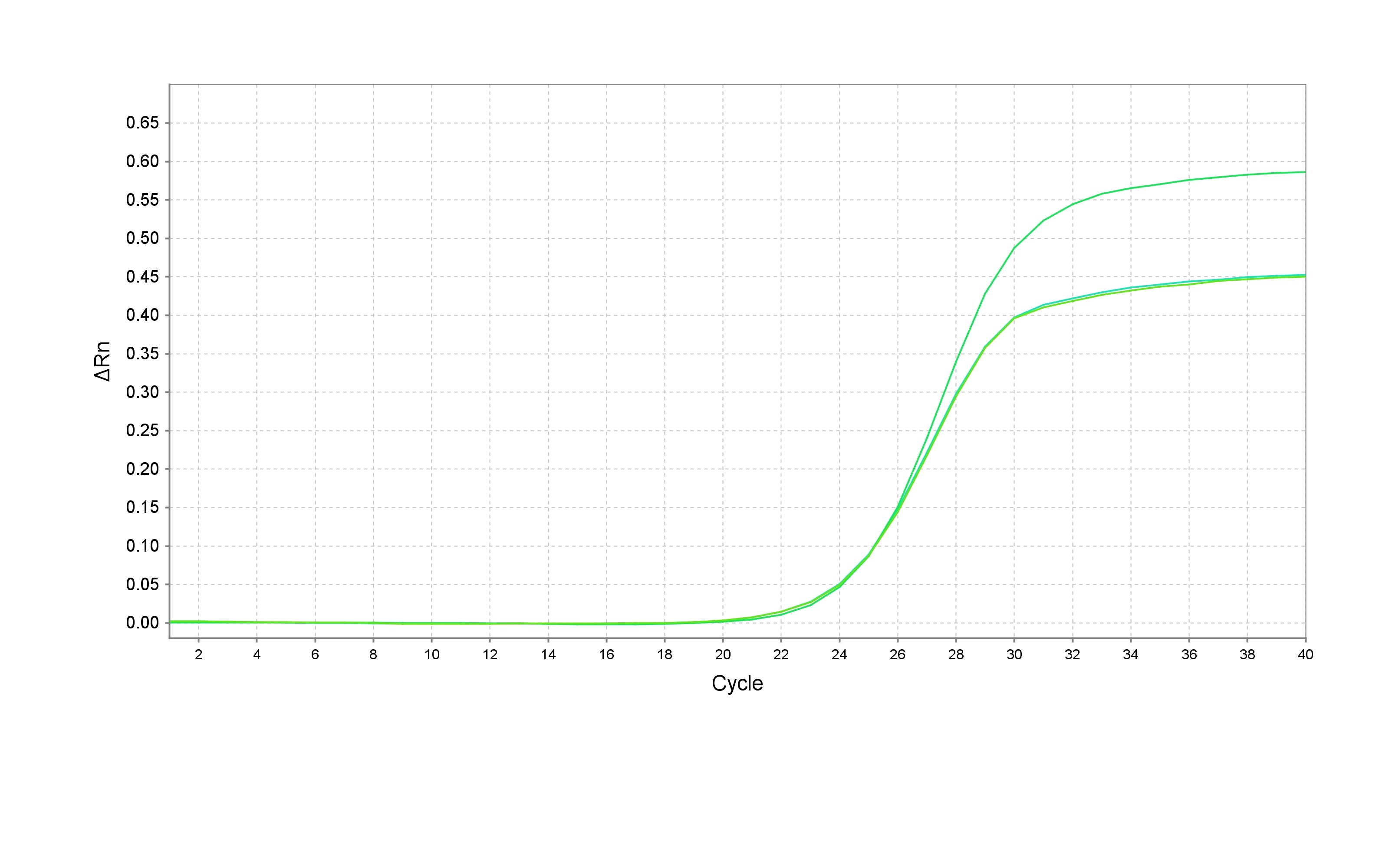

Supplement: Supplementary file 1 [file Data_Sheet_1.ZIP › Raw data1/RT-qPCR/└⌐╘÷╟·╧▀/CANP2 (B1 ó█).jpg]

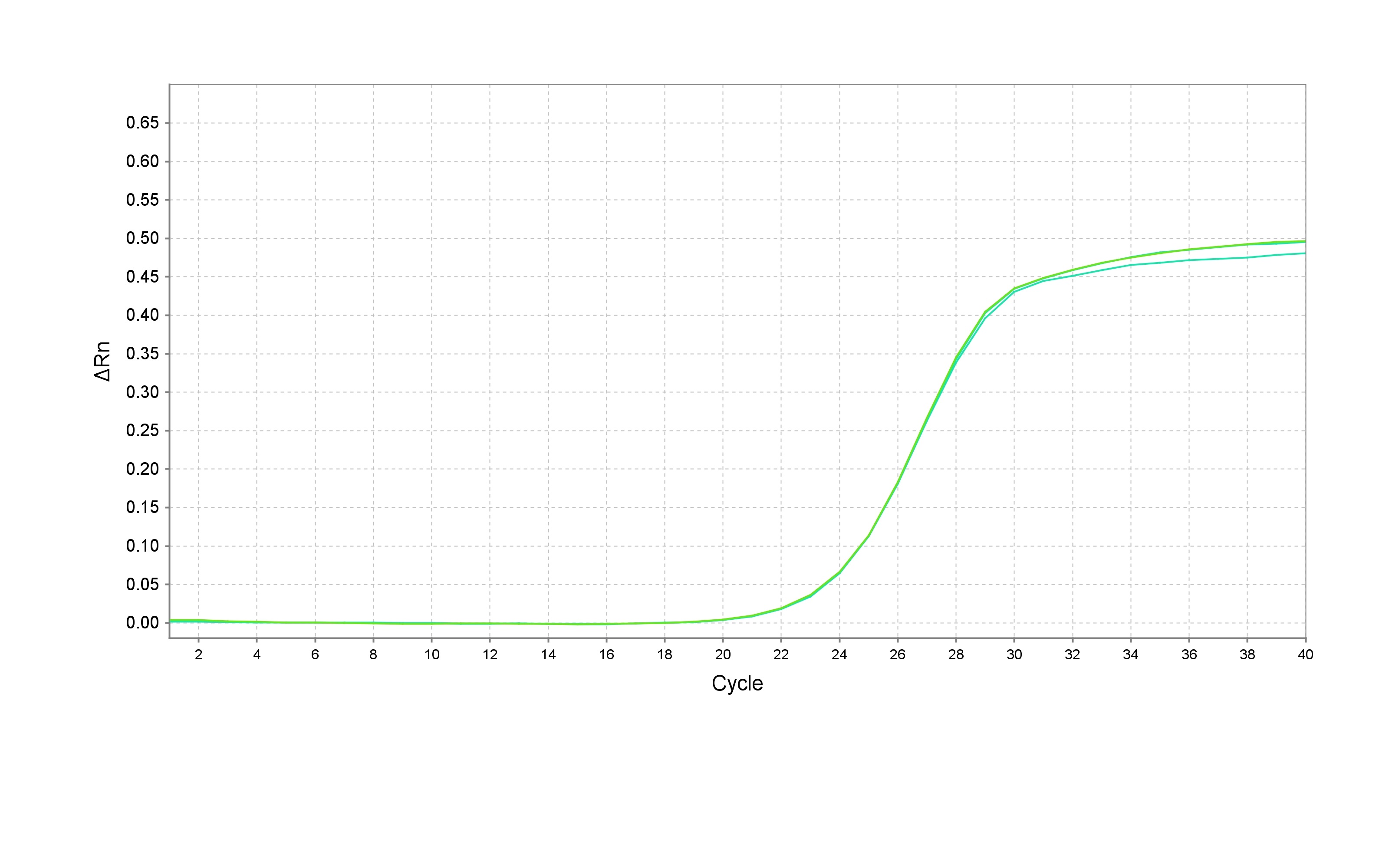

Supplement: Supplementary file 1 [file Data_Sheet_1.ZIP › Raw data1/RT-qPCR/└⌐╘÷╟·╧▀/CANP2 (C1 ó┘).jpg]

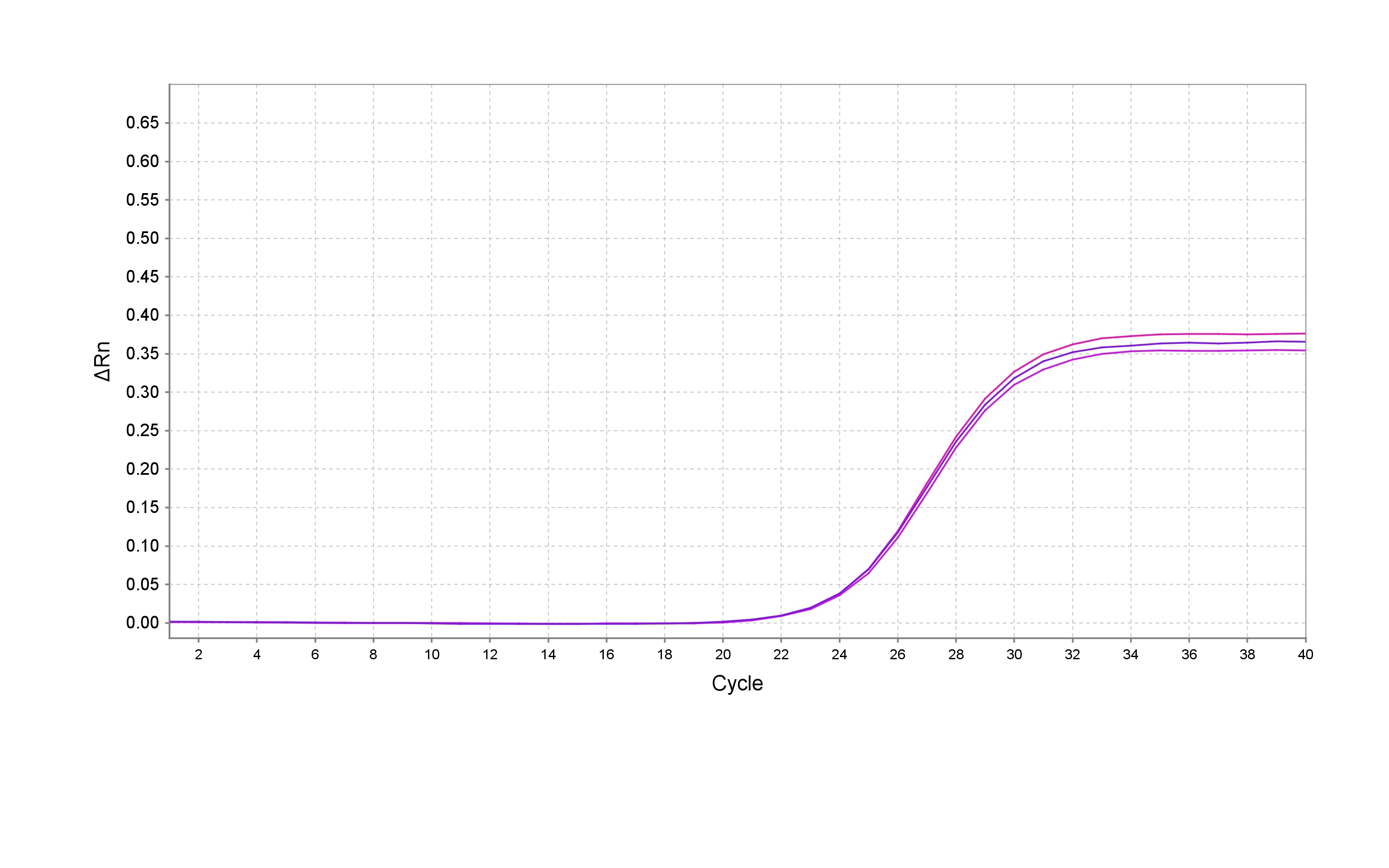

Supplement: Supplementary file 1 [file Data_Sheet_1.ZIP › Raw data1/RT-qPCR/└⌐╘÷╟·╧▀/CANP2 (C1 ó┌).jpg]

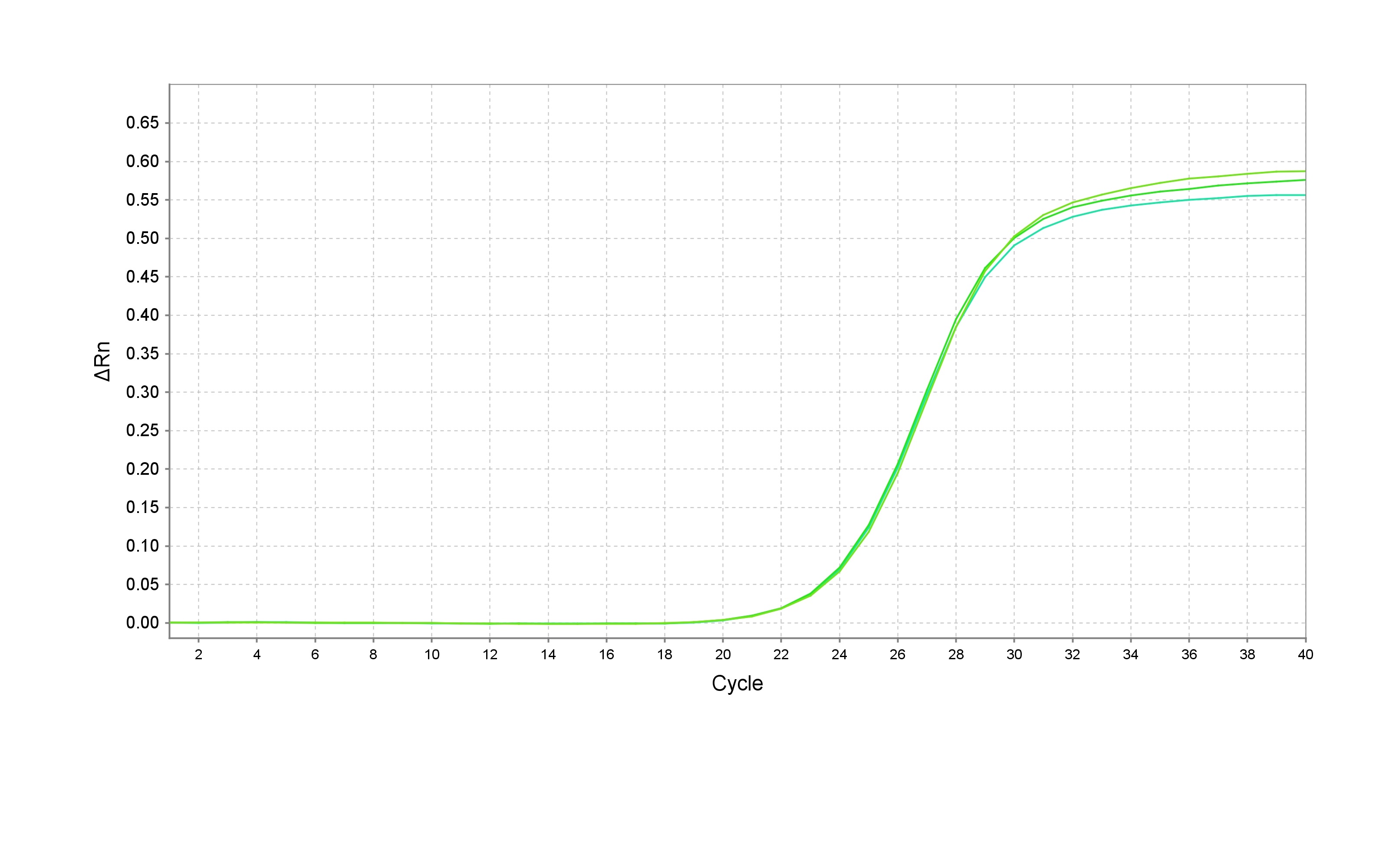

Supplement: Supplementary file 1 [file Data_Sheet_1.ZIP › Raw data1/RT-qPCR/└⌐╘÷╟·╧▀/CANP2 (C1 ó█).jpg]

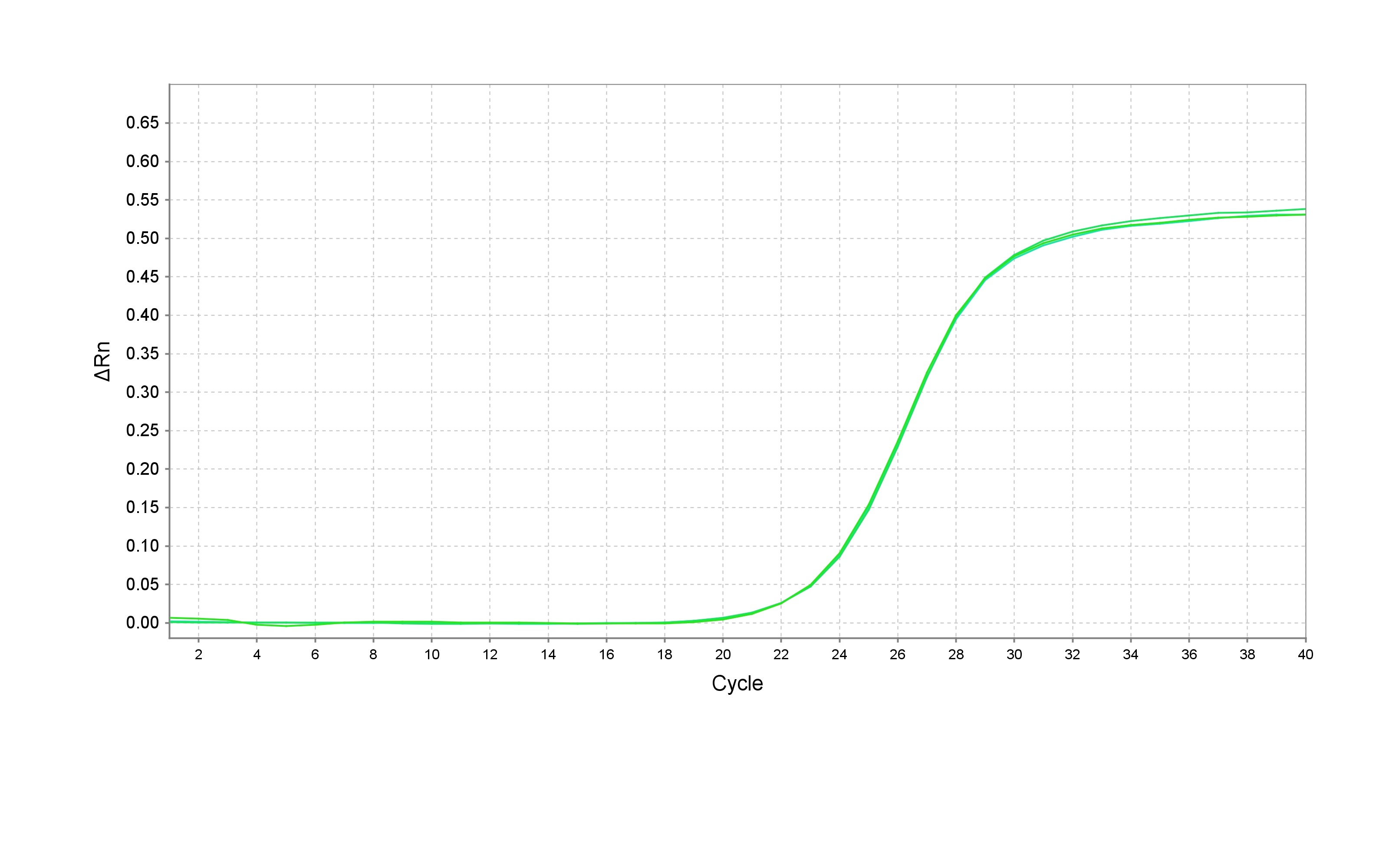

Supplement: Supplementary file 1 [file Data_Sheet_1.ZIP › Raw data1/RT-qPCR/└⌐╘÷╟·╧▀/CANP2 (D1 ó┘).jpg]

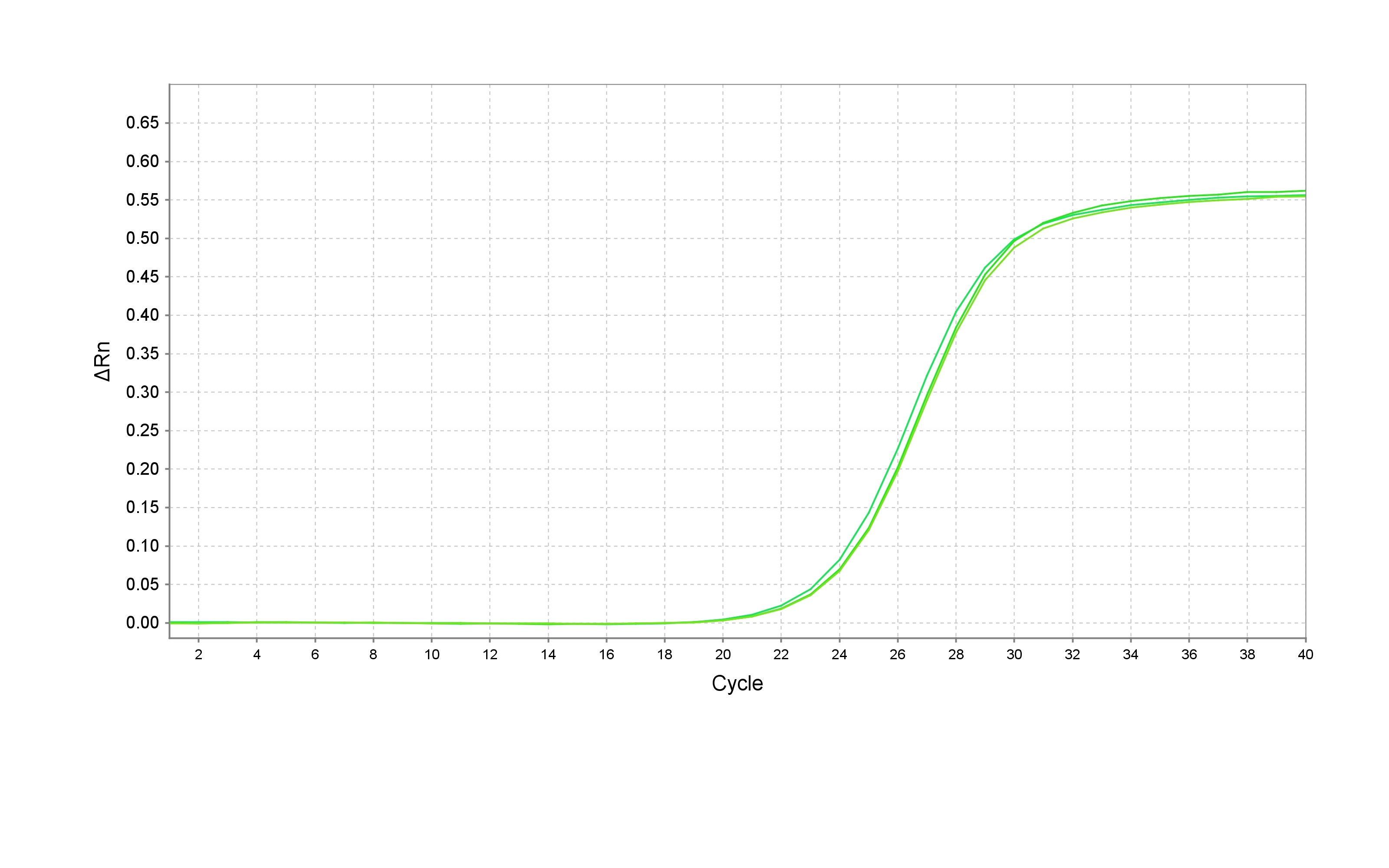

Supplement: Supplementary file 1 [file Data_Sheet_1.ZIP › Raw data1/RT-qPCR/└⌐╘÷╟·╧▀/CANP2 (D1 ó┌).jpg]

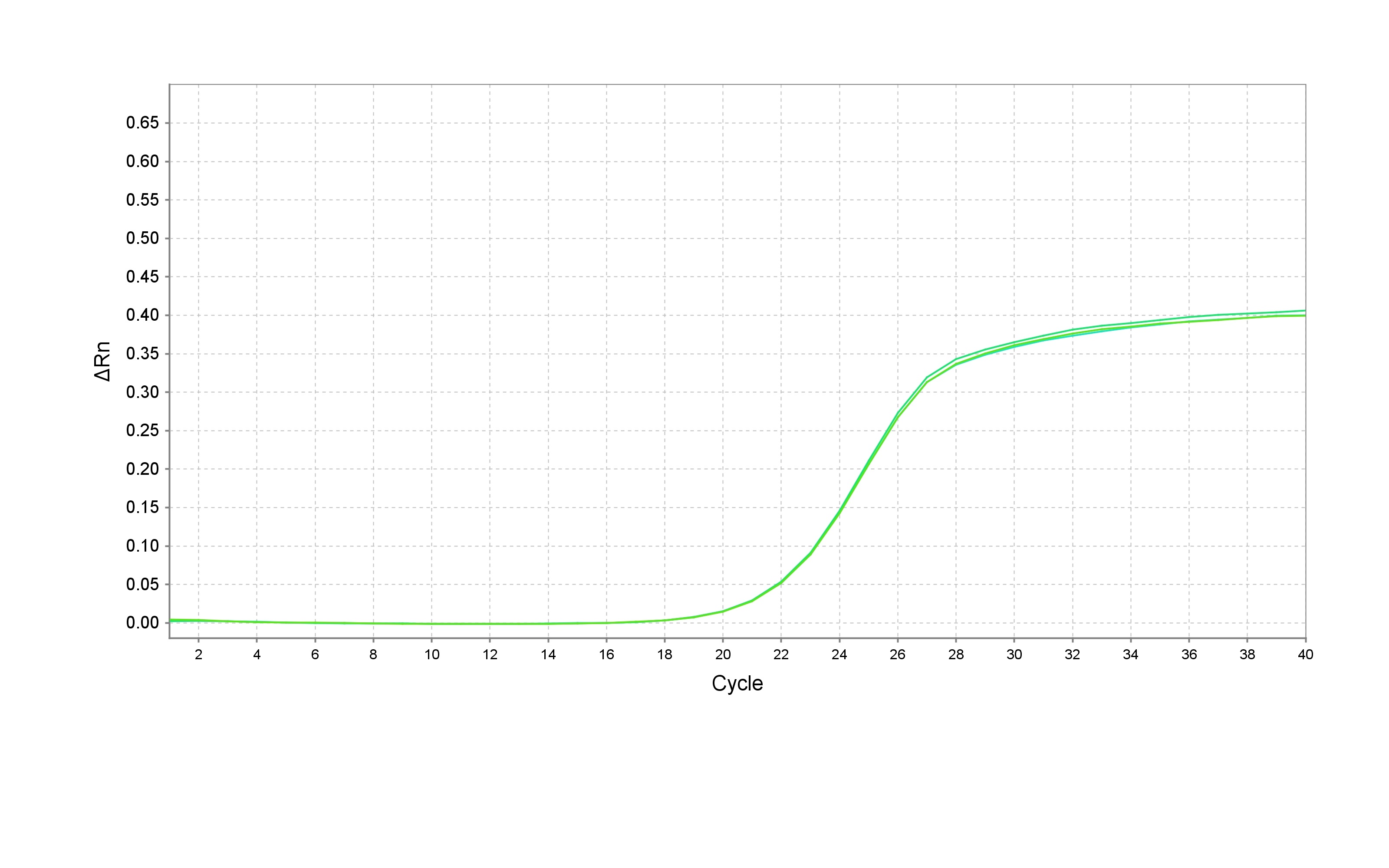

Supplement: Supplementary file 1 [file Data_Sheet_1.ZIP › Raw data1/RT-qPCR/└⌐╘÷╟·╧▀/CANP2 (D1 ó█).jpg]

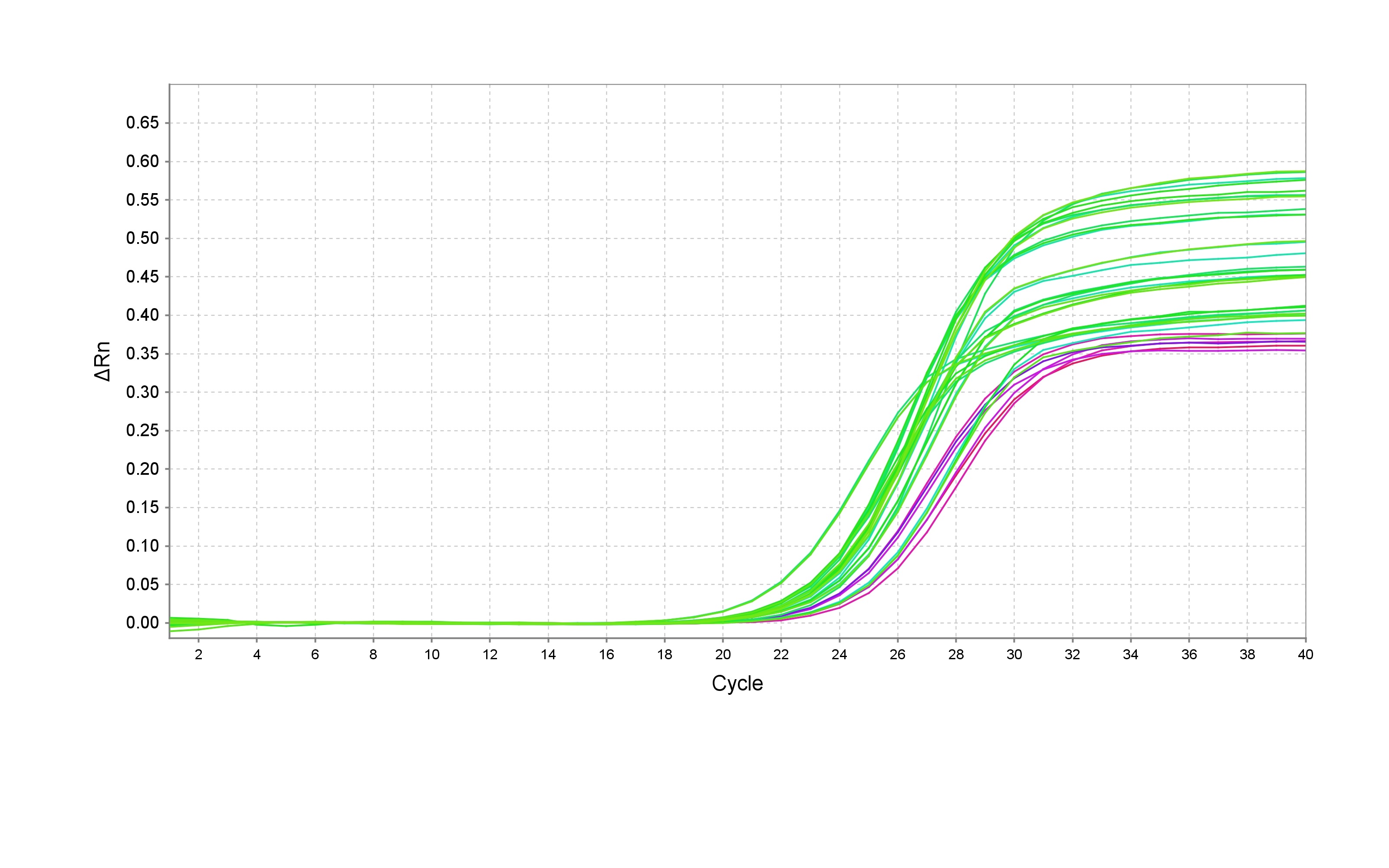

Supplement: Supplementary file 1 [file Data_Sheet_1.ZIP › Raw data1/RT-qPCR/└⌐╘÷╟·╧▀/CANP2.jpg]

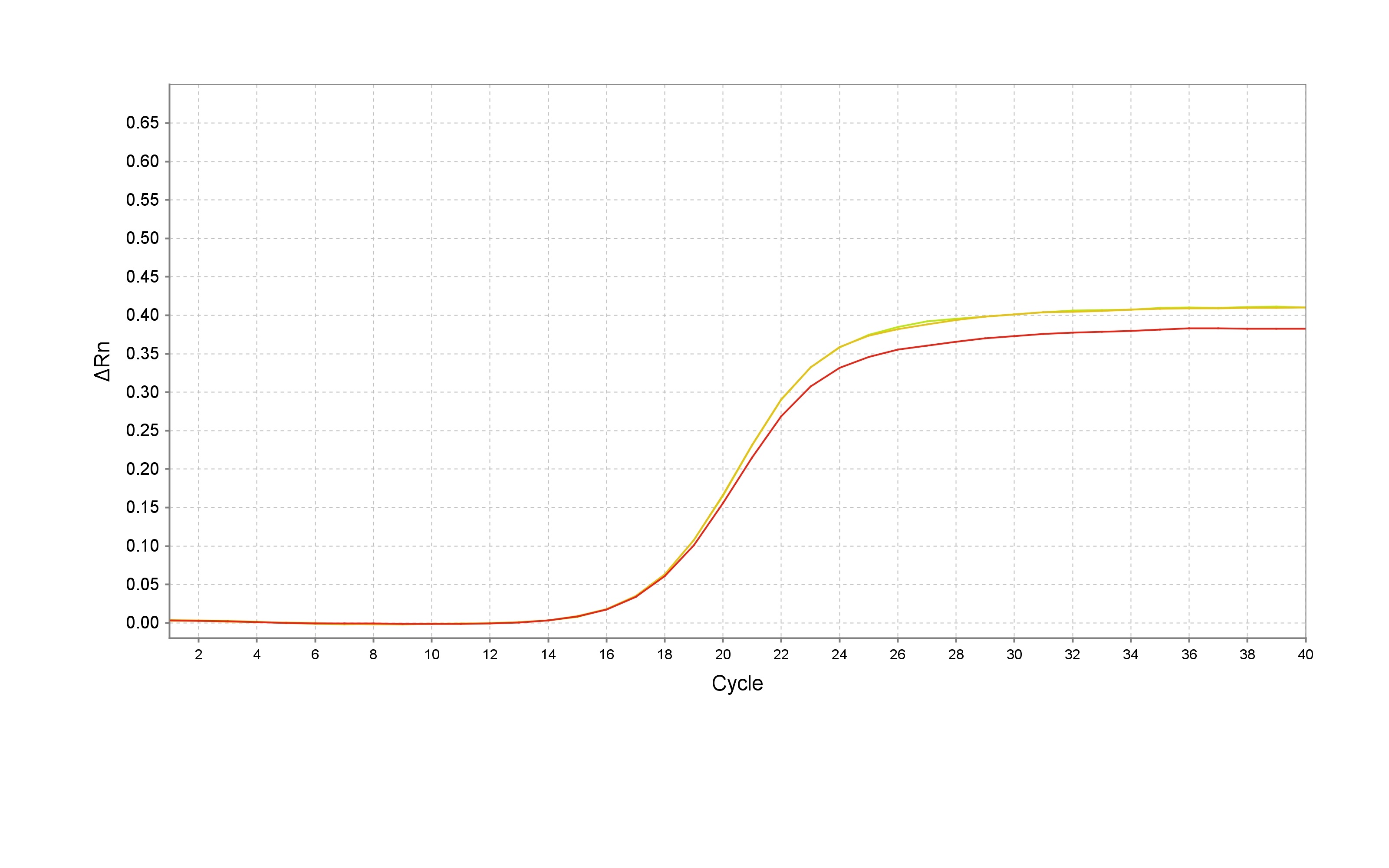

Supplement: Supplementary file 1 [file Data_Sheet_1.ZIP › Raw data1/RT-qPCR/└⌐╘÷╟·╧▀/GAPDH (A1 ó┘).jpg]

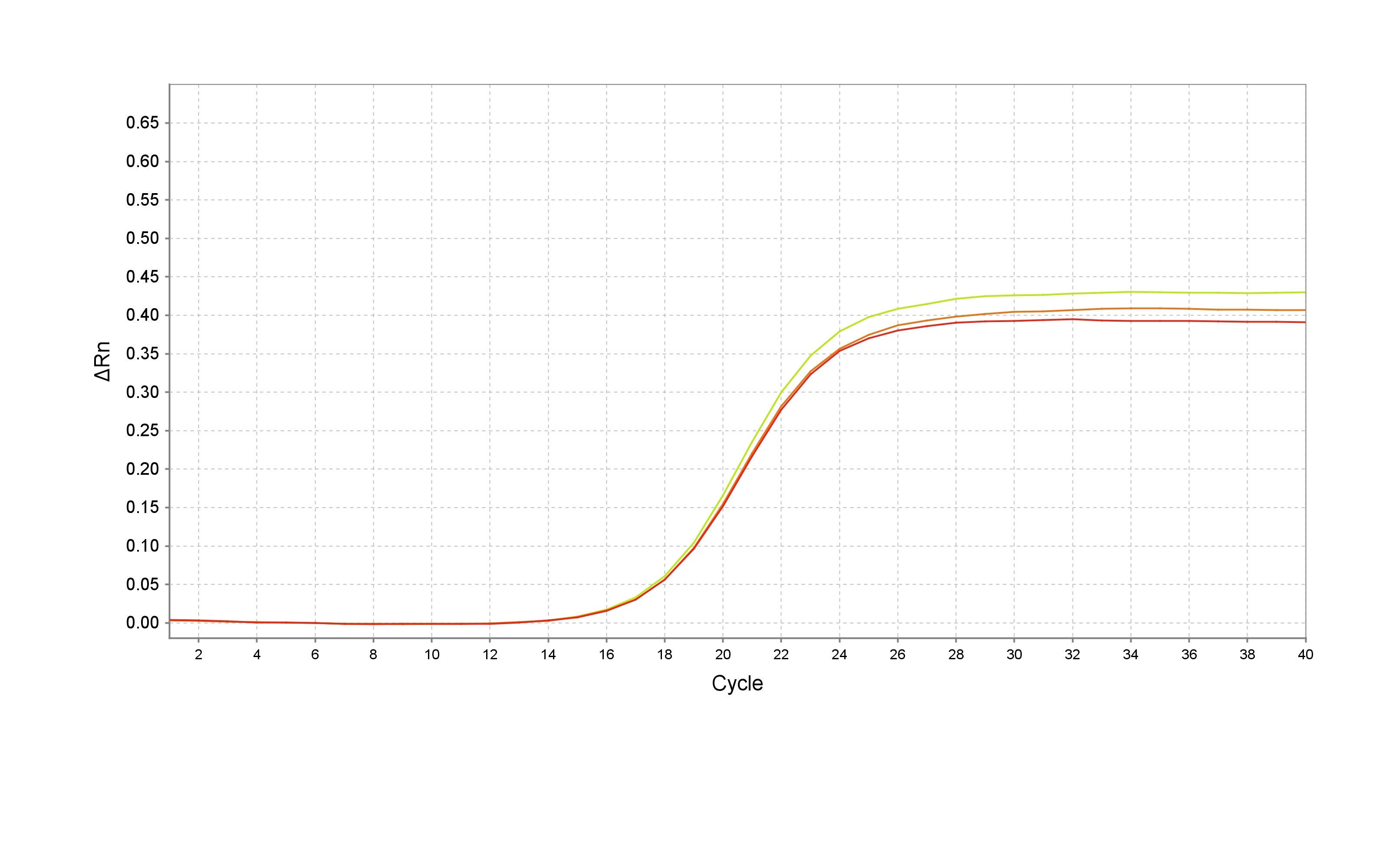

Supplement: Supplementary file 1 [file Data_Sheet_1.ZIP › Raw data1/RT-qPCR/└⌐╘÷╟·╧▀/GAPDH (A1 ó┌).jpg]

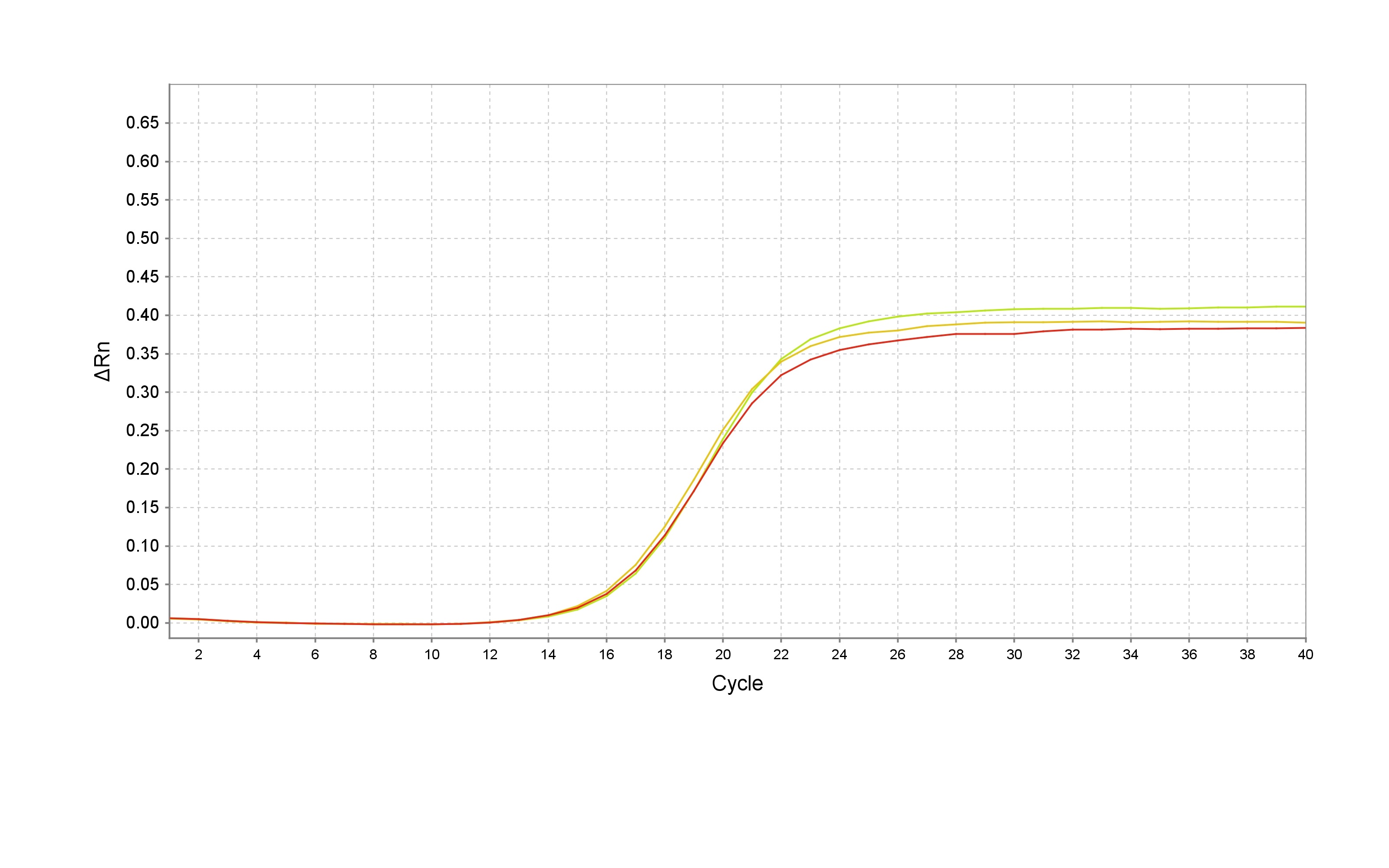

Supplement: Supplementary file 1 [file Data_Sheet_1.ZIP › Raw data1/RT-qPCR/└⌐╘÷╟·╧▀/GAPDH (A1 ó█).jpg]

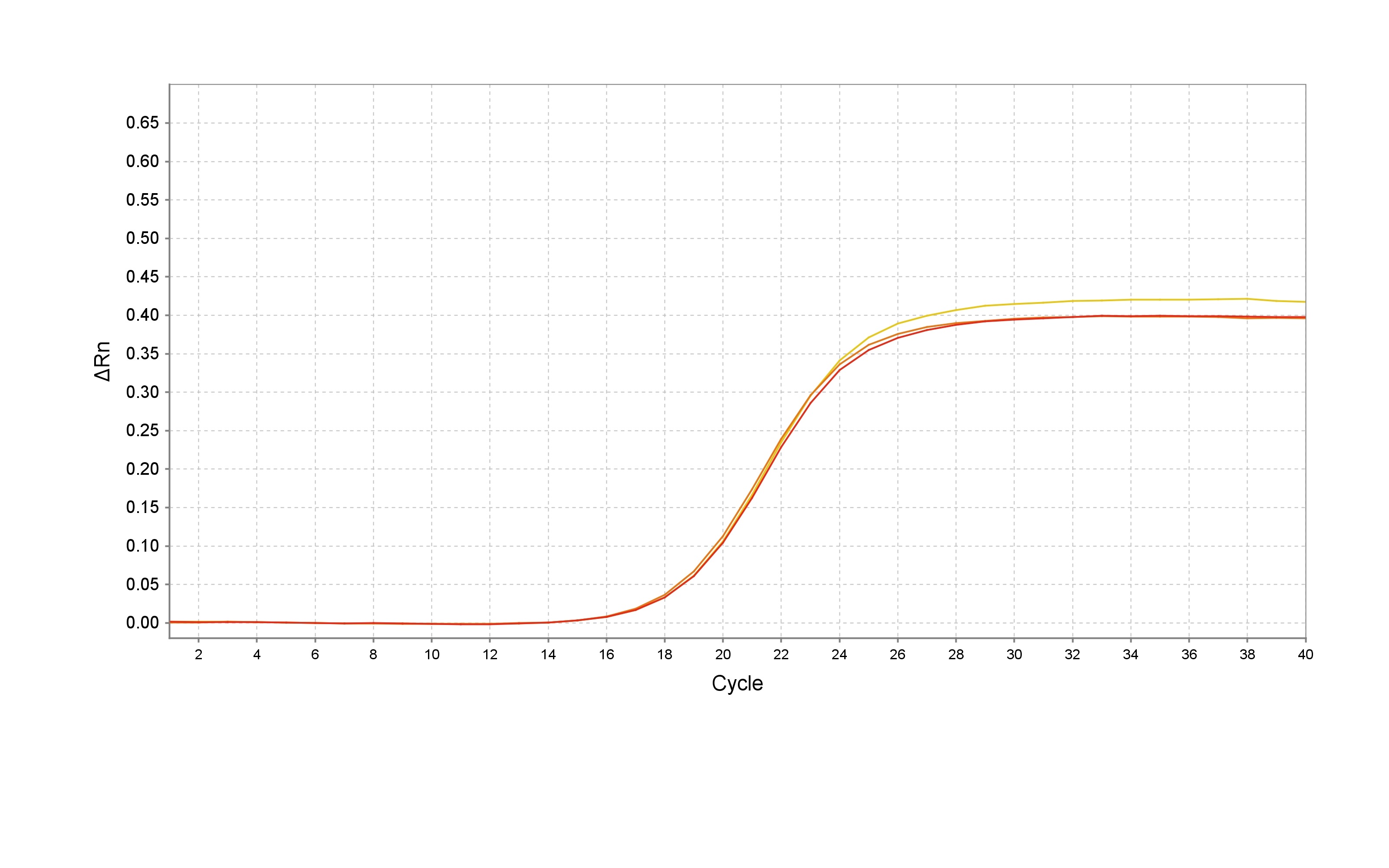

Supplement: Supplementary file 1 [file Data_Sheet_1.ZIP › Raw data1/RT-qPCR/└⌐╘÷╟·╧▀/GAPDH (B1 ó┘).jpg]

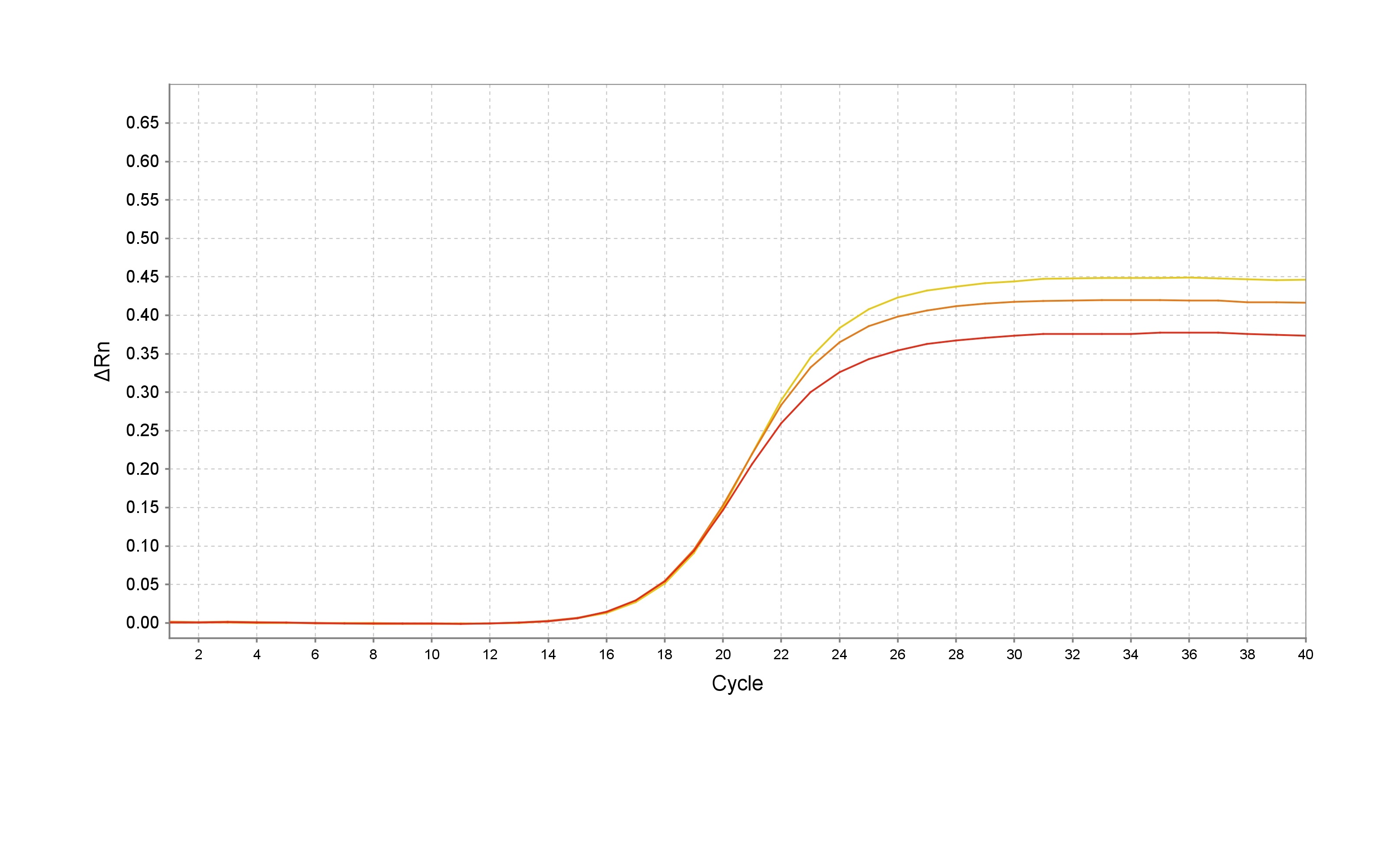

Supplement: Supplementary file 1 [file Data_Sheet_1.ZIP › Raw data1/RT-qPCR/└⌐╘÷╟·╧▀/GAPDH (B1 ó┌).jpg]

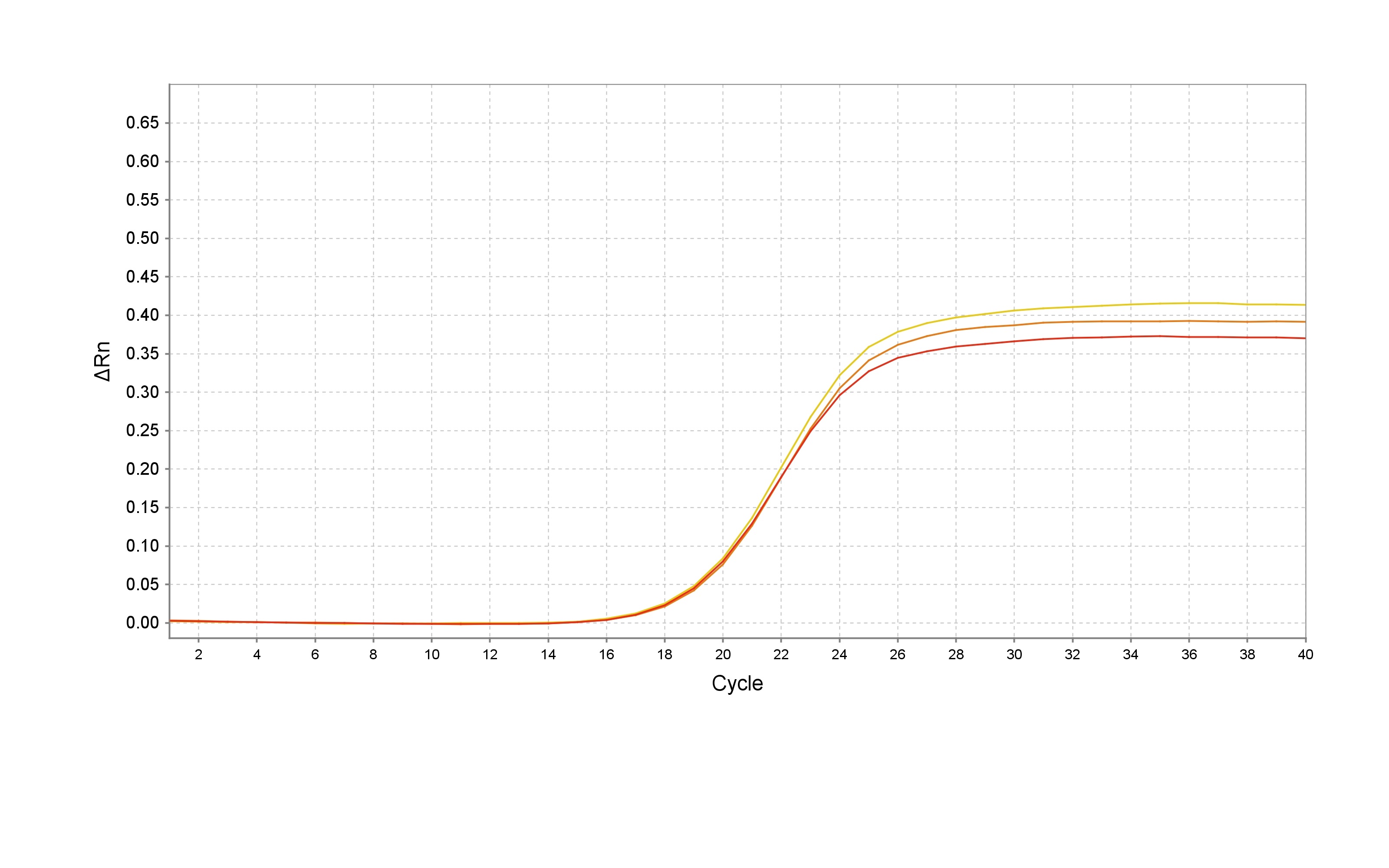

Supplement: Supplementary file 1 [file Data_Sheet_1.ZIP › Raw data1/RT-qPCR/└⌐╘÷╟·╧▀/GAPDH (B1 ó█).jpg]

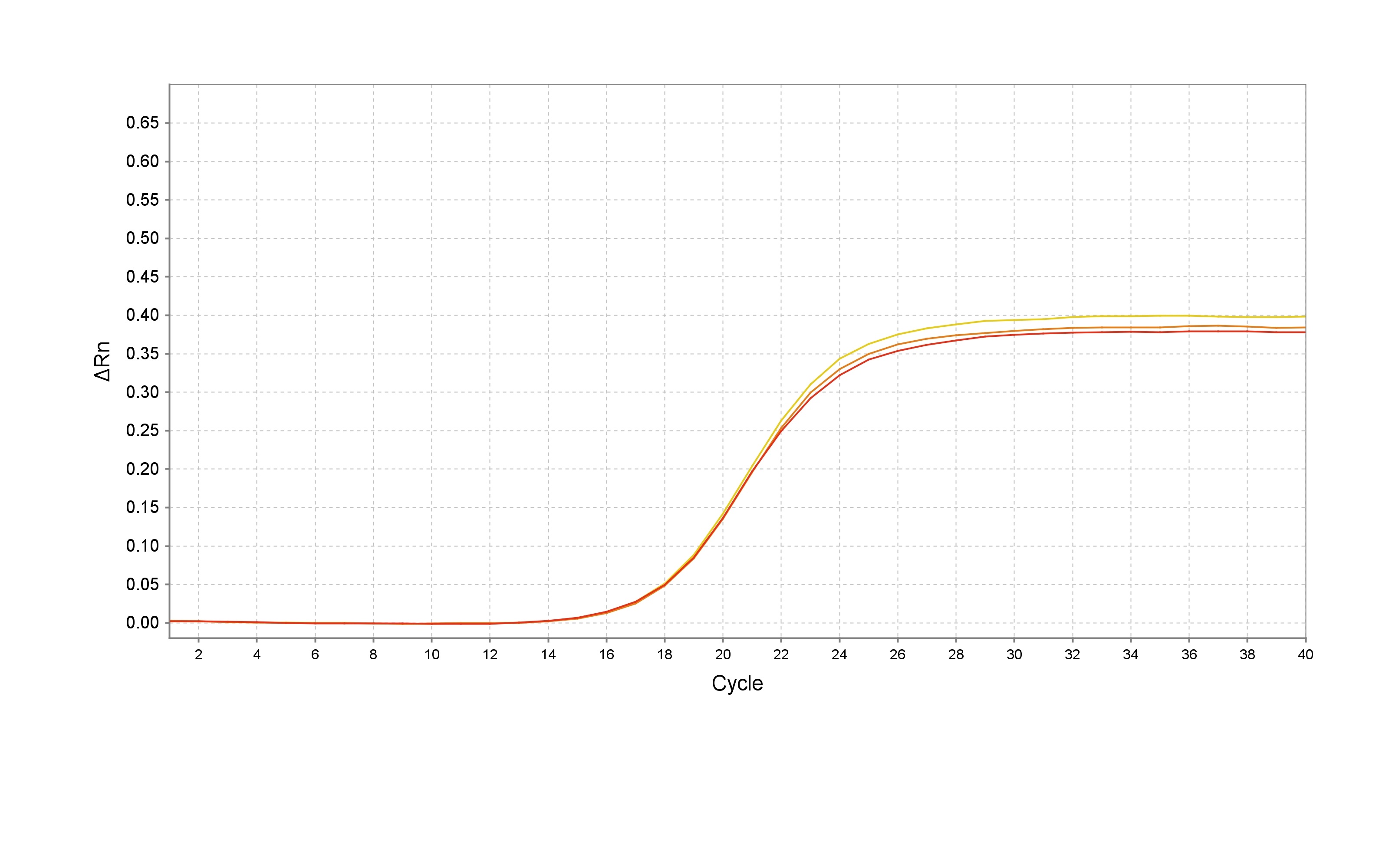

Supplement: Supplementary file 1 [file Data_Sheet_1.ZIP › Raw data1/RT-qPCR/└⌐╘÷╟·╧▀/GAPDH (C1 ó┘).jpg]

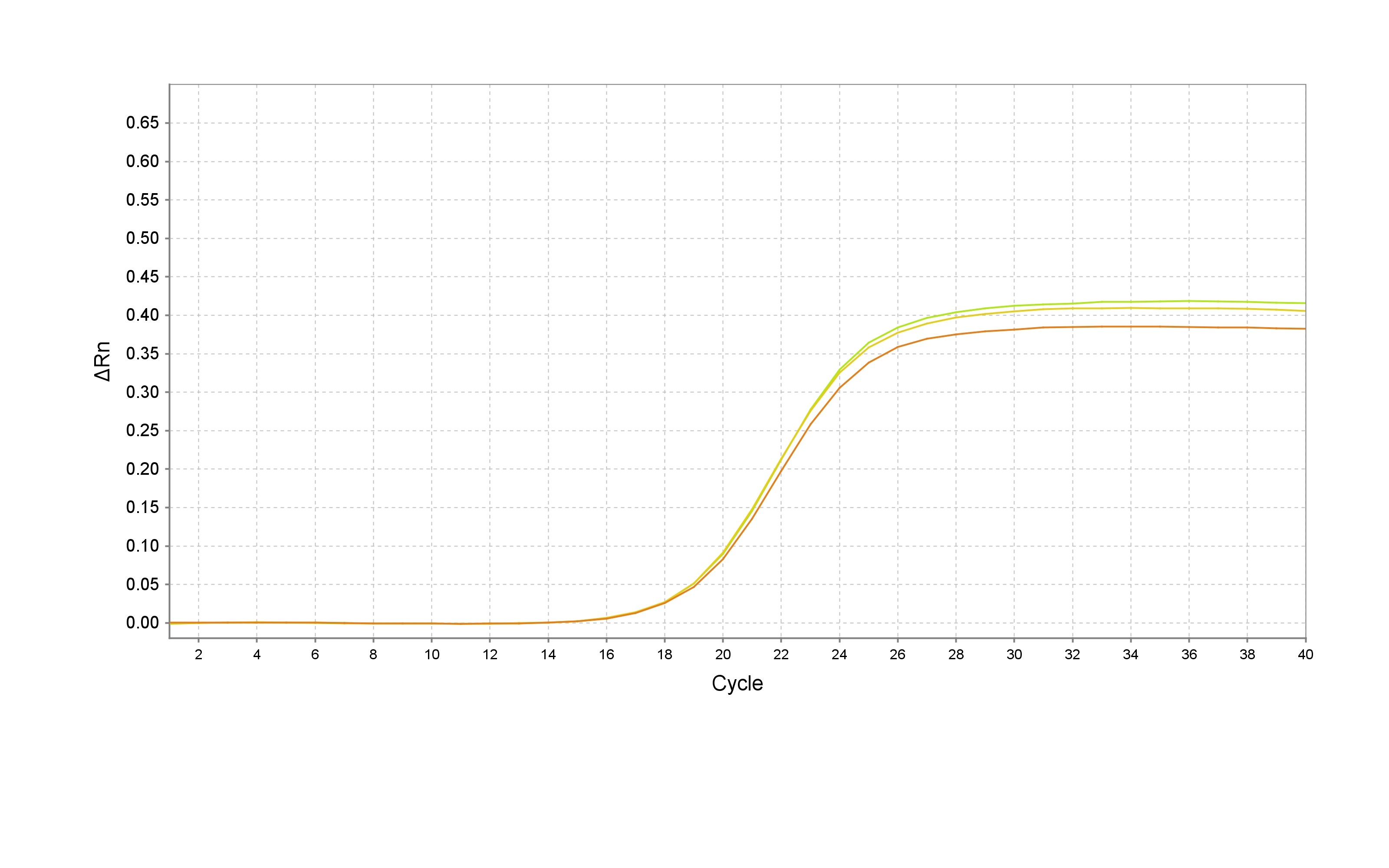

Supplement: Supplementary file 1 [file Data_Sheet_1.ZIP › Raw data1/RT-qPCR/└⌐╘÷╟·╧▀/GAPDH (C1 ó┌).jpg]

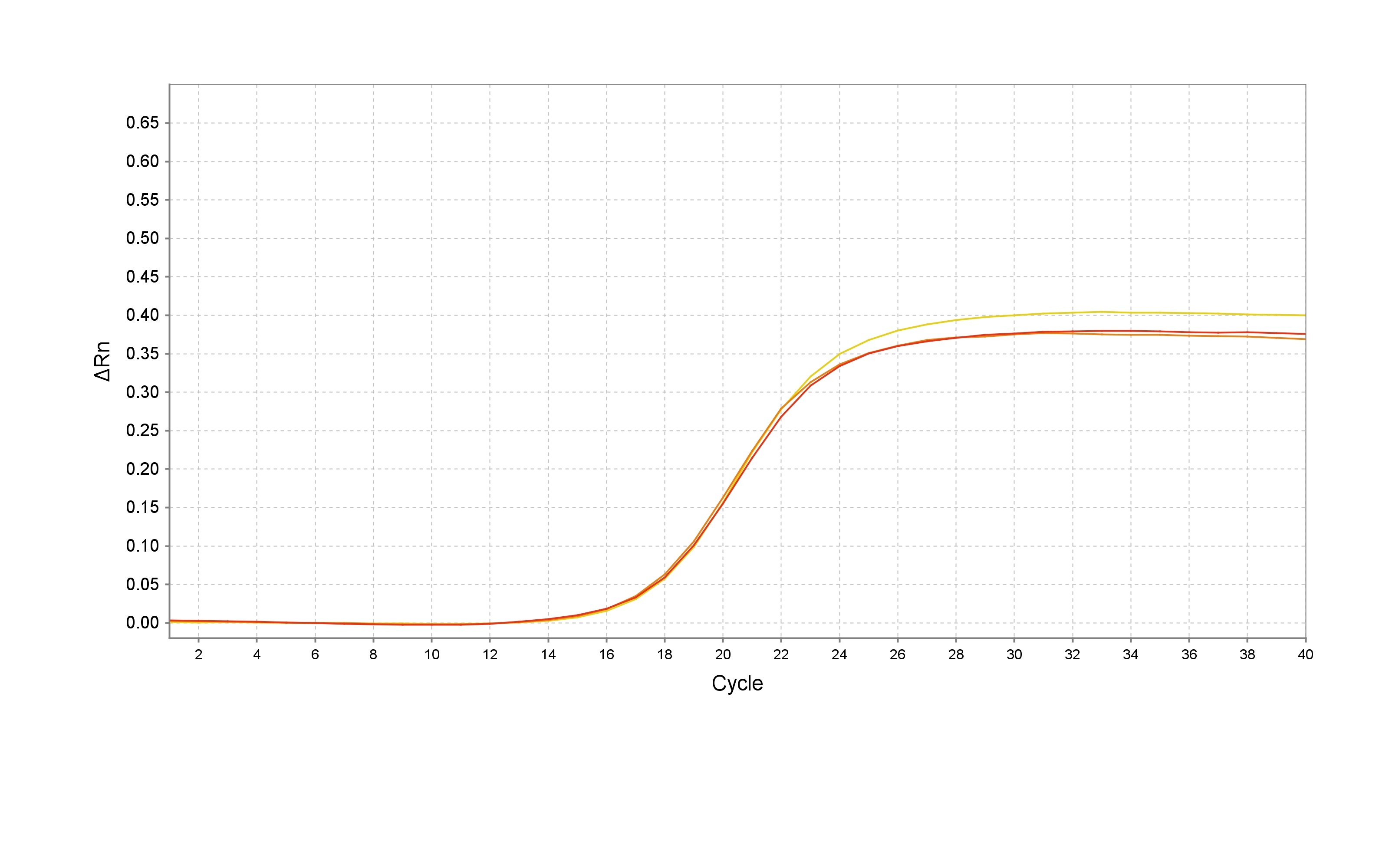

Supplement: Supplementary file 1 [file Data_Sheet_1.ZIP › Raw data1/RT-qPCR/└⌐╘÷╟·╧▀/GAPDH (C1 ó█).jpg]

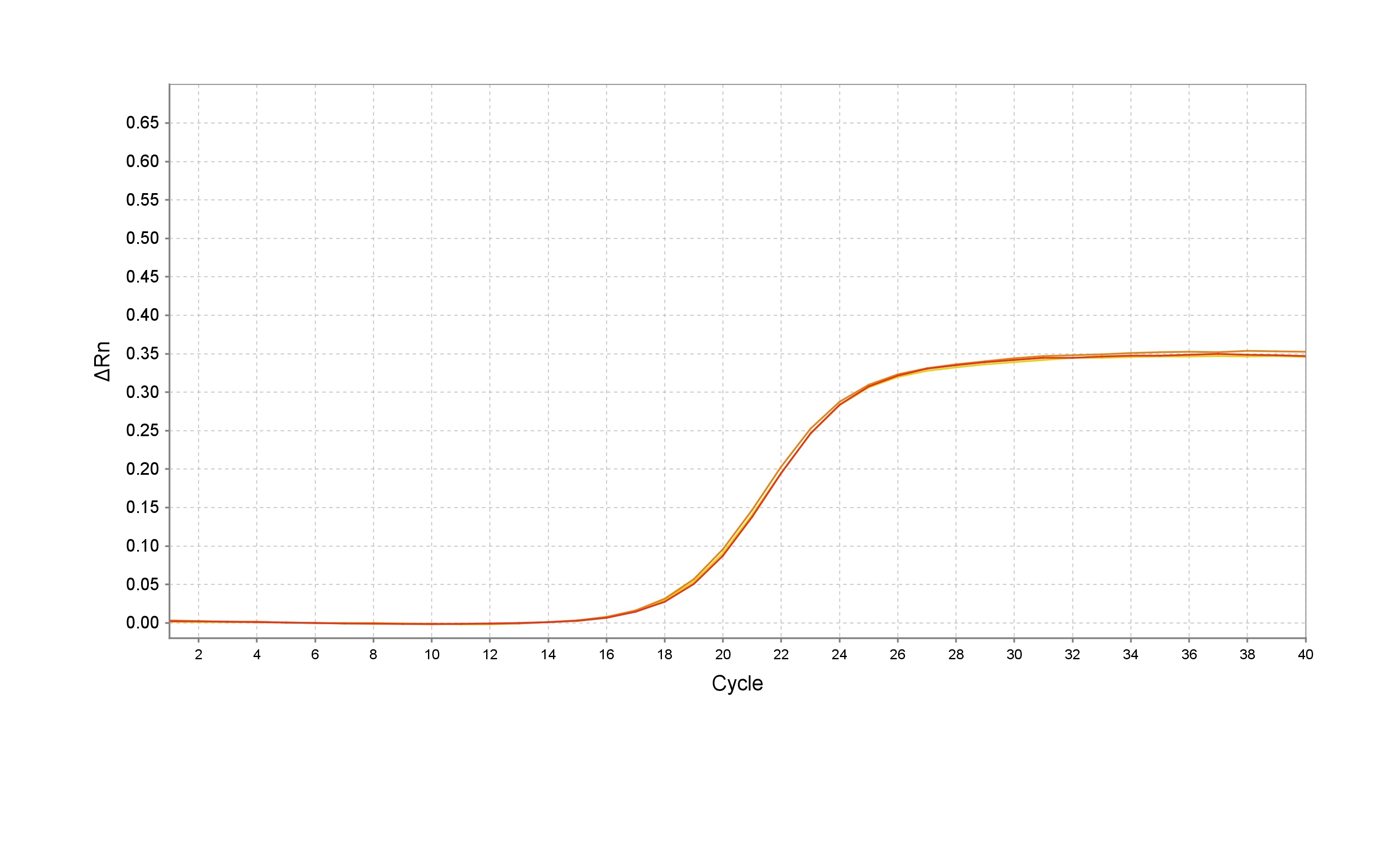

Supplement: Supplementary file 1 [file Data_Sheet_1.ZIP › Raw data1/RT-qPCR/└⌐╘÷╟·╧▀/GAPDH (D1 ó┘).jpg]

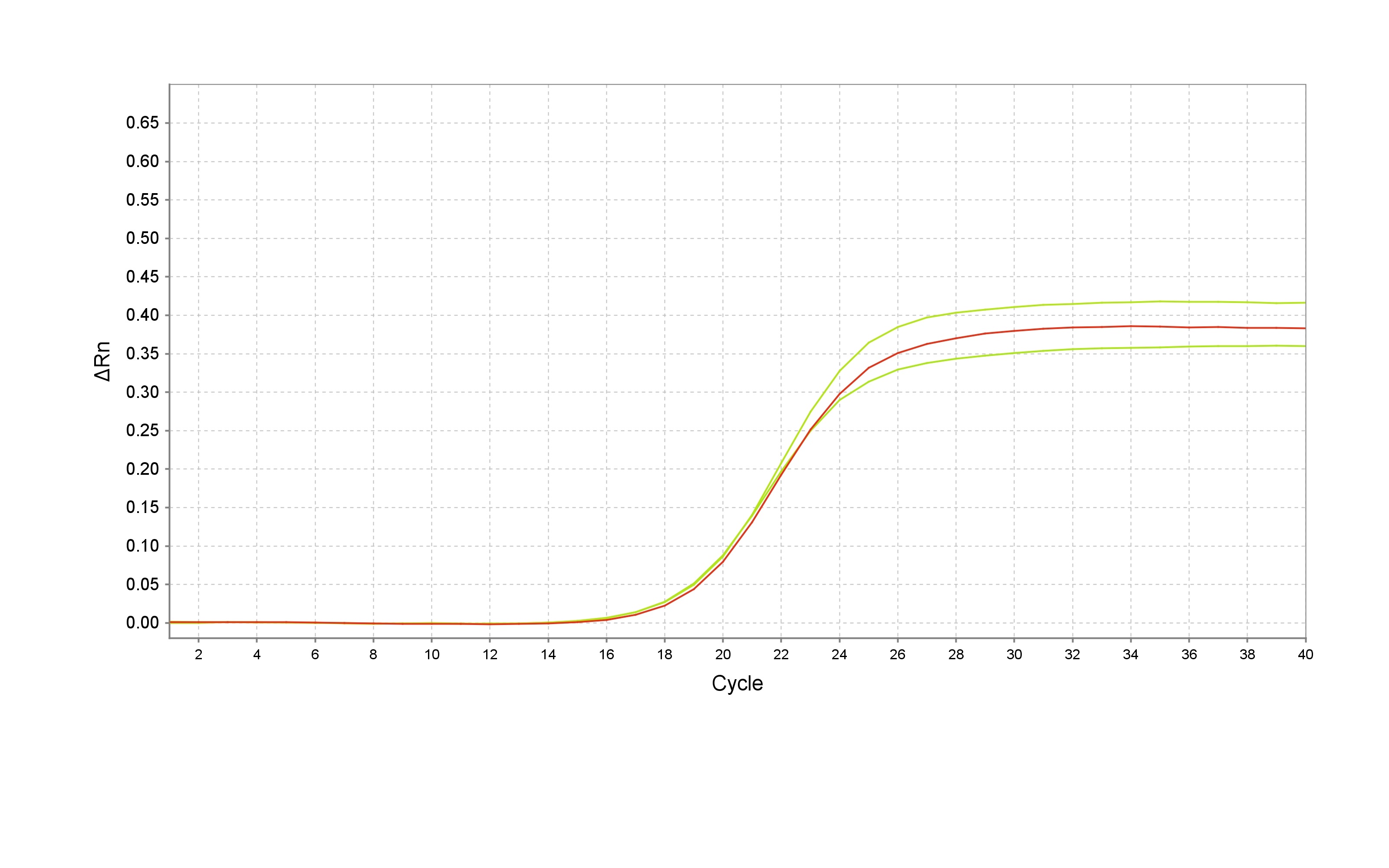

Supplement: Supplementary file 1 [file Data_Sheet_1.ZIP › Raw data1/RT-qPCR/└⌐╘÷╟·╧▀/GAPDH (D1 ó┌).jpg]

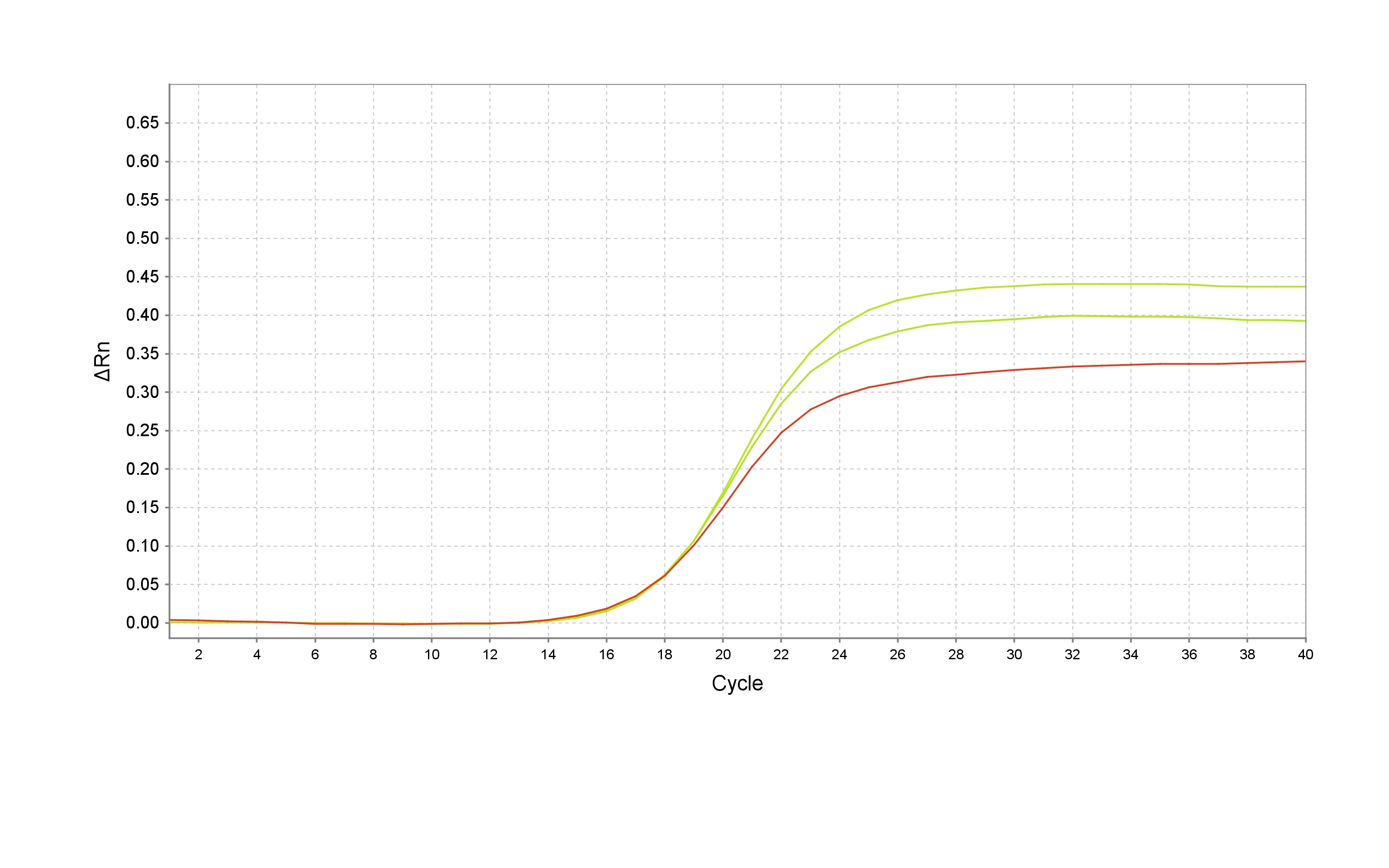

Supplement: Supplementary file 1 [file Data_Sheet_1.ZIP › Raw data1/RT-qPCR/└⌐╘÷╟·╧▀/GAPDH (D1 ó█).jpg]

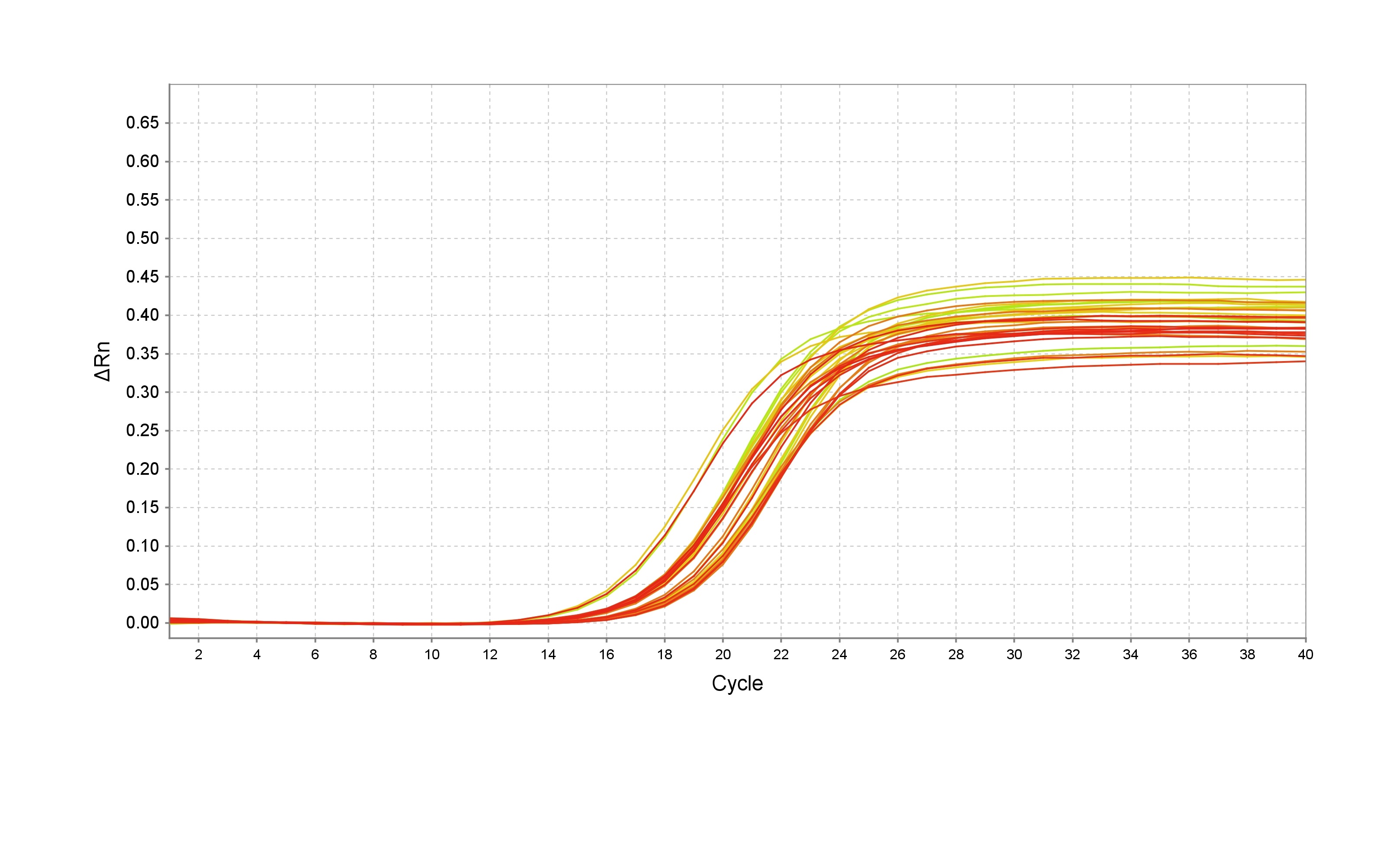

Supplement: Supplementary file 1 [file Data_Sheet_1.ZIP › Raw data1/RT-qPCR/└⌐╘÷╟·╧▀/GAPDH.jpg]

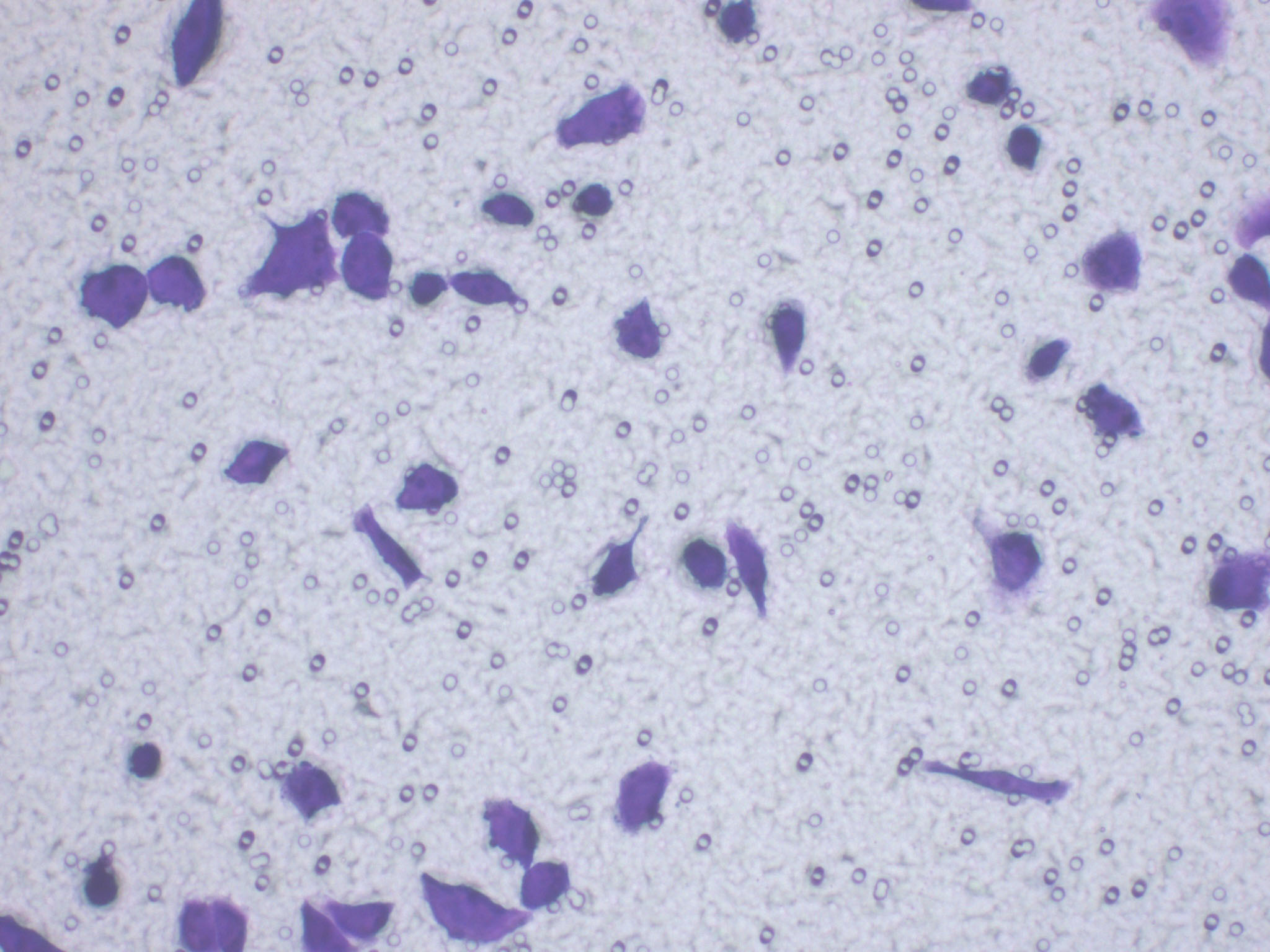

Supplement: Supplementary file 1 [file Data_Sheet_1.ZIP › Raw data1/Transwell migration and invasion assay/control.jpg]

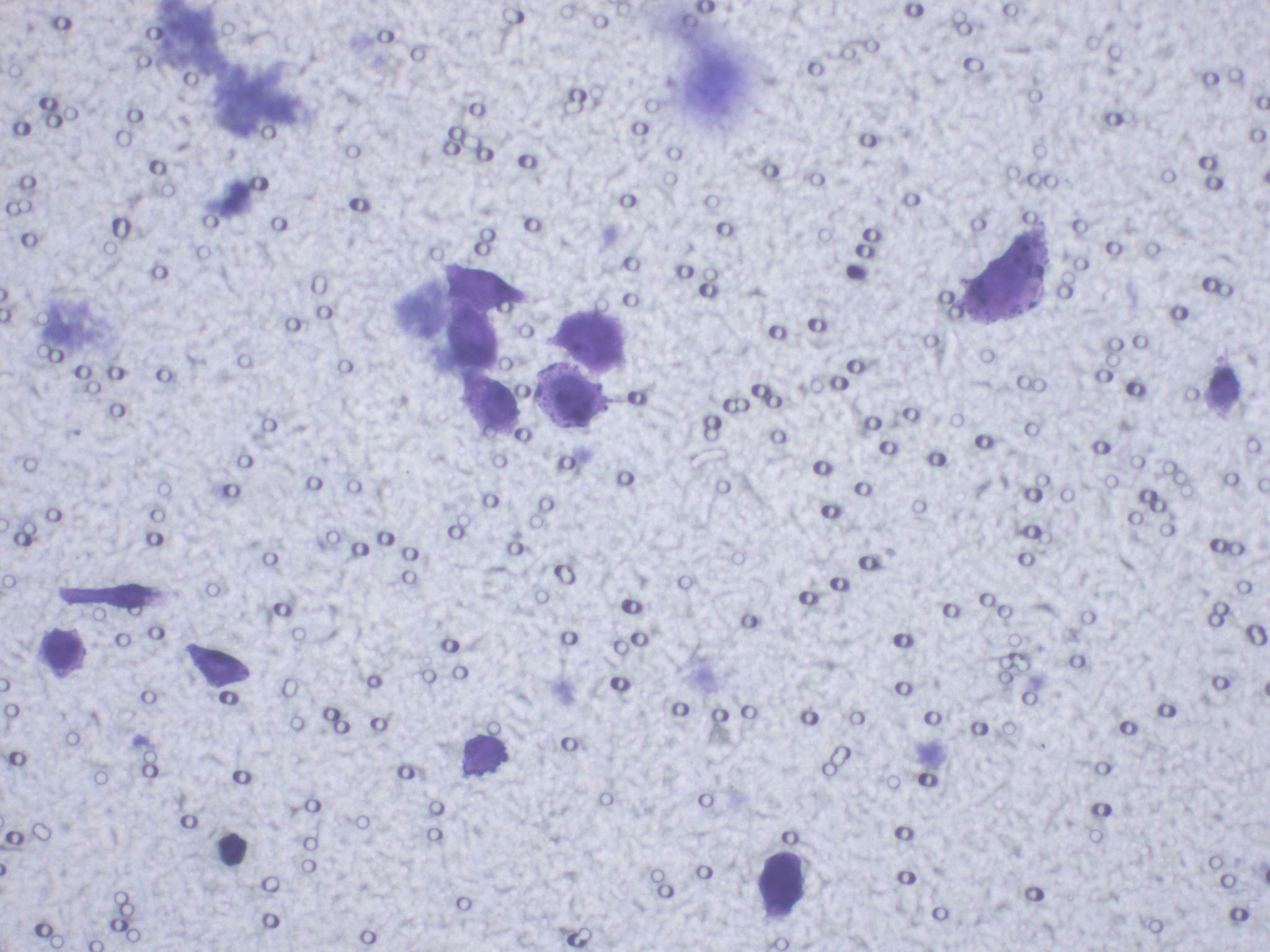

Supplement: Supplementary file 1 [file Data_Sheet_1.ZIP › Raw data1/Transwell migration and invasion assay/siCAPN2.jpg]

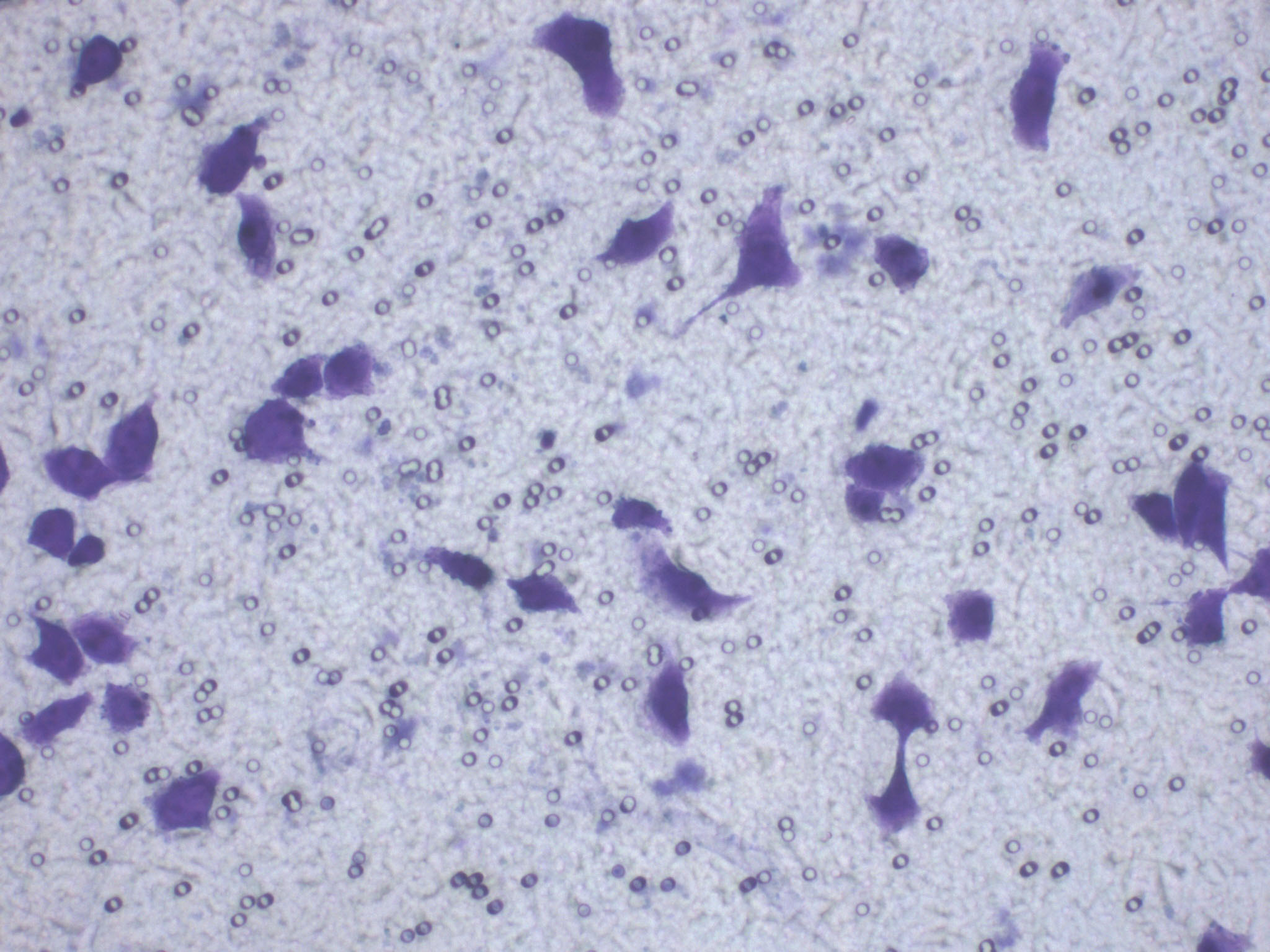

Supplement: Supplementary file 1 [file Data_Sheet_1.ZIP › Raw data1/Transwell migration and invasion assay/siNC.jpg]

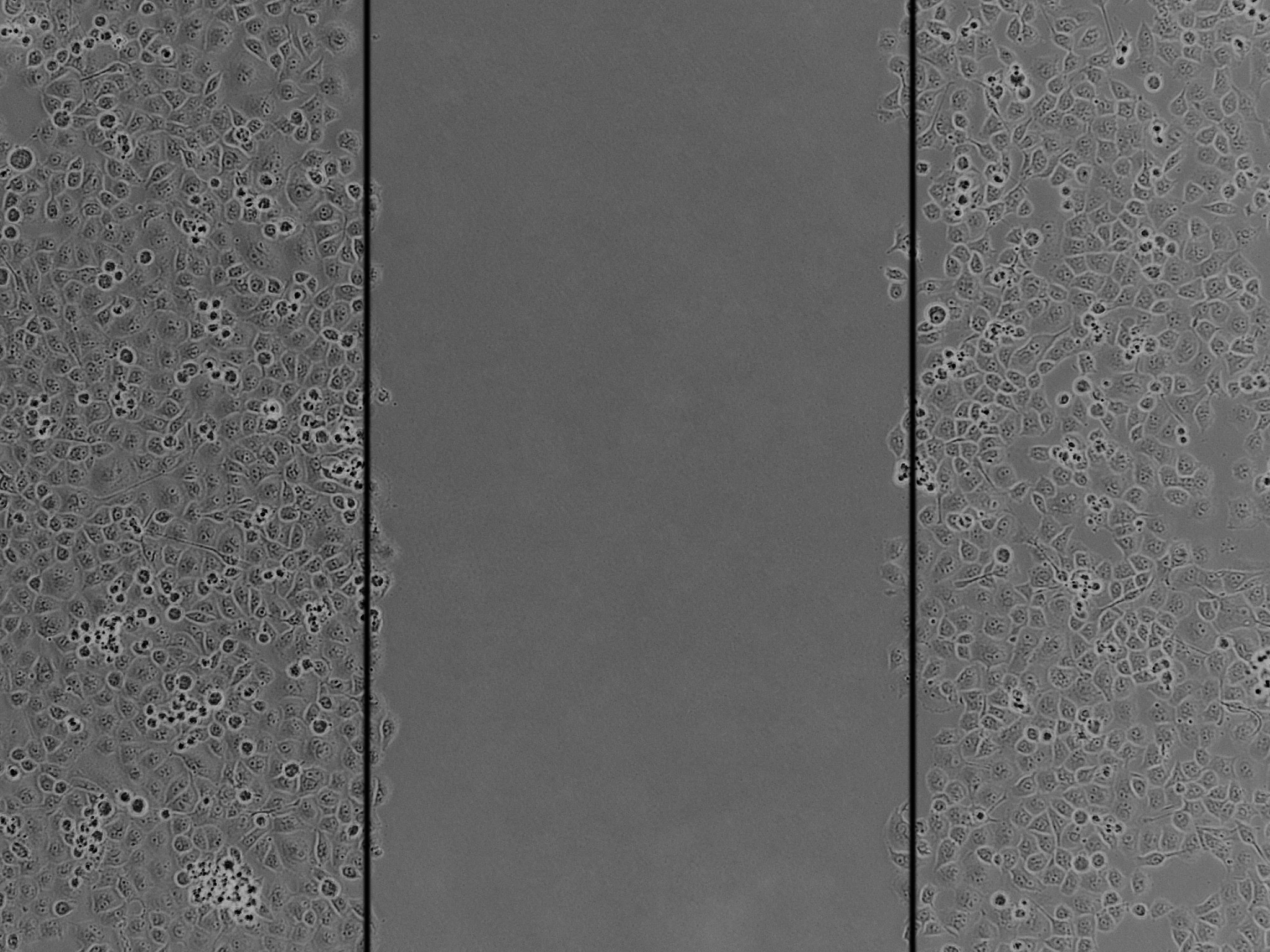

Supplement: Supplementary file 1 [file Data_Sheet_1.ZIP › Raw data1/Wound scratch assay/control-0h.jpg]

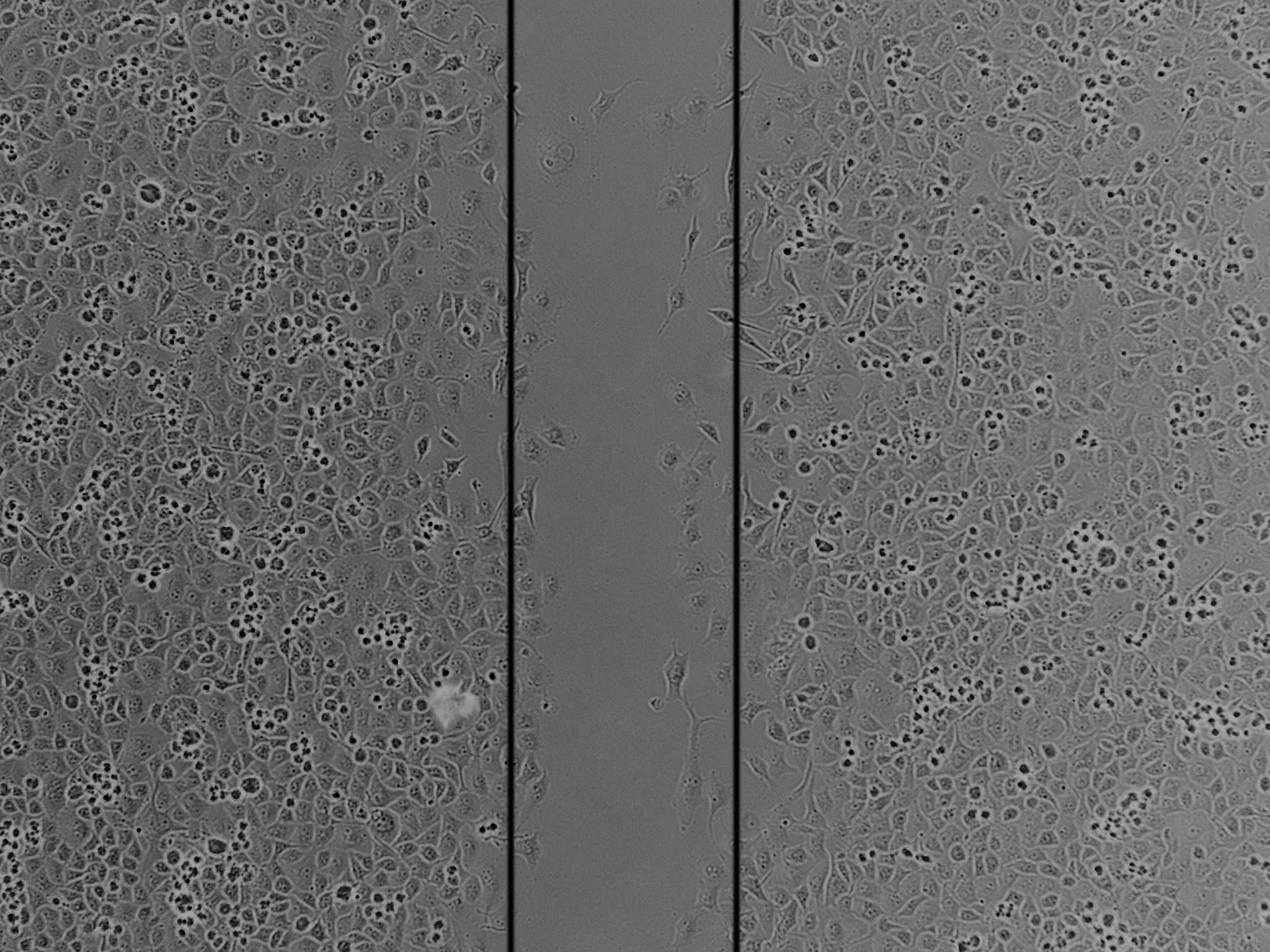

Supplement: Supplementary file 1 [file Data_Sheet_1.ZIP › Raw data1/Wound scratch assay/control24h.jpg]

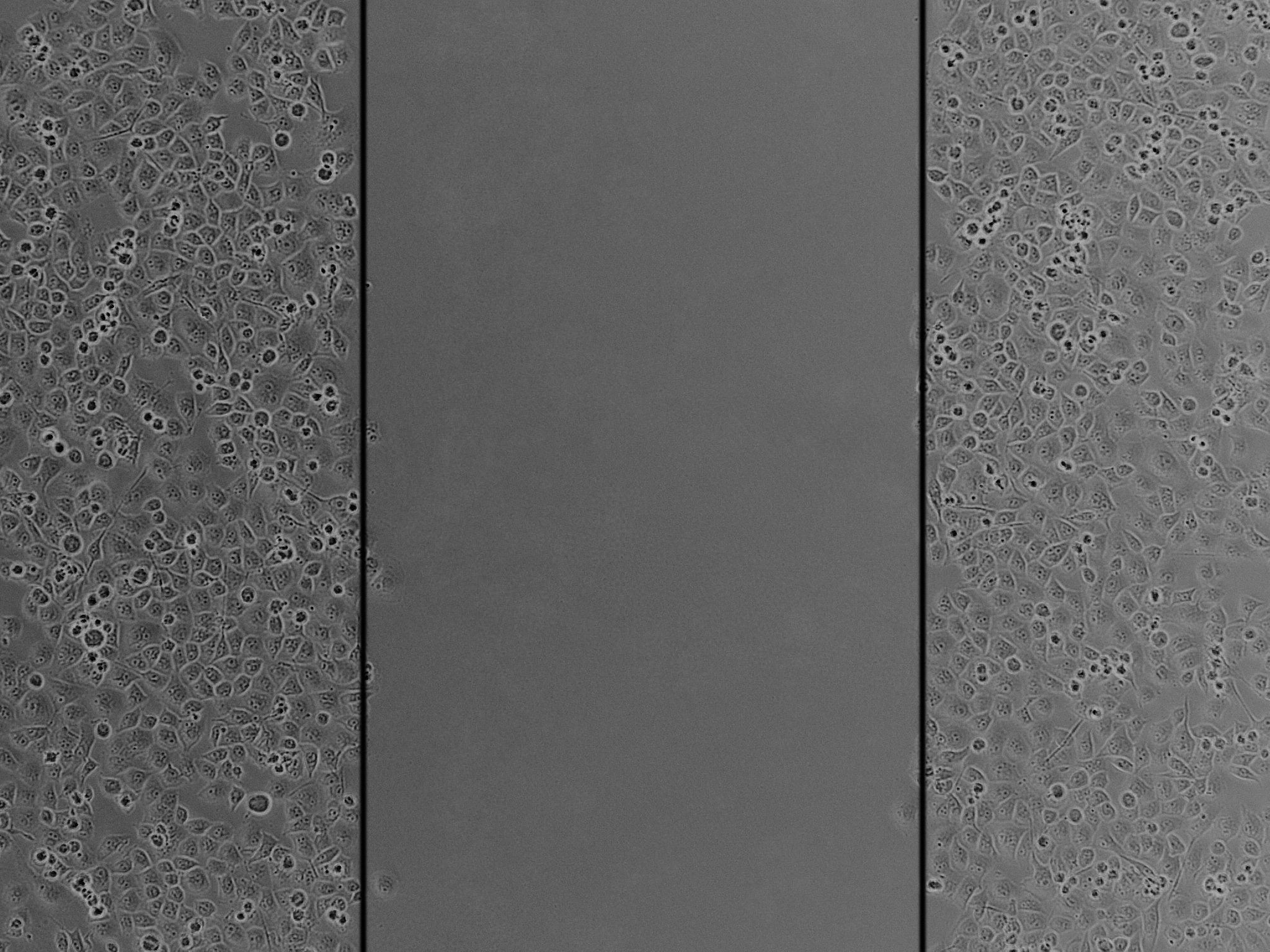

Supplement: Supplementary file 1 [file Data_Sheet_1.ZIP › Raw data1/Wound scratch assay/siCAPN2-0h.jpg]

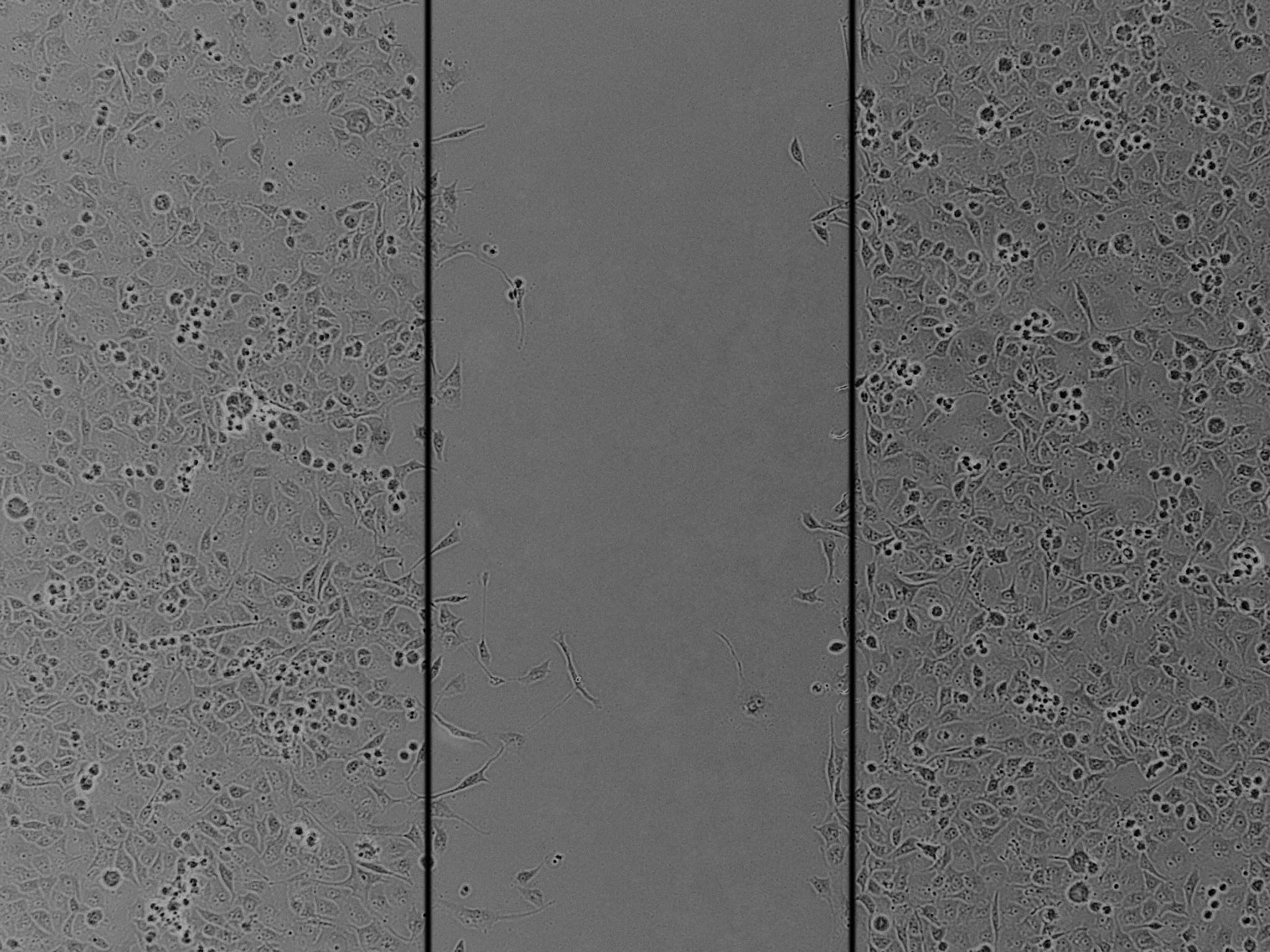

Supplement: Supplementary file 1 [file Data_Sheet_1.ZIP › Raw data1/Wound scratch assay/siCAPN2-24h.jpg]

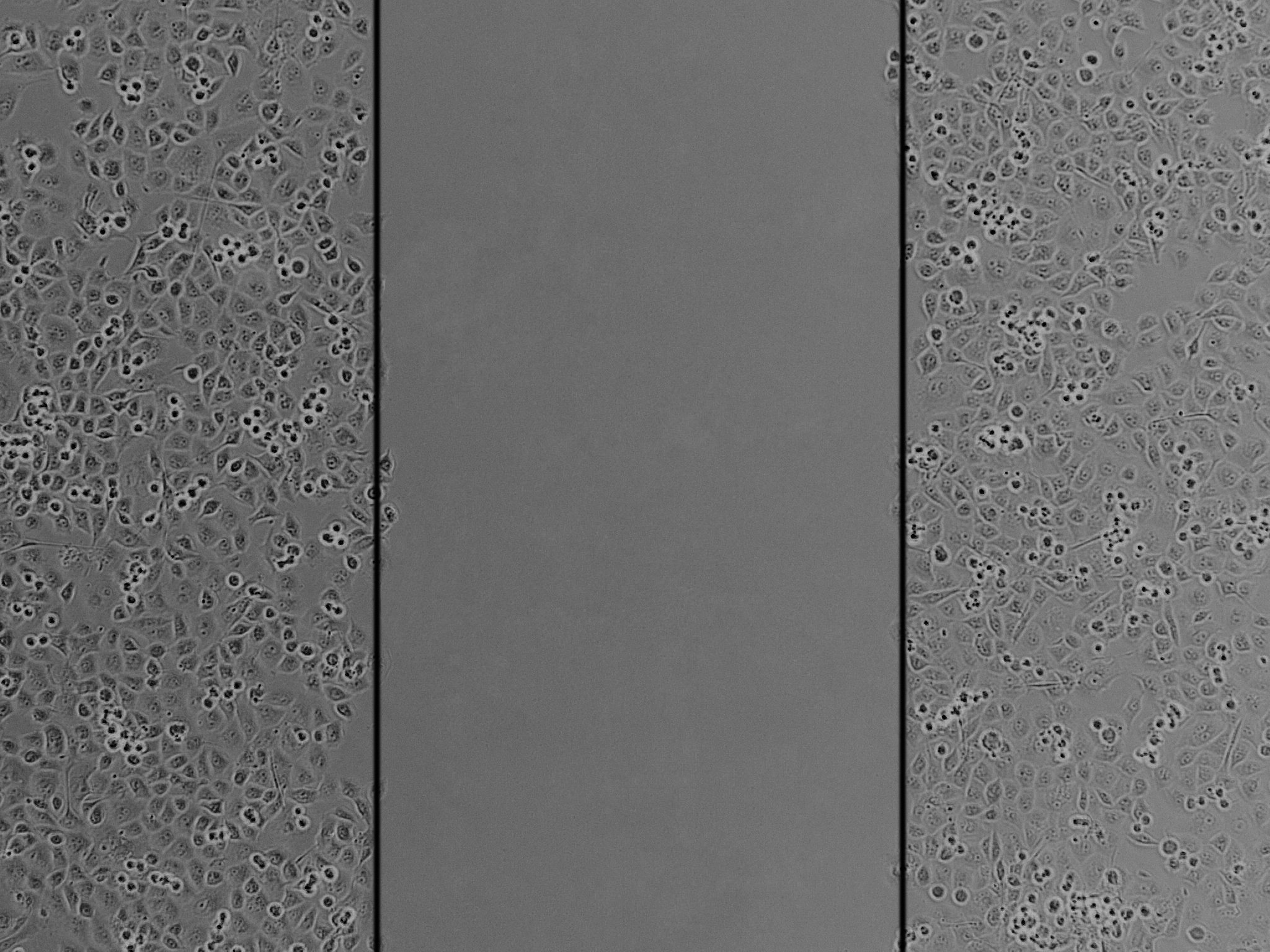

Supplement: Supplementary file 1 [file Data_Sheet_1.ZIP › Raw data1/Wound scratch assay/siNC-0h.jpg]

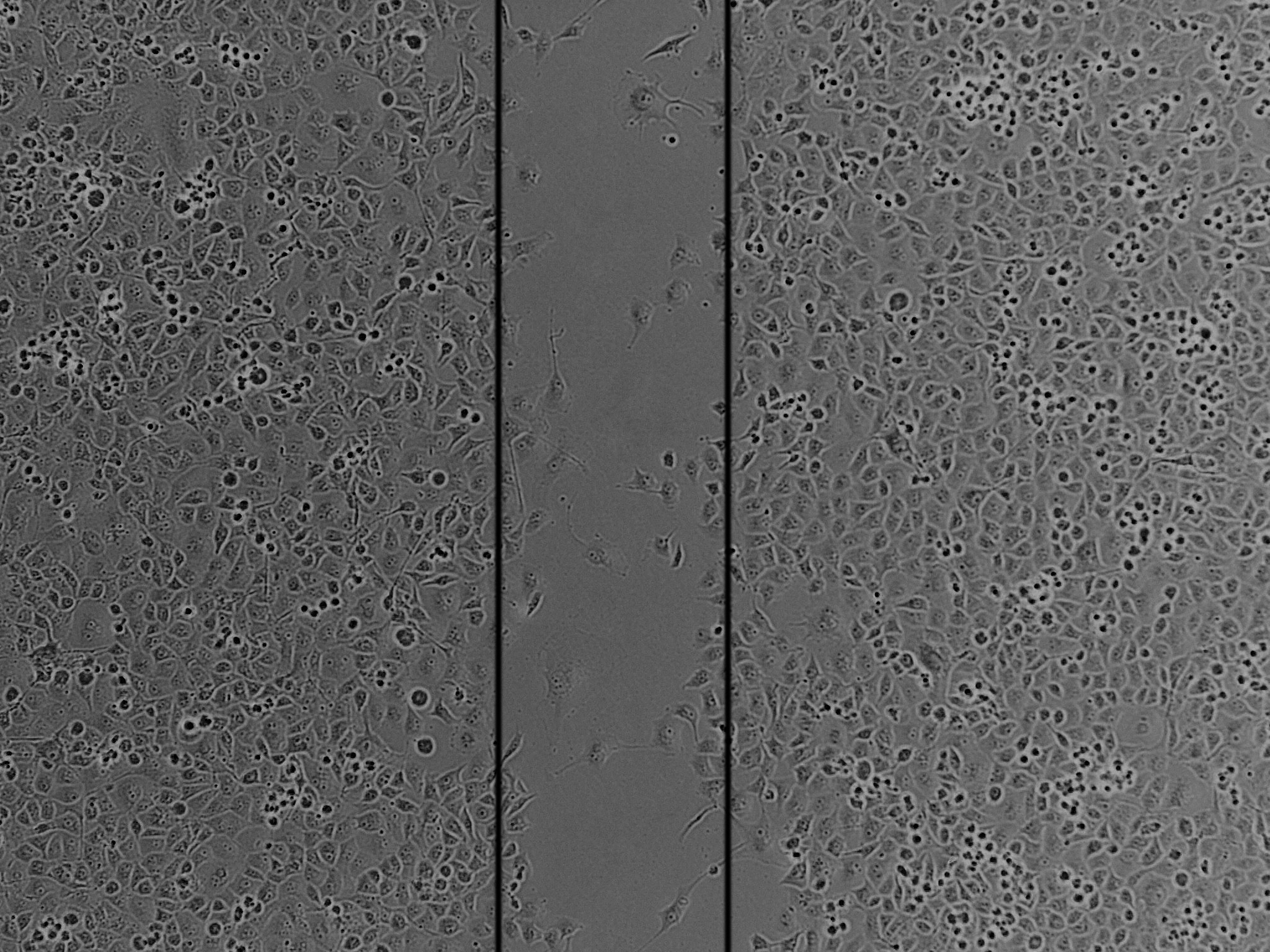

Supplement: Supplementary file 1 [file Data_Sheet_1.ZIP › Raw data1/Wound scratch assay/siNC-24H.jpg]

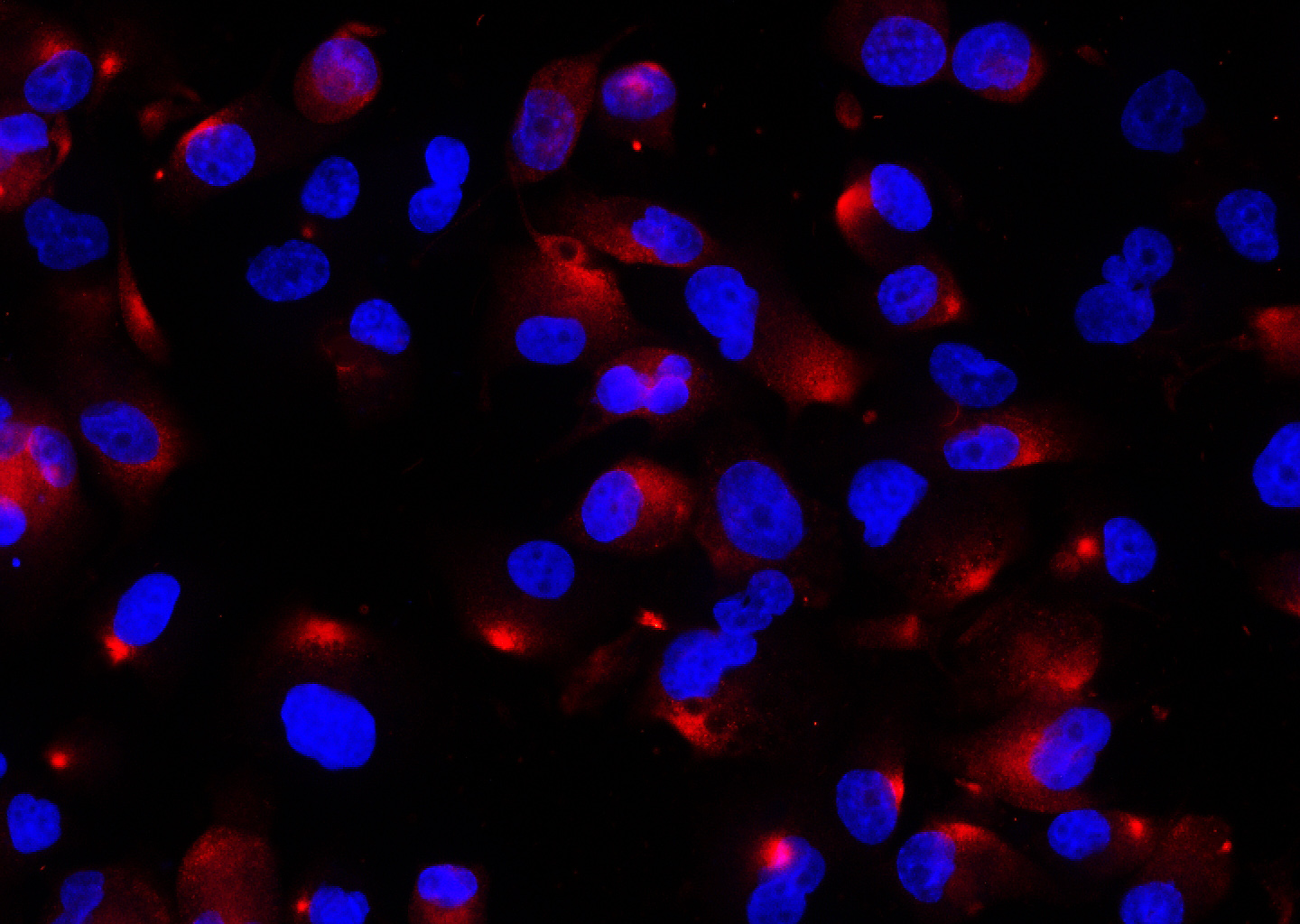

Supplement: Supplementary file 2 [file Data_Sheet_2.ZIP › Immunofluorescence staining/E-cad/A1-1 400-3+4.jpg]

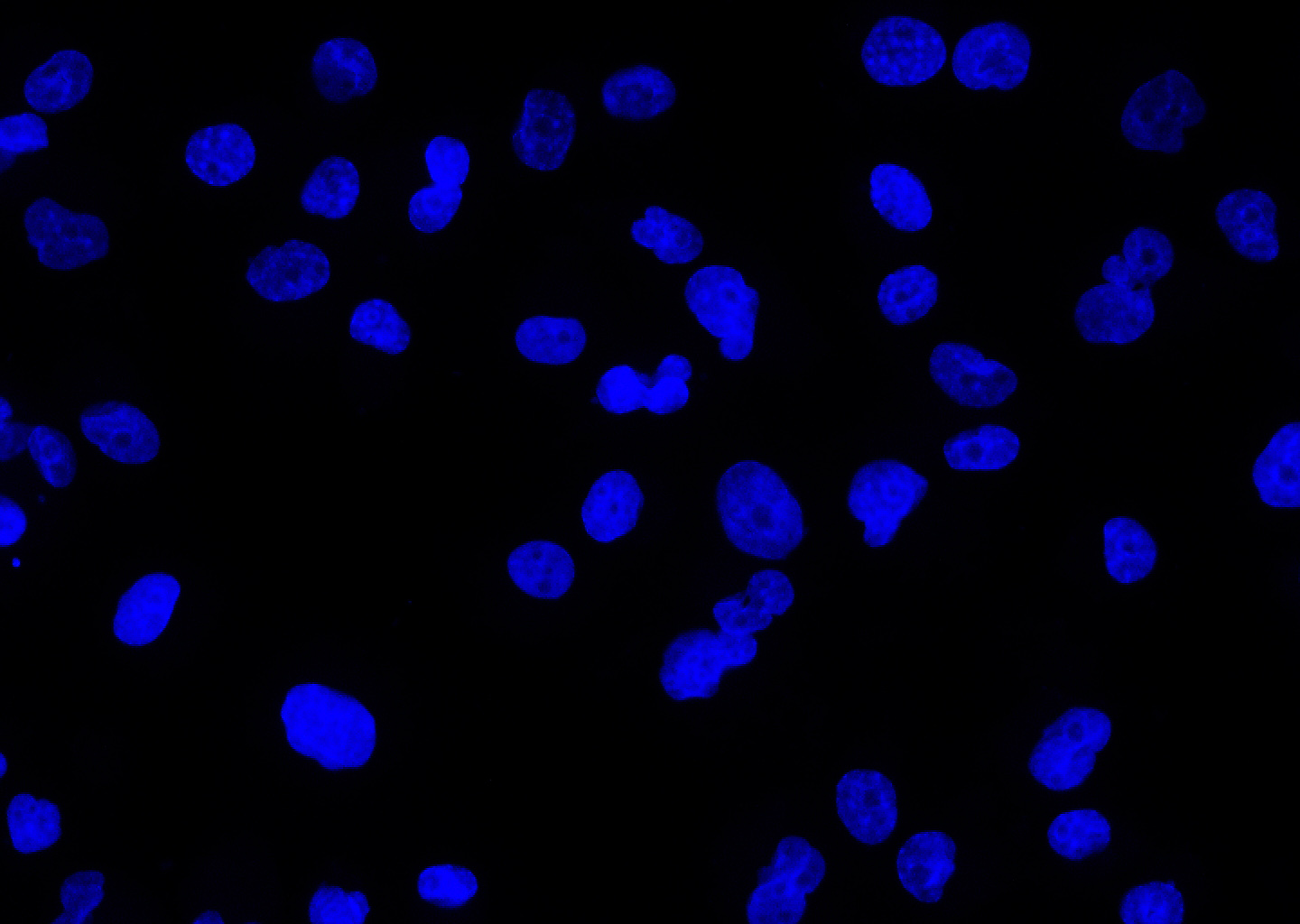

Supplement: Supplementary file 2 [file Data_Sheet_2.ZIP › Immunofluorescence staining/E-cad/A1-1 400-3.jpg]

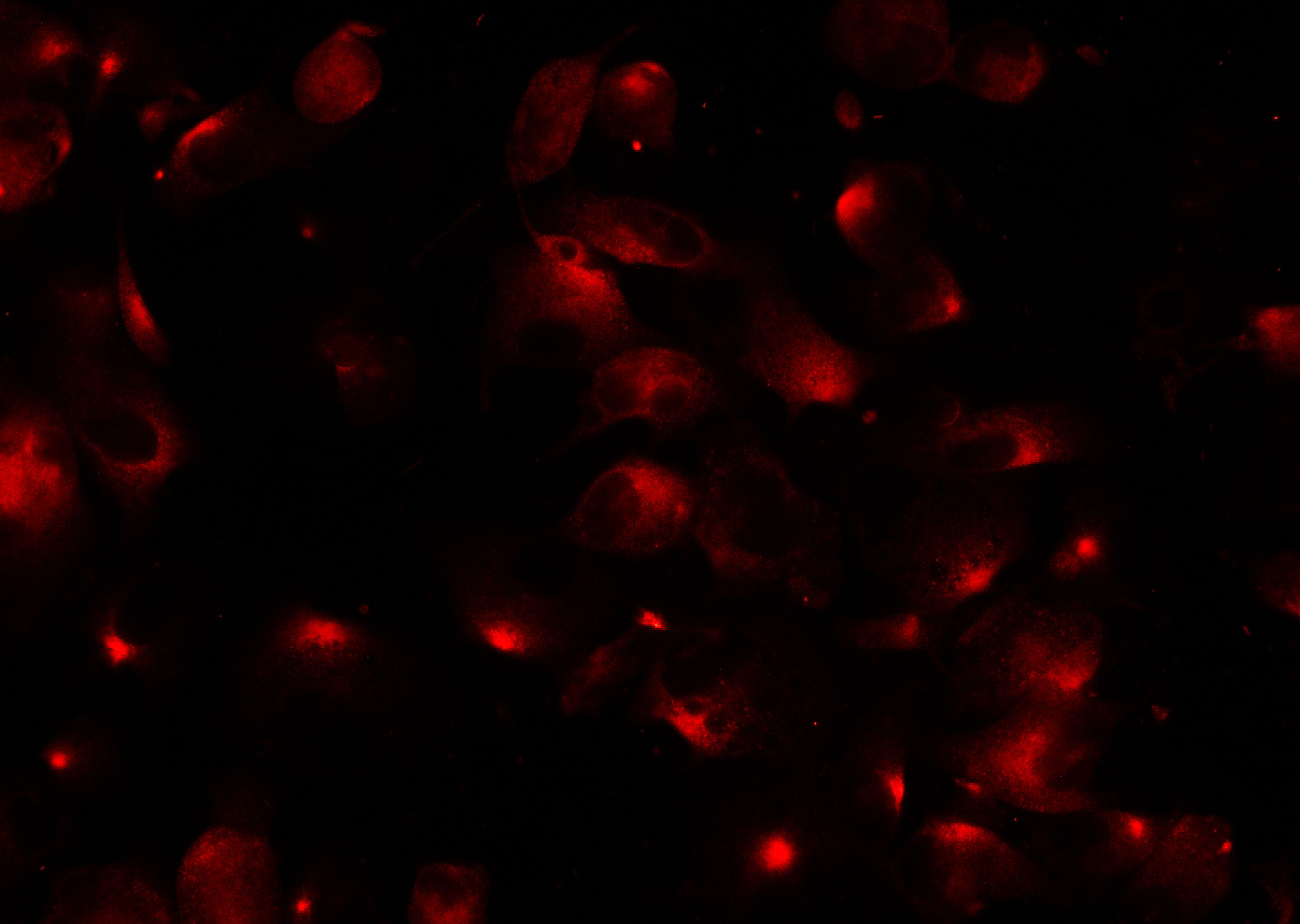

Supplement: Supplementary file 2 [file Data_Sheet_2.ZIP › Immunofluorescence staining/E-cad/A1-1 400-4.jpg]

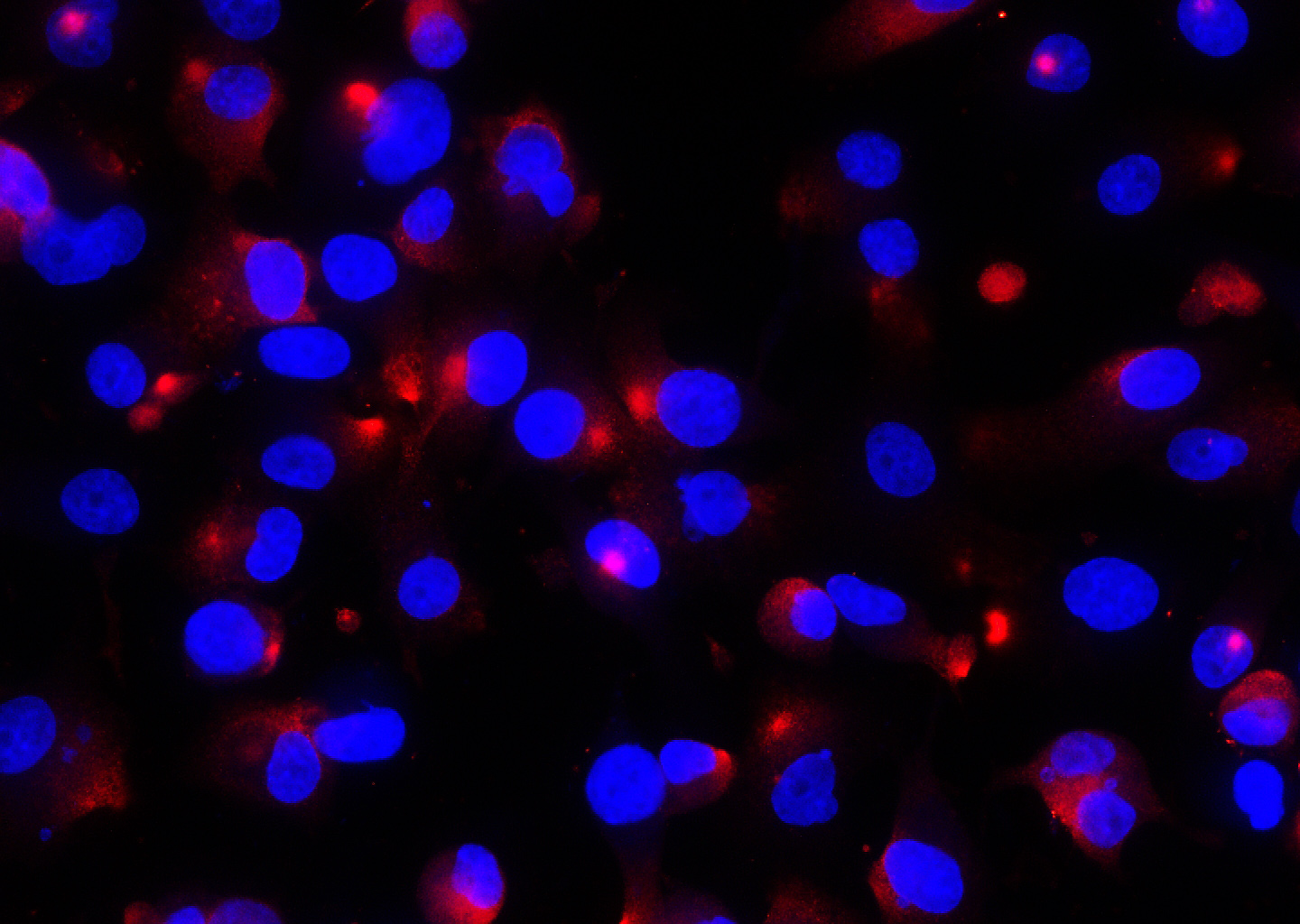

Supplement: Supplementary file 2 [file Data_Sheet_2.ZIP › Immunofluorescence staining/E-cad/B1-3 400-1+2.jpg]

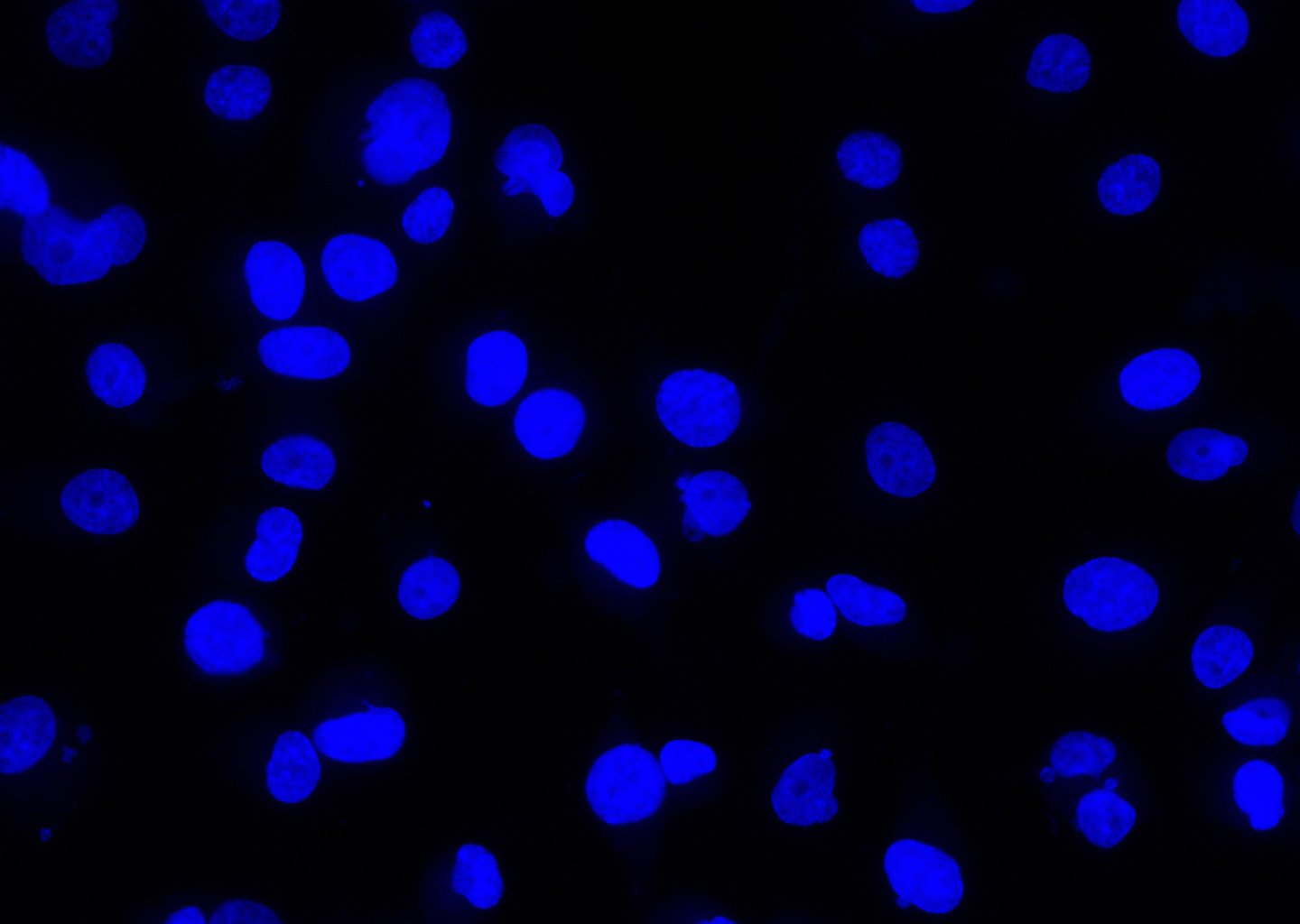

Supplement: Supplementary file 2 [file Data_Sheet_2.ZIP › Immunofluorescence staining/E-cad/B1-3 400-1.jpg]

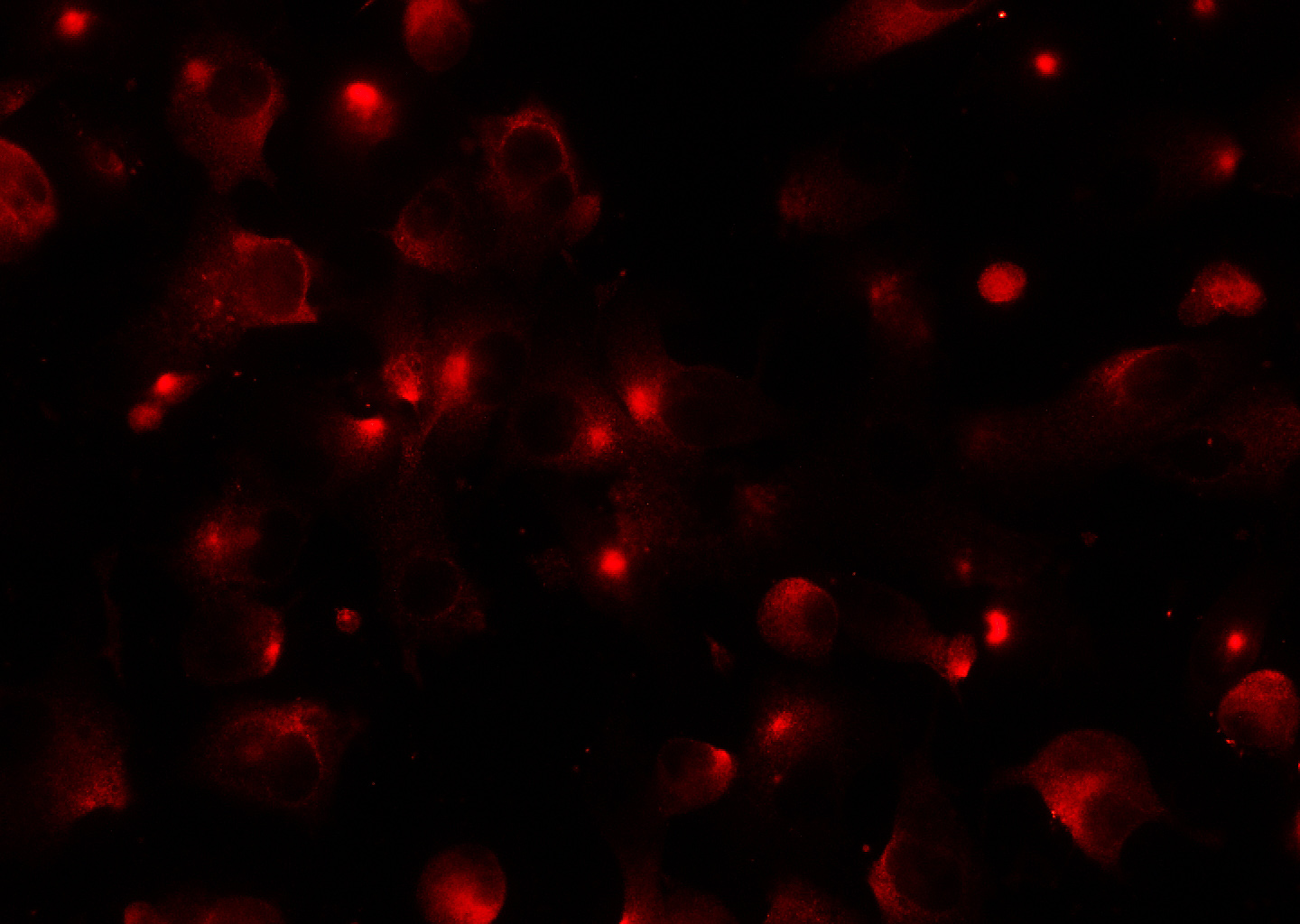

Supplement: Supplementary file 2 [file Data_Sheet_2.ZIP › Immunofluorescence staining/E-cad/B1-3 400-2.jpg]

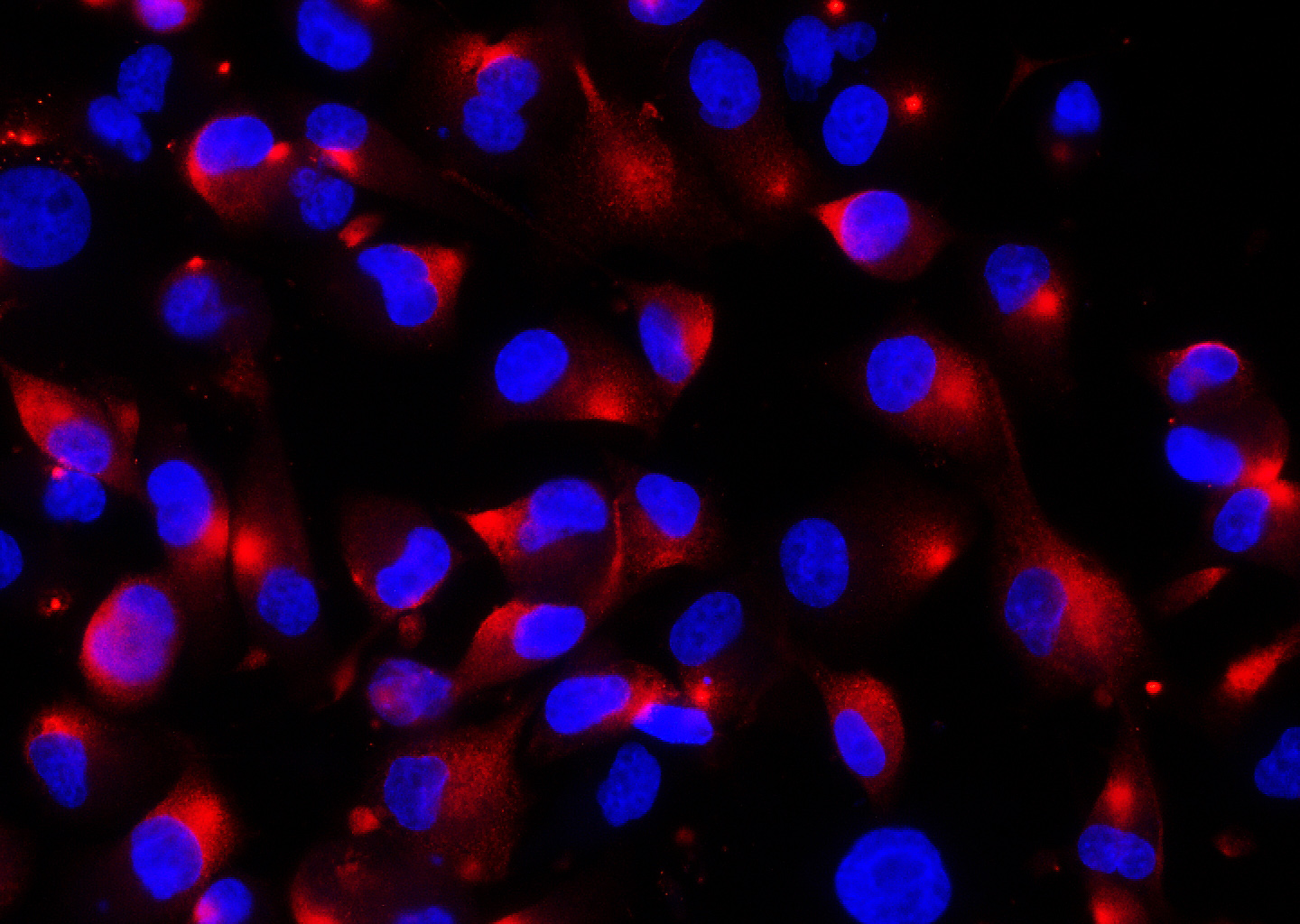

Supplement: Supplementary file 2 [file Data_Sheet_2.ZIP › Immunofluorescence staining/E-cad/C1-3 400-1+2.jpg]

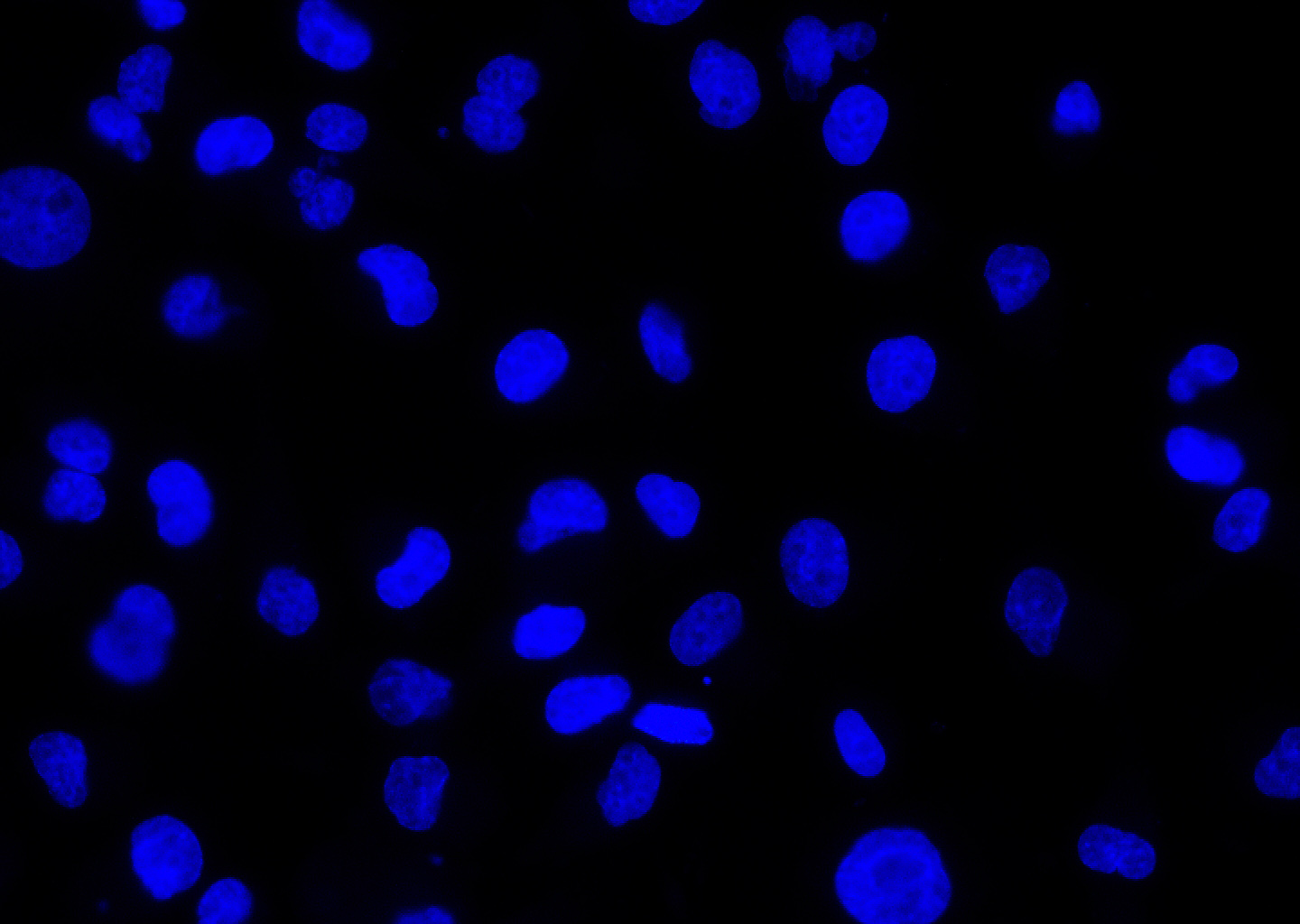

Supplement: Supplementary file 2 [file Data_Sheet_2.ZIP › Immunofluorescence staining/E-cad/C1-3 400-1.jpg]

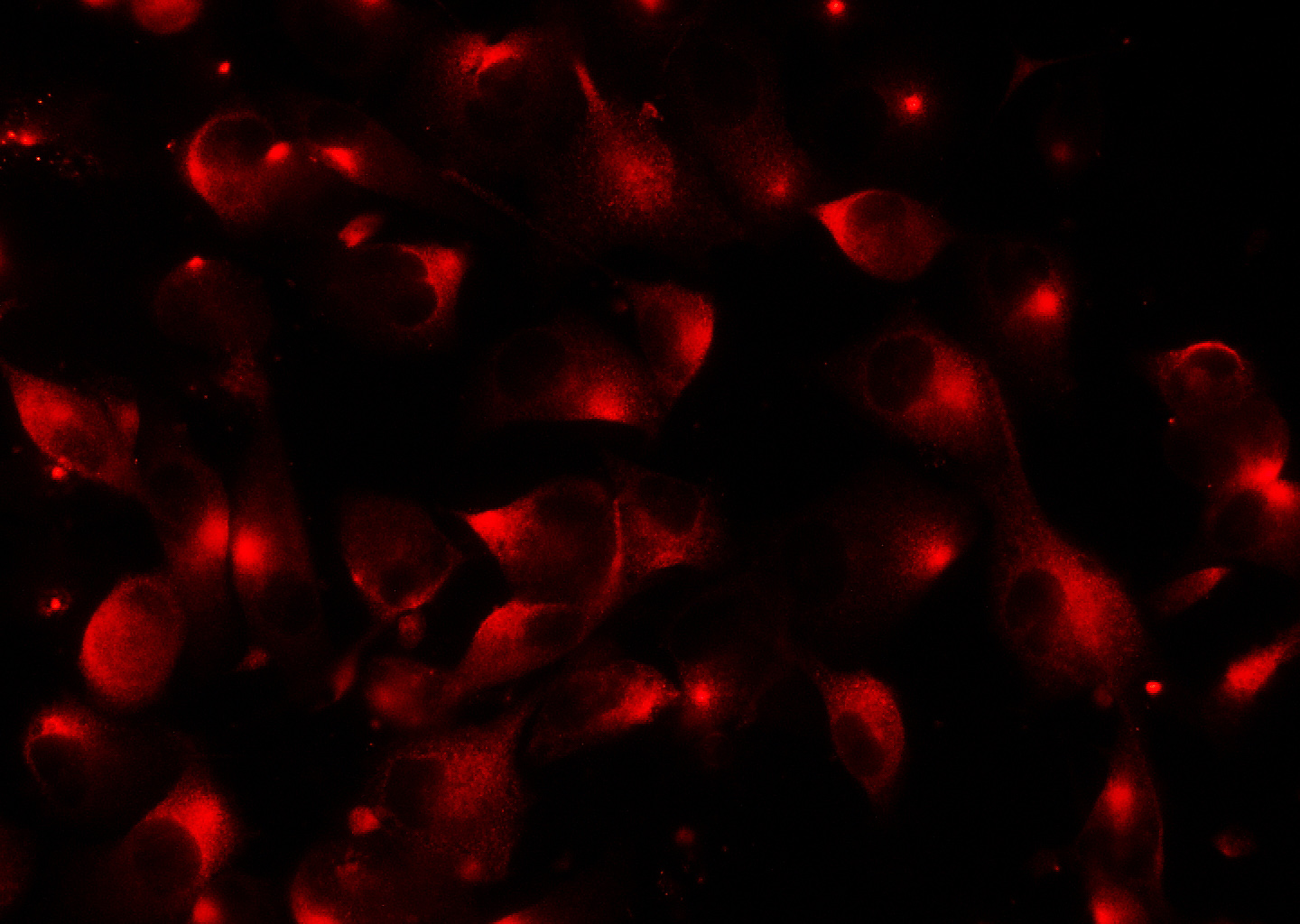

Supplement: Supplementary file 2 [file Data_Sheet_2.ZIP › Immunofluorescence staining/E-cad/C1-3 400-2.jpg]

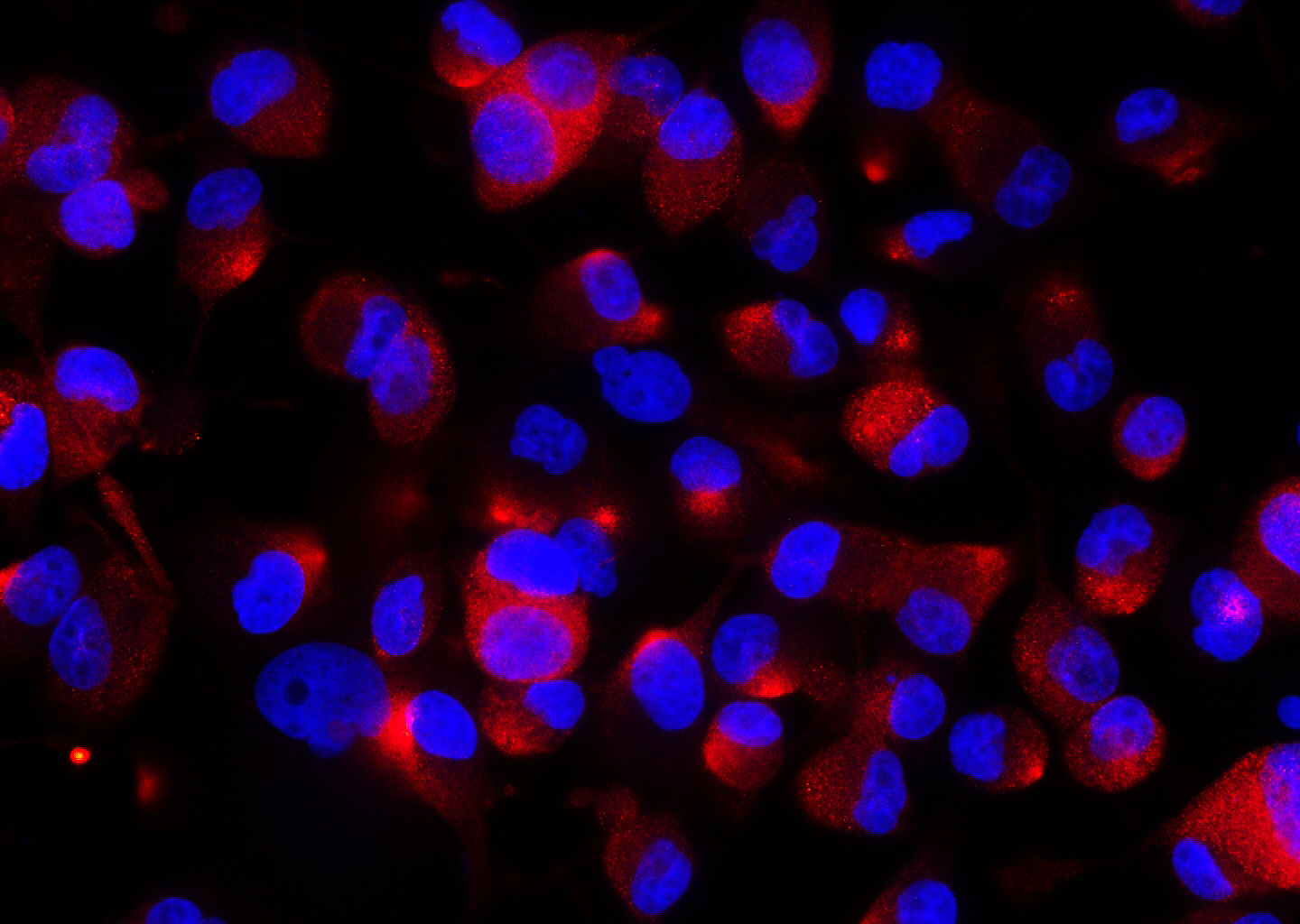

Supplement: Supplementary file 2 [file Data_Sheet_2.ZIP › Immunofluorescence staining/N-cad/A1-1 400-1+2.jpg]

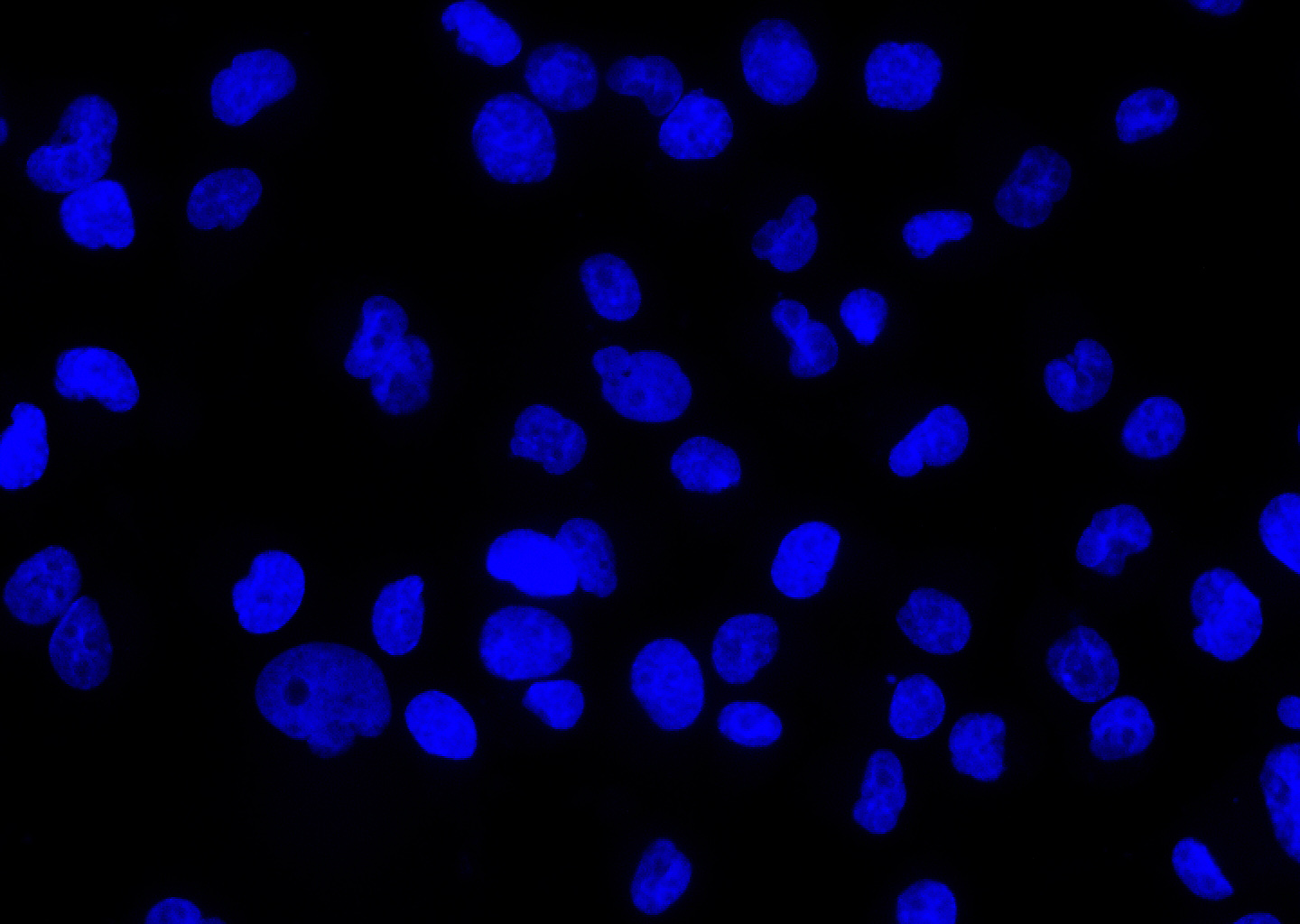

Supplement: Supplementary file 2 [file Data_Sheet_2.ZIP › Immunofluorescence staining/N-cad/A1-1 400-1.jpg]

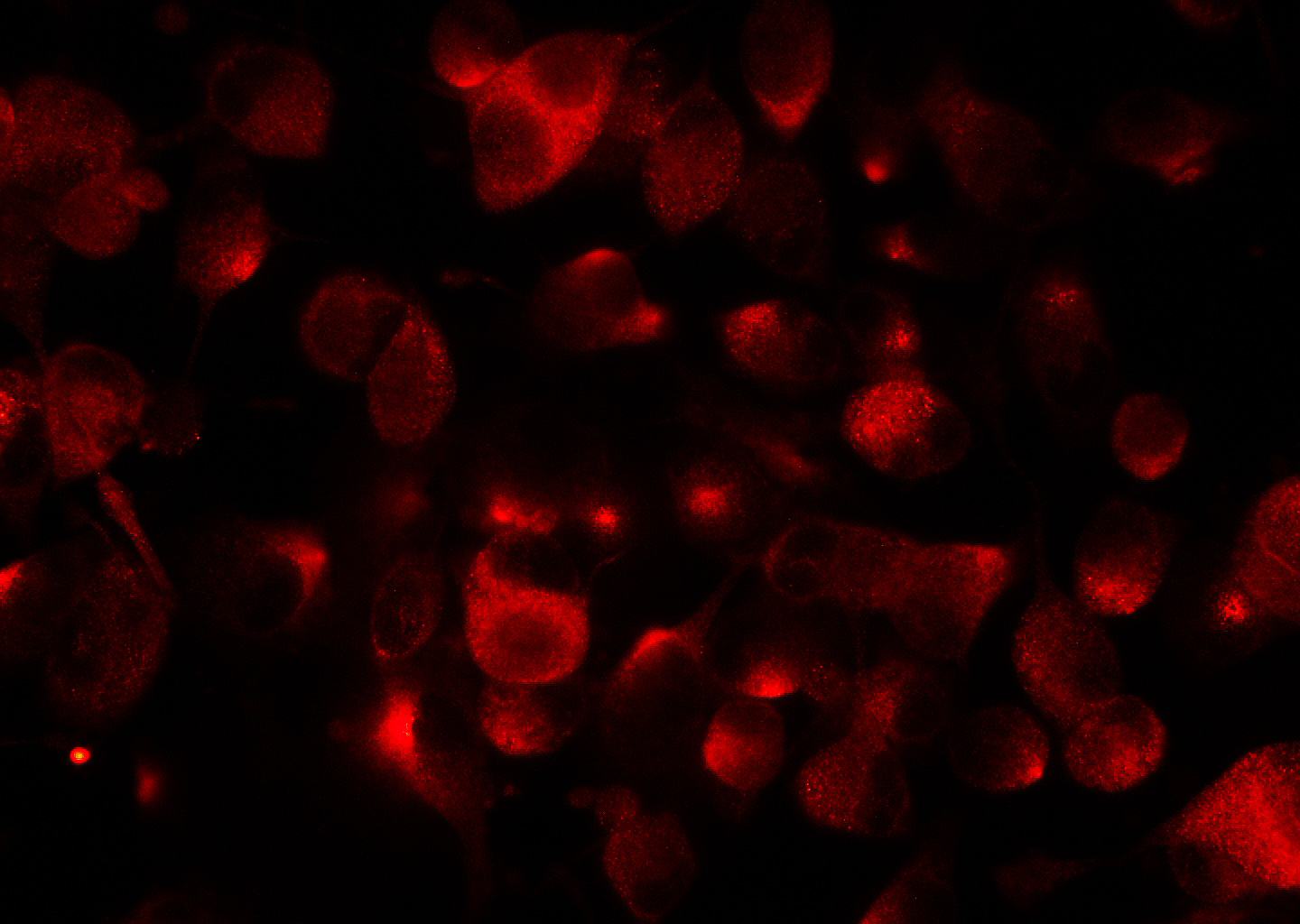

Supplement: Supplementary file 2 [file Data_Sheet_2.ZIP › Immunofluorescence staining/N-cad/A1-1 400-2.jpg]

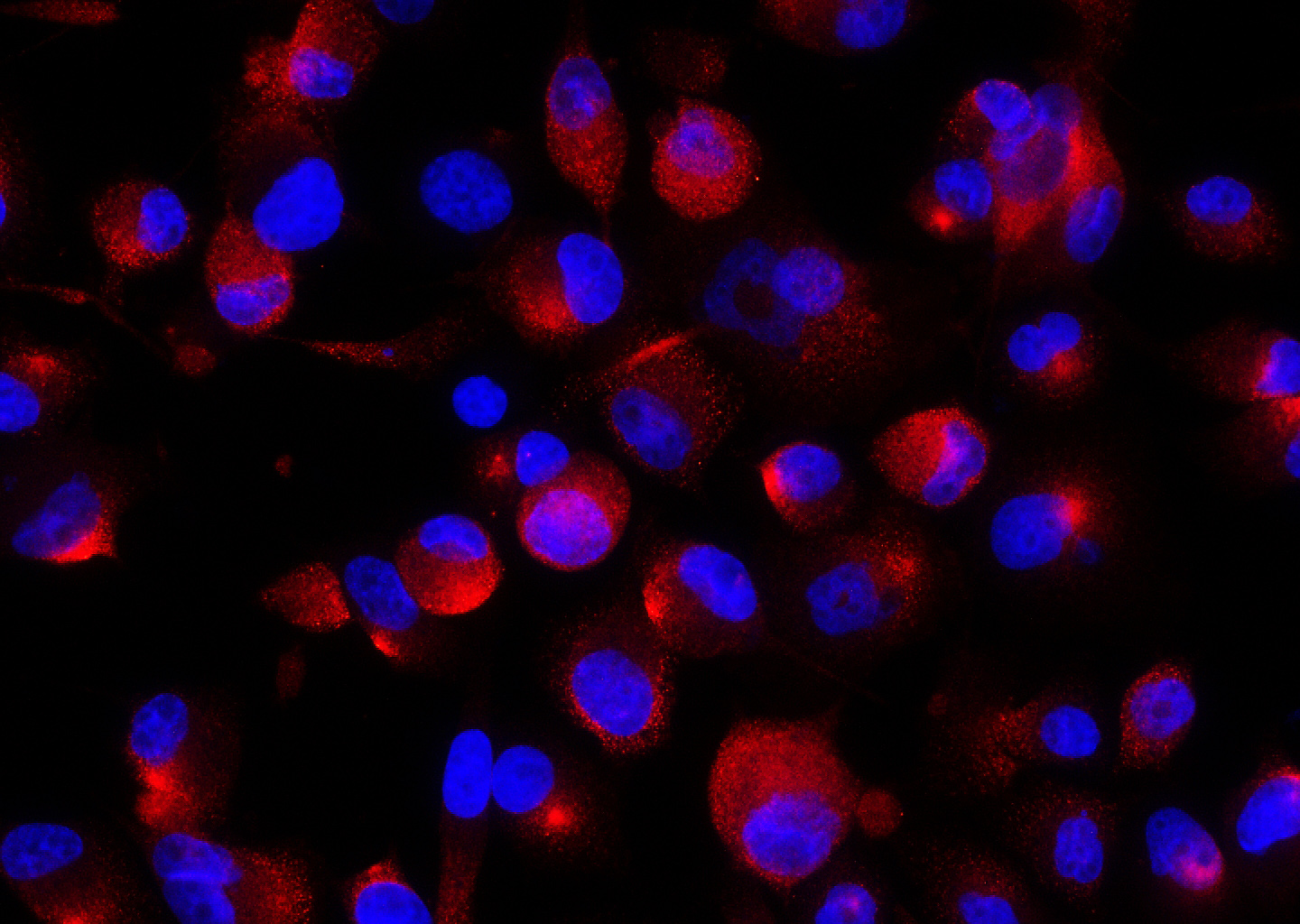

Supplement: Supplementary file 2 [file Data_Sheet_2.ZIP › Immunofluorescence staining/N-cad/B1-1 400-1+2.jpg]

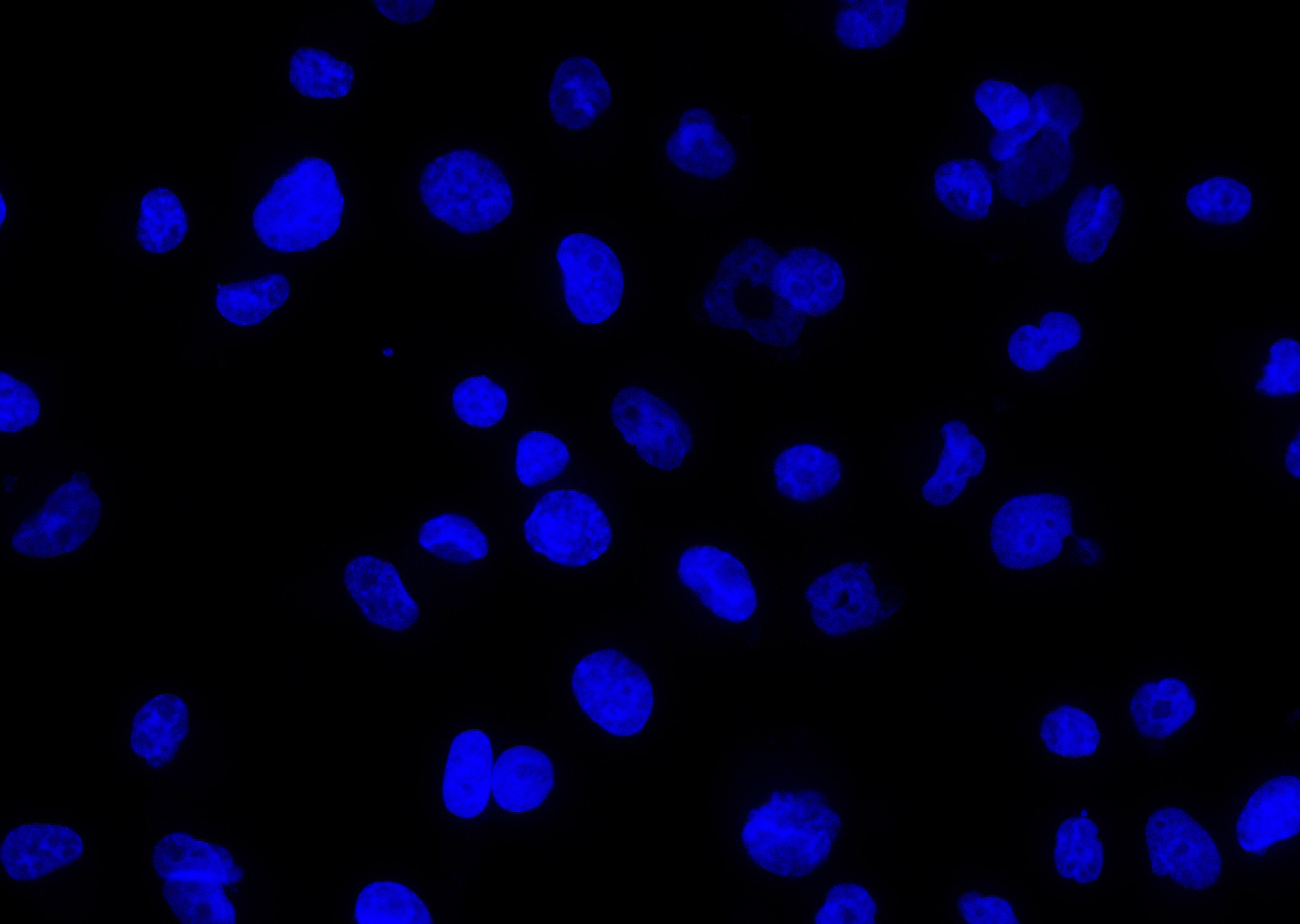

Supplement: Supplementary file 2 [file Data_Sheet_2.ZIP › Immunofluorescence staining/N-cad/B1-1 400-1.jpg]

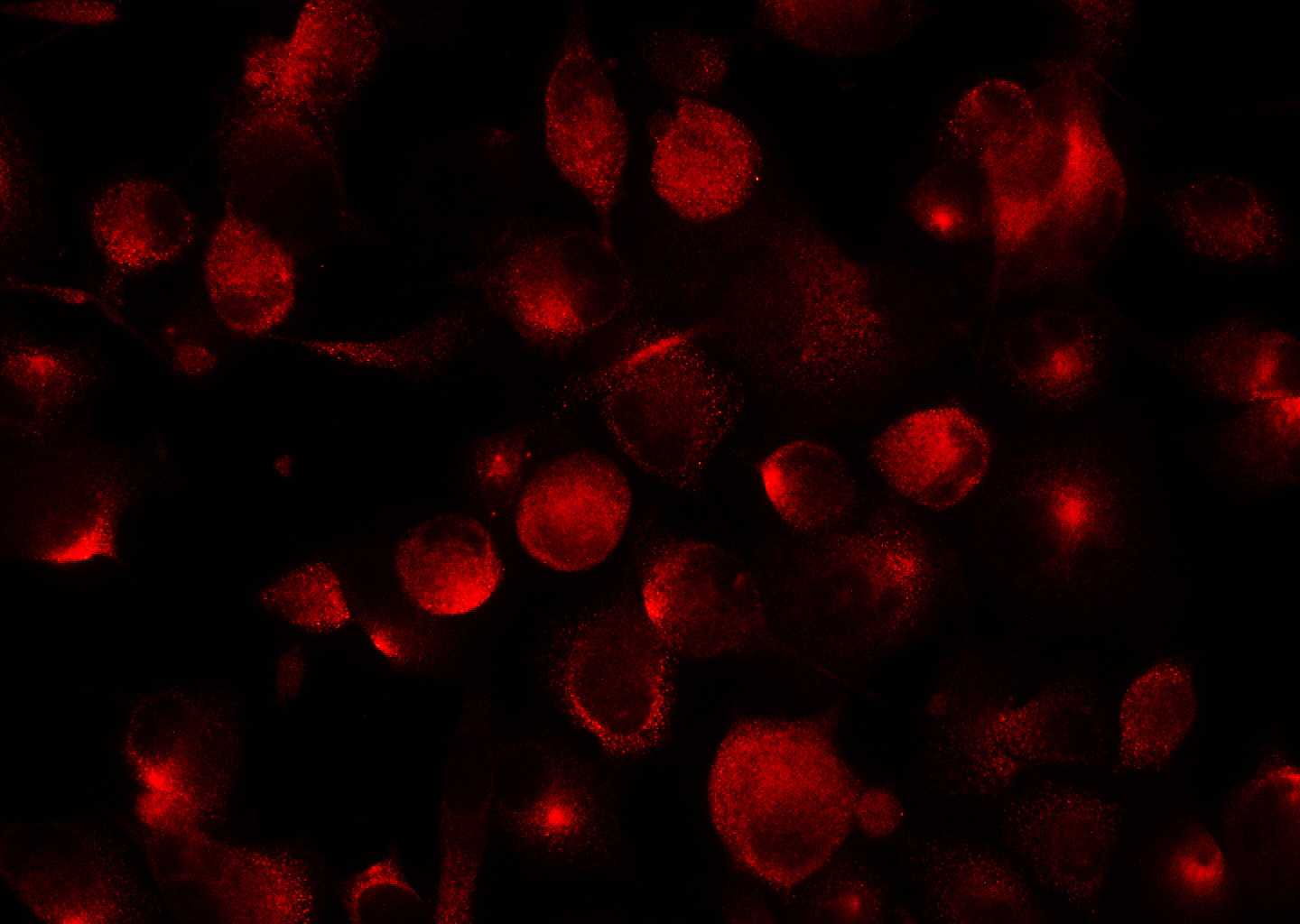

Supplement: Supplementary file 2 [file Data_Sheet_2.ZIP › Immunofluorescence staining/N-cad/B1-1 400-2.jpg]

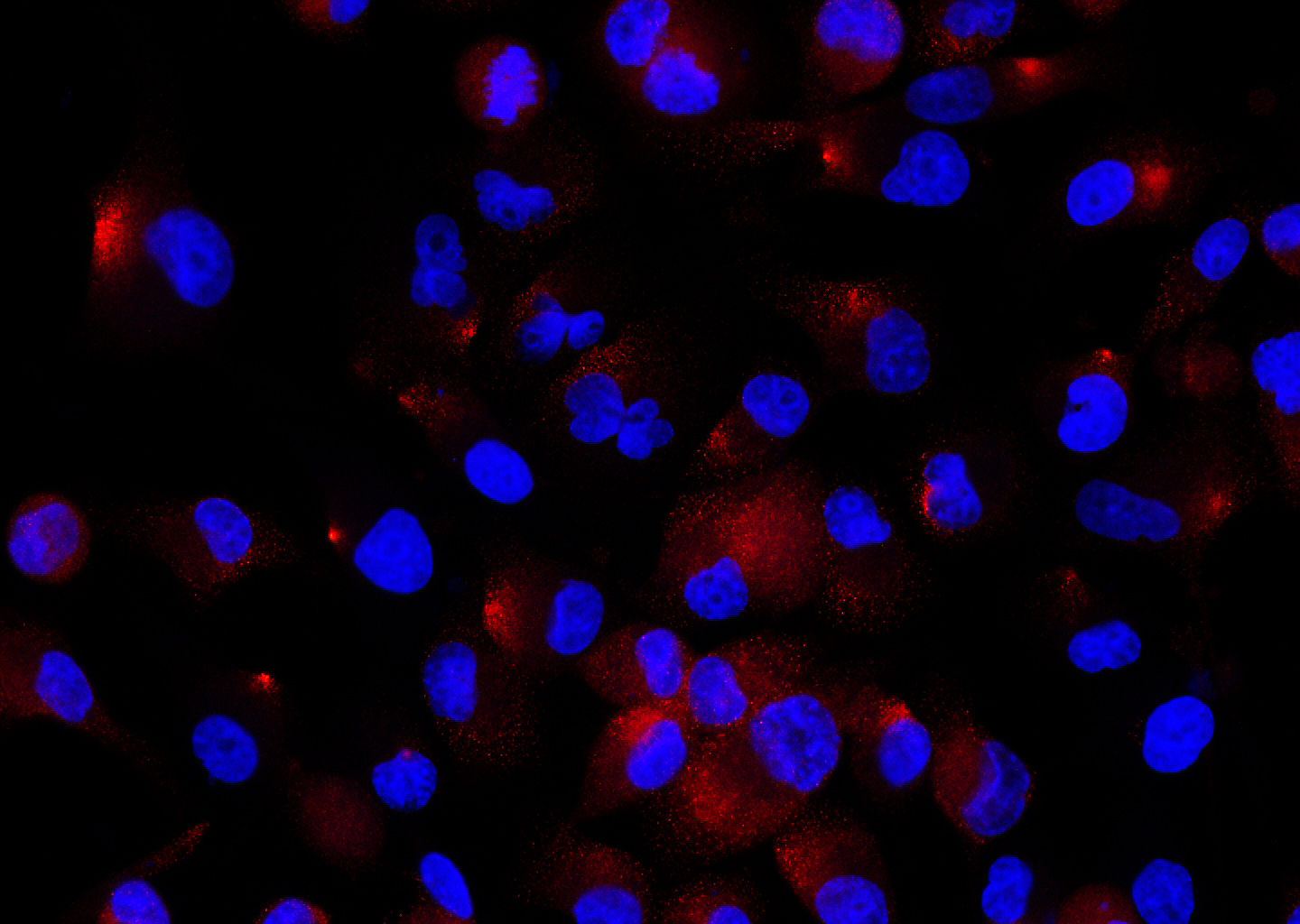

Supplement: Supplementary file 2 [file Data_Sheet_2.ZIP › Immunofluorescence staining/N-cad/C1-1 400-3+4.jpg]

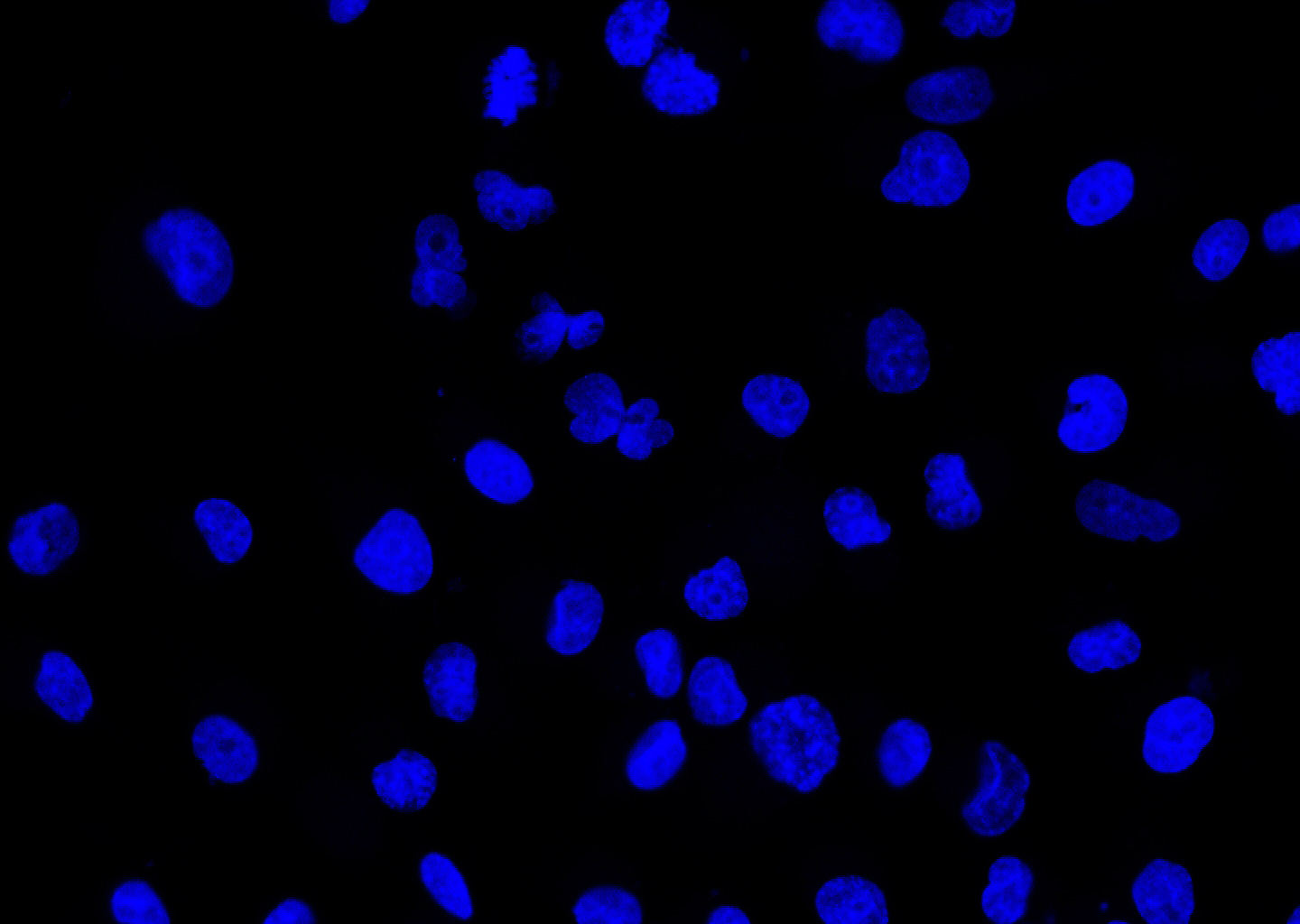

Supplement: Supplementary file 2 [file Data_Sheet_2.ZIP › Immunofluorescence staining/N-cad/C1-1 400-3.jpg]

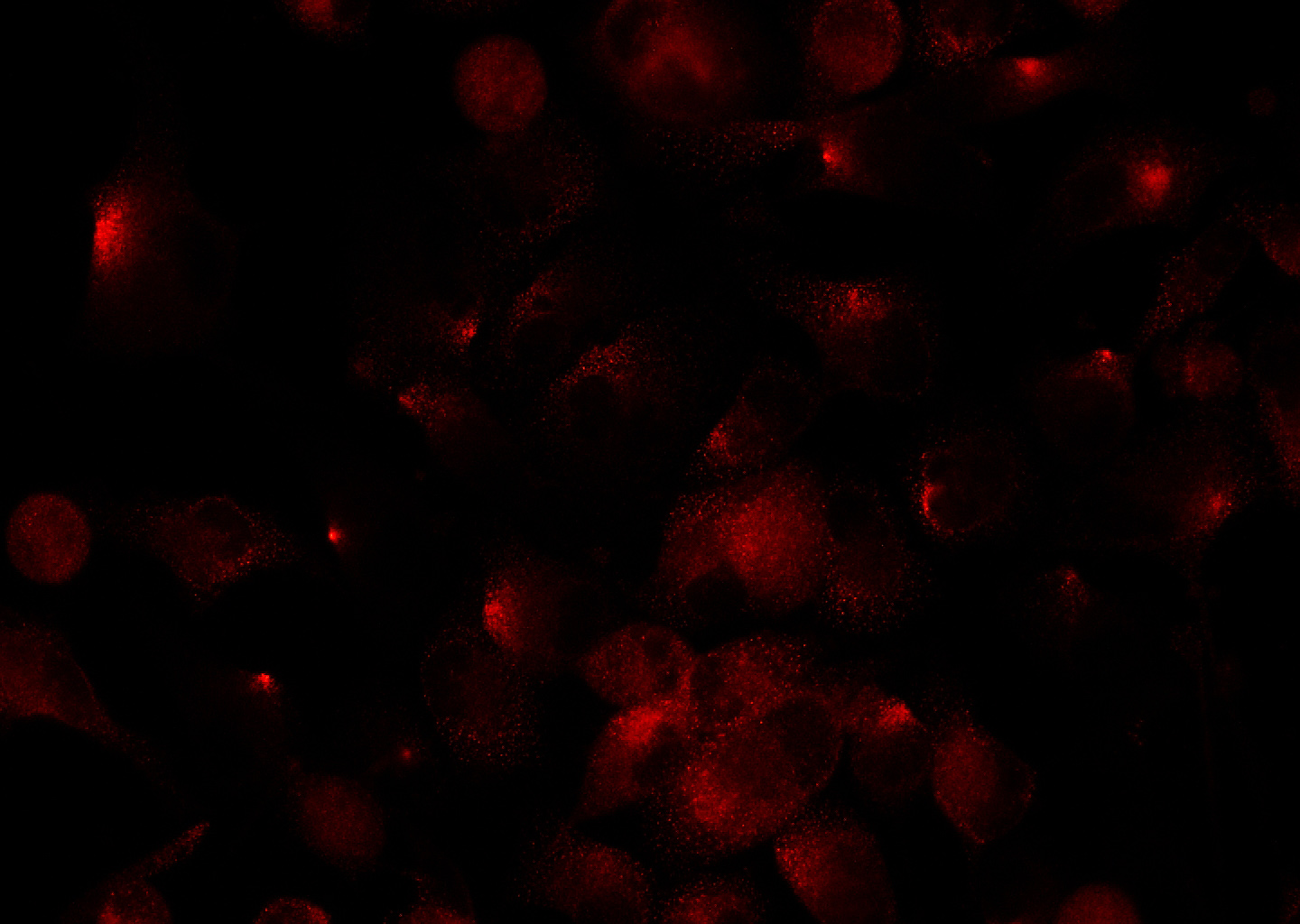

Supplement: Supplementary file 2 [file Data_Sheet_2.ZIP › Immunofluorescence staining/N-cad/C1-1 400-4.jpg]

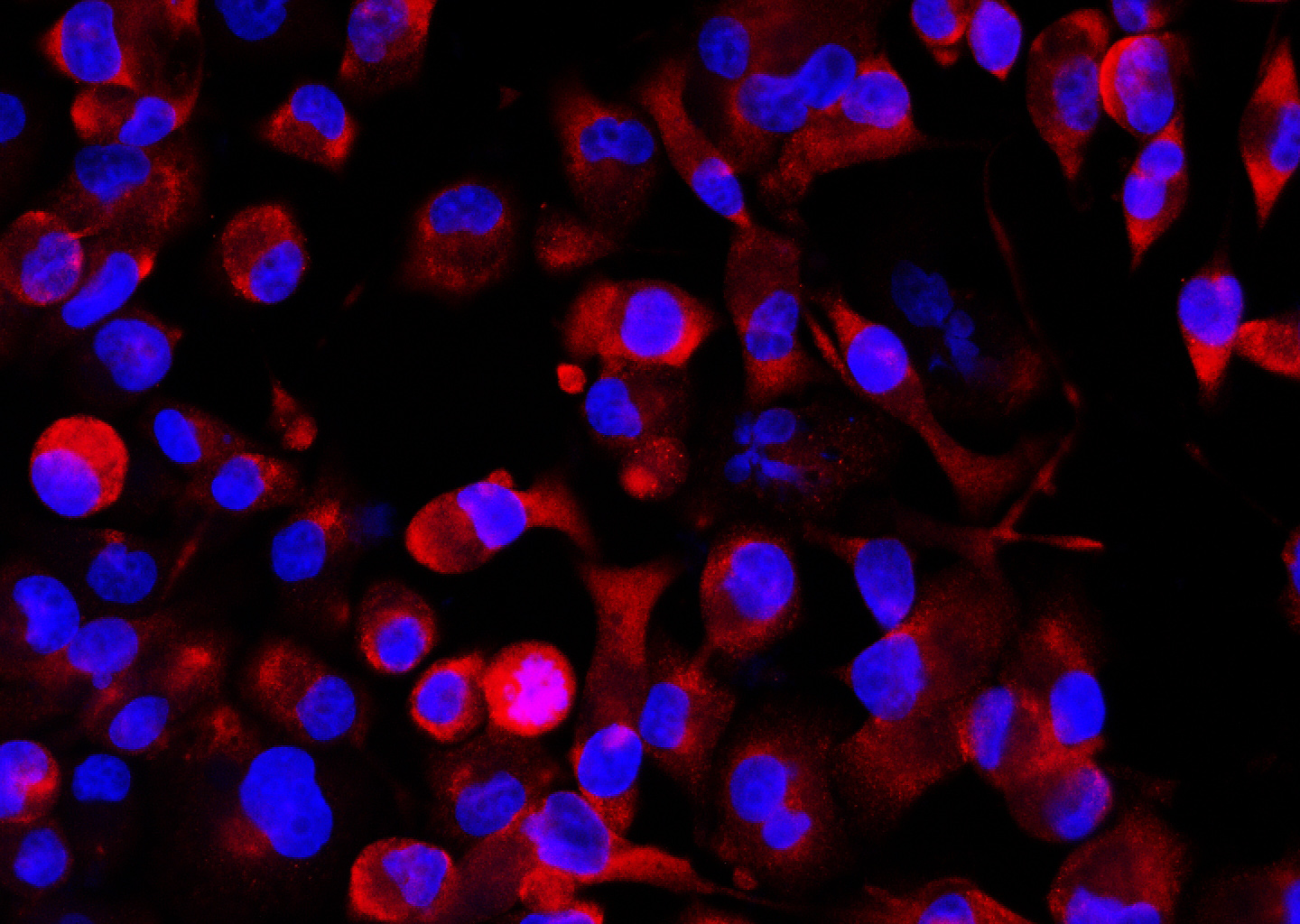

Supplement: Supplementary file 2 [file Data_Sheet_2.ZIP › Immunofluorescence staining/beta-cat/A1-3 400-3+4.jpg]

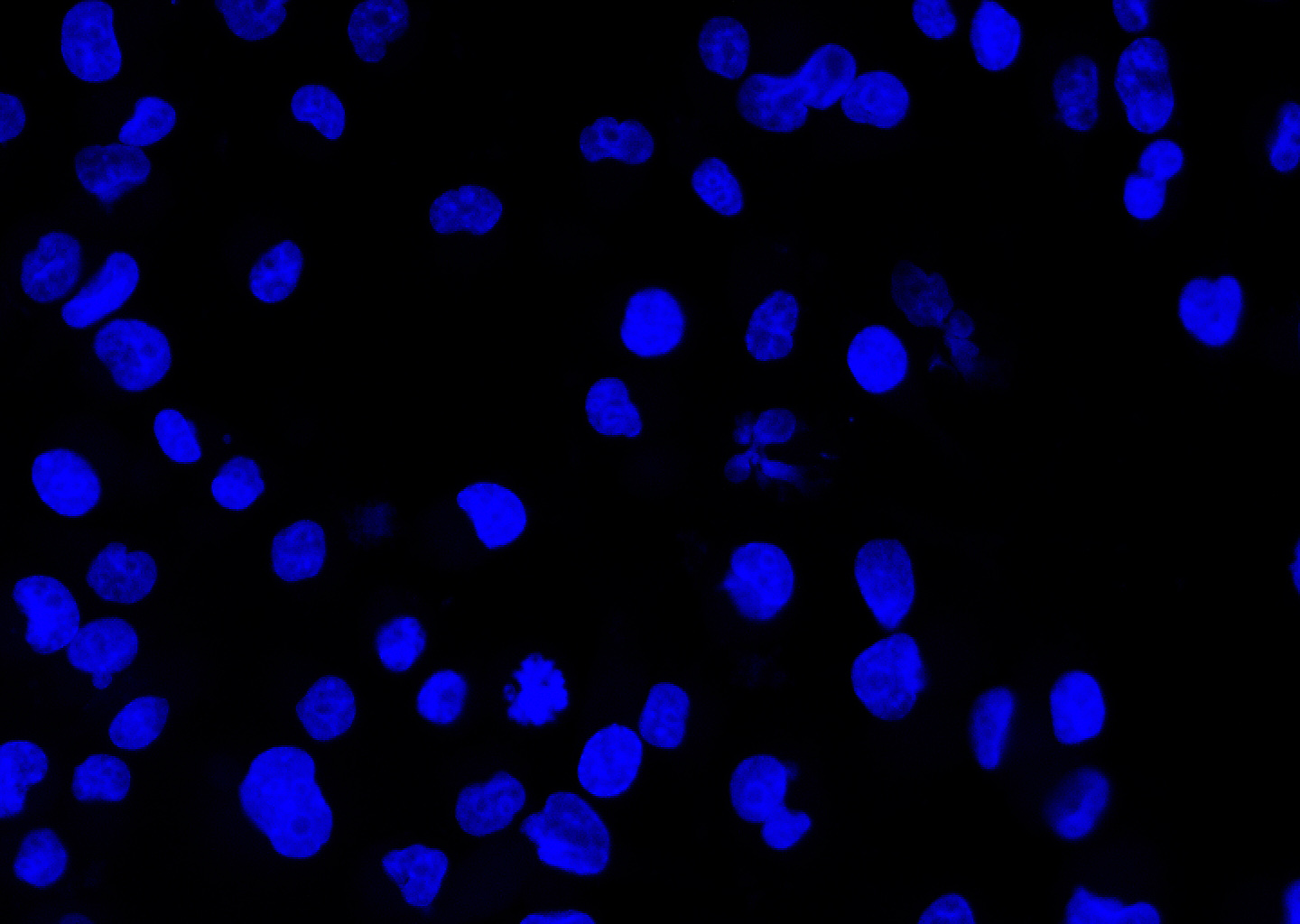

Supplement: Supplementary file 2 [file Data_Sheet_2.ZIP › Immunofluorescence staining/beta-cat/A1-3 400-3.jpg]

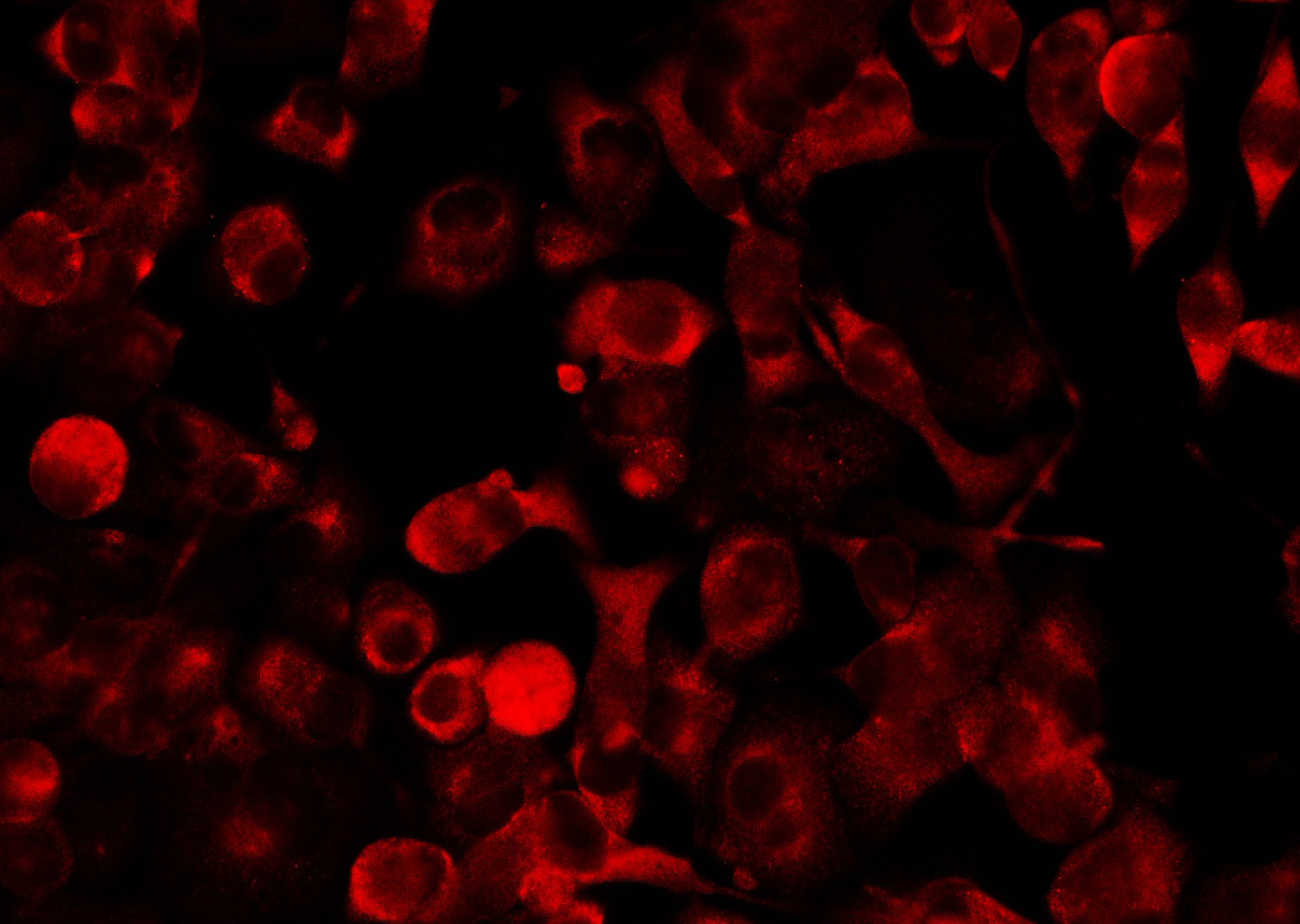

Supplement: Supplementary file 2 [file Data_Sheet_2.ZIP › Immunofluorescence staining/beta-cat/A1-3 400-4.jpg]

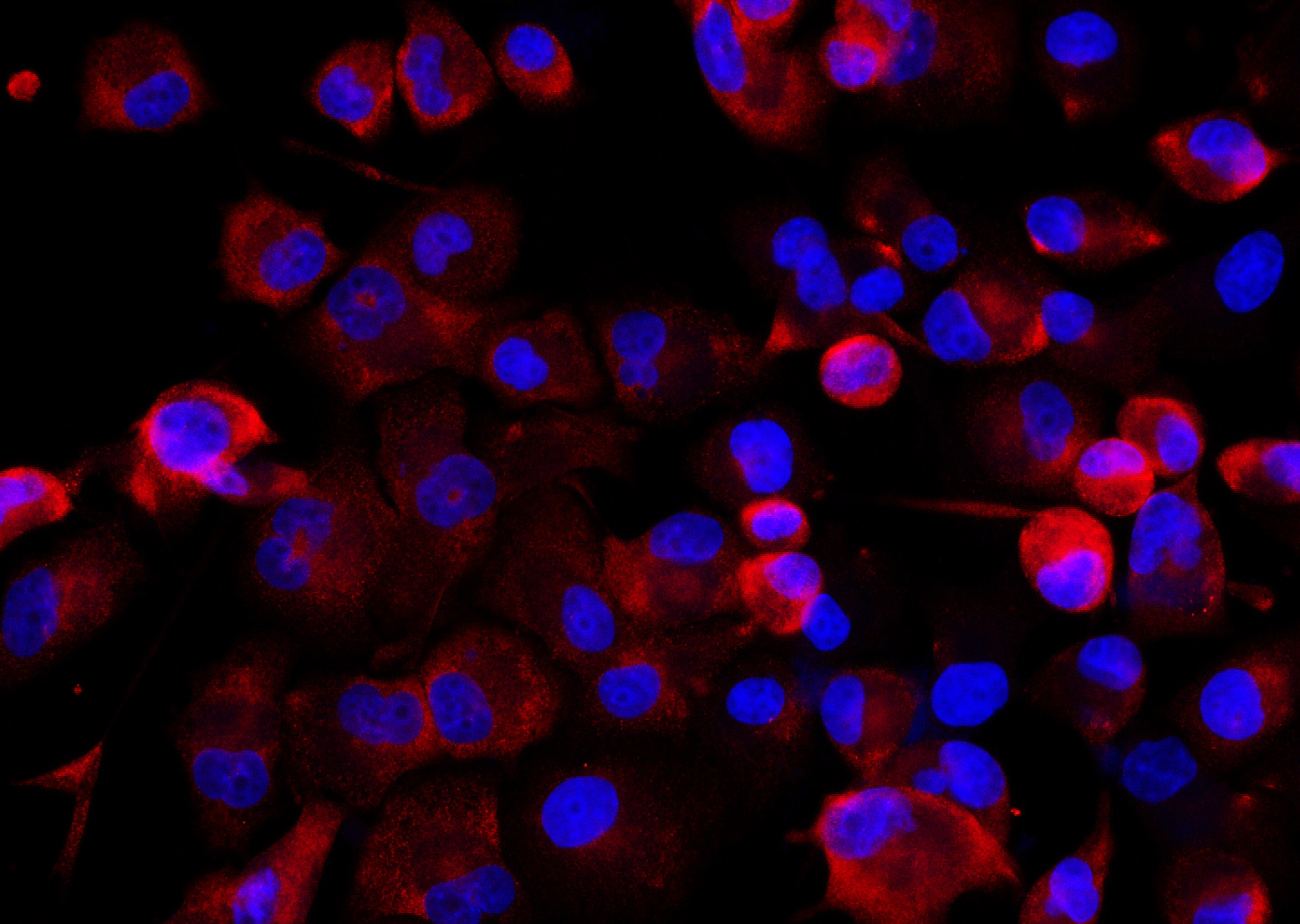

Supplement: Supplementary file 2 [file Data_Sheet_2.ZIP › Immunofluorescence staining/beta-cat/B1-3 400-3+4.jpg]

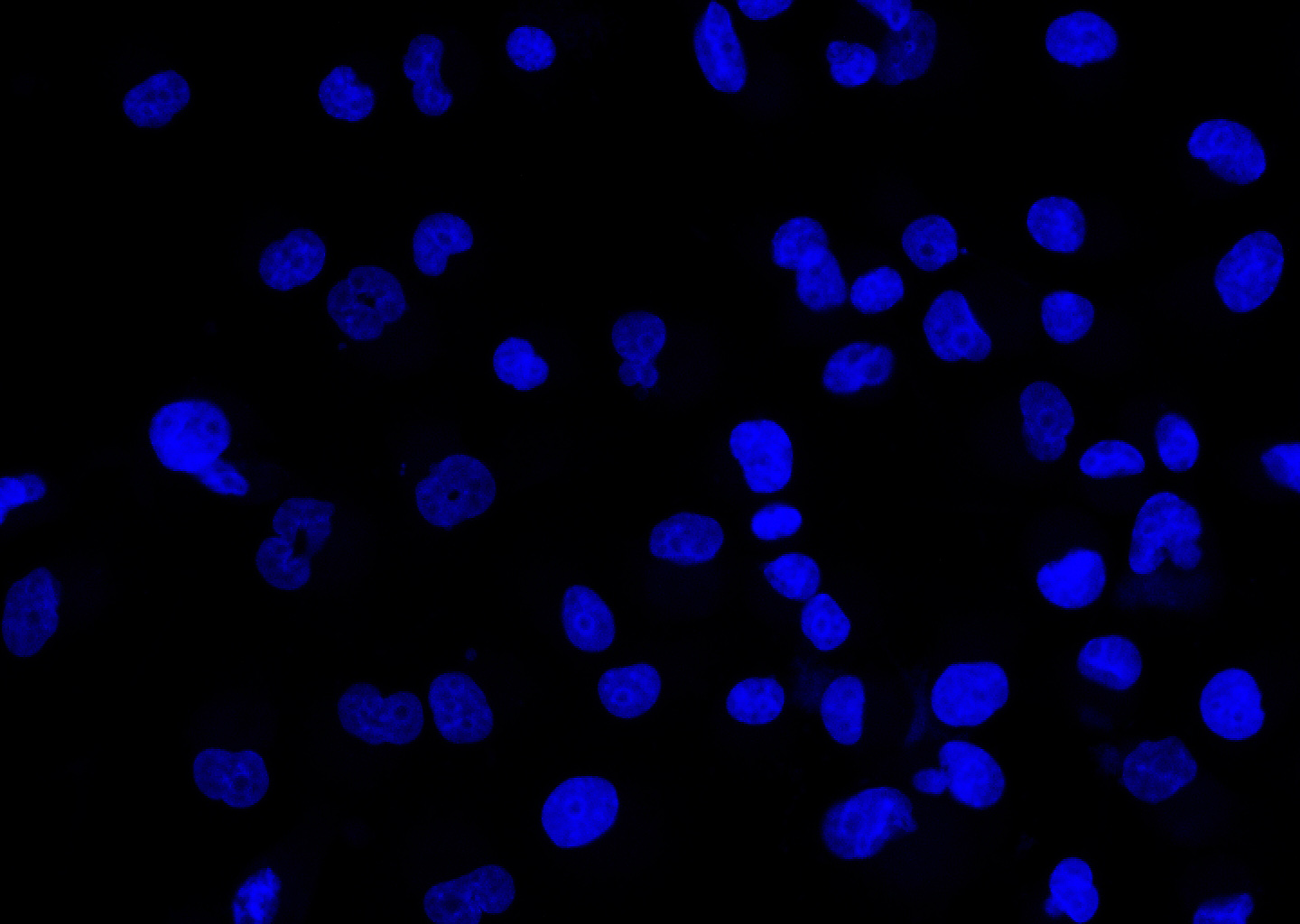

Supplement: Supplementary file 2 [file Data_Sheet_2.ZIP › Immunofluorescence staining/beta-cat/B1-3 400-3.jpg]

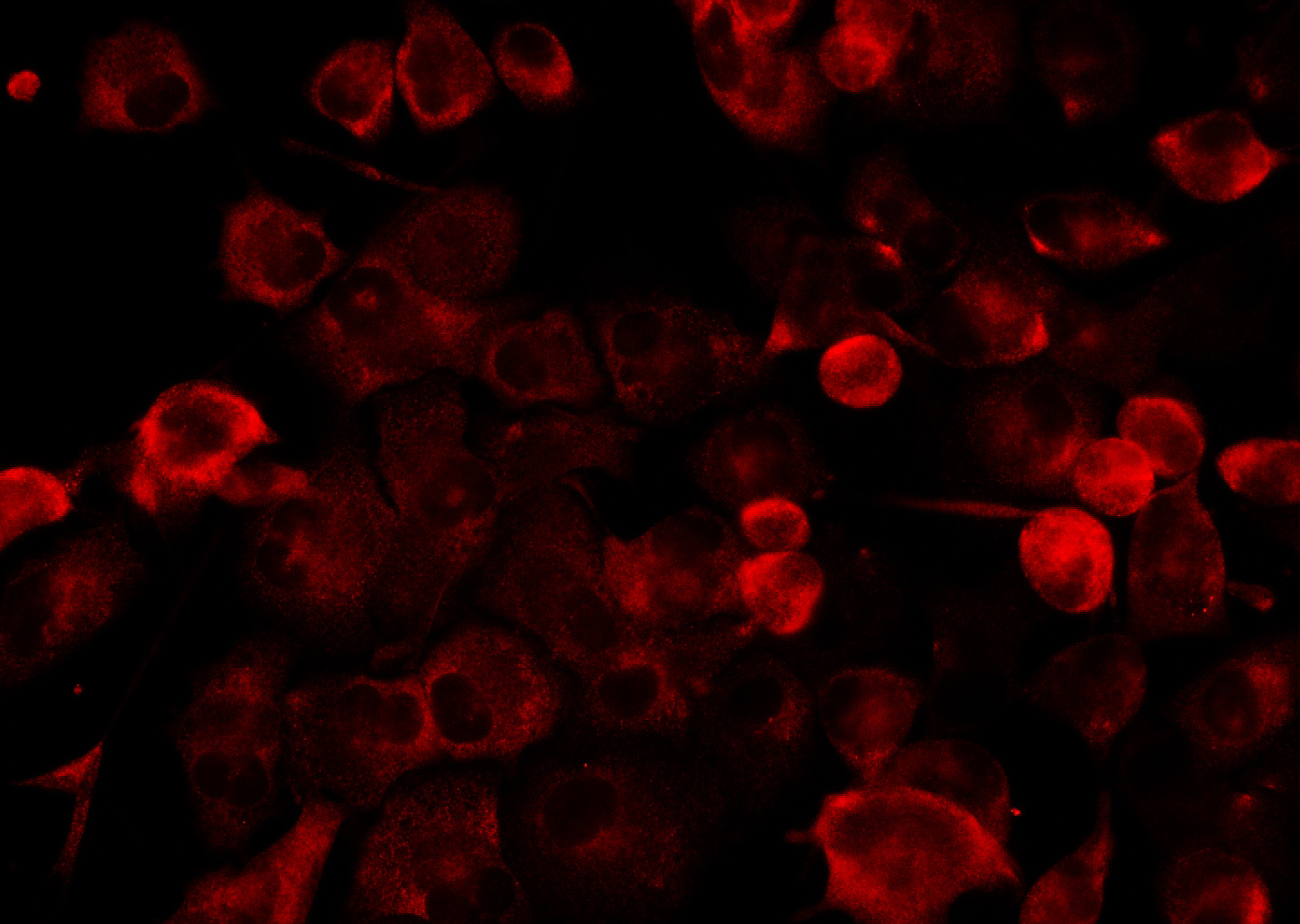

Supplement: Supplementary file 2 [file Data_Sheet_2.ZIP › Immunofluorescence staining/beta-cat/B1-3 400-4.jpg]

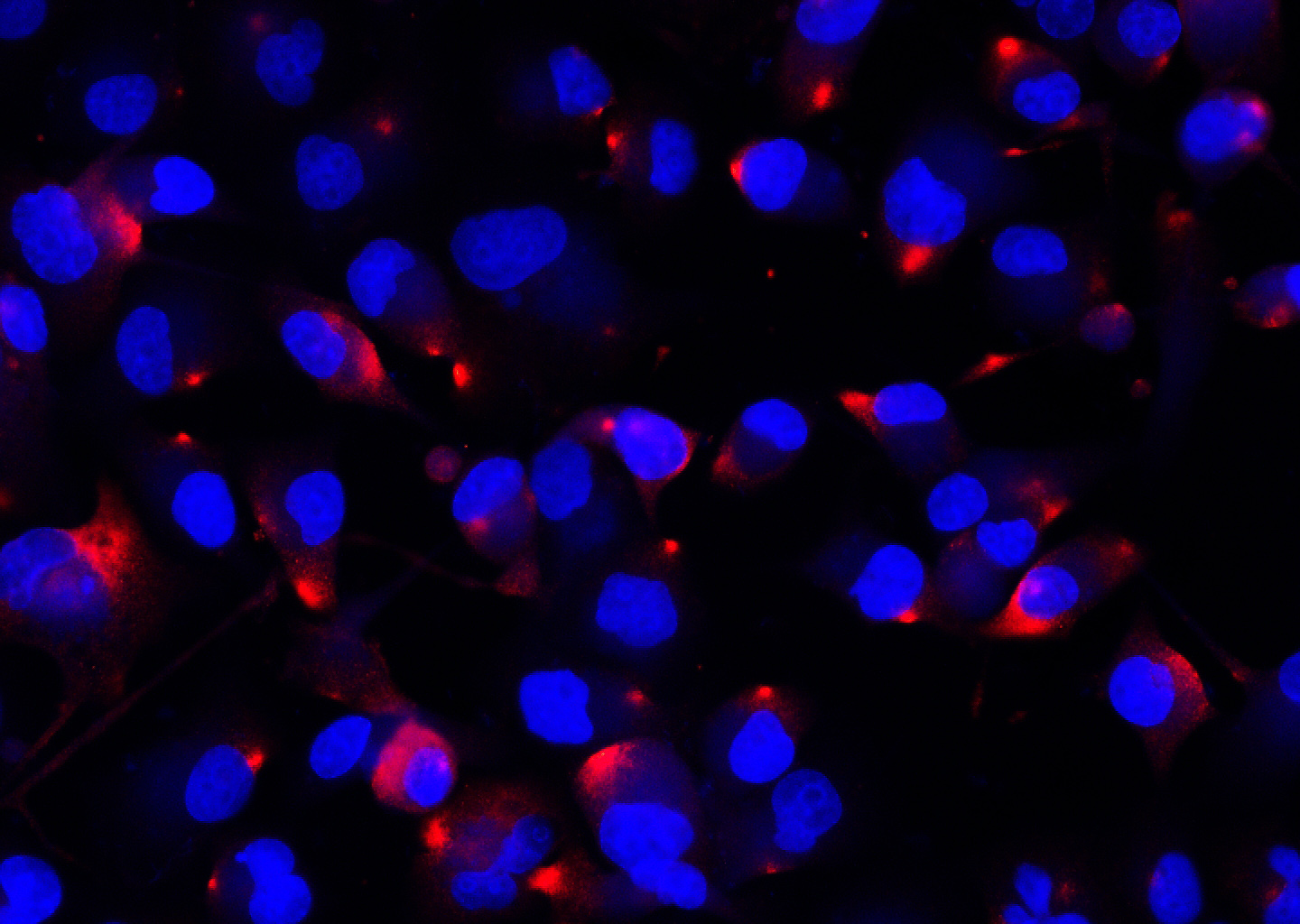

Supplement: Supplementary file 2 [file Data_Sheet_2.ZIP › Immunofluorescence staining/beta-cat/C1-3 400-3+4.jpg]

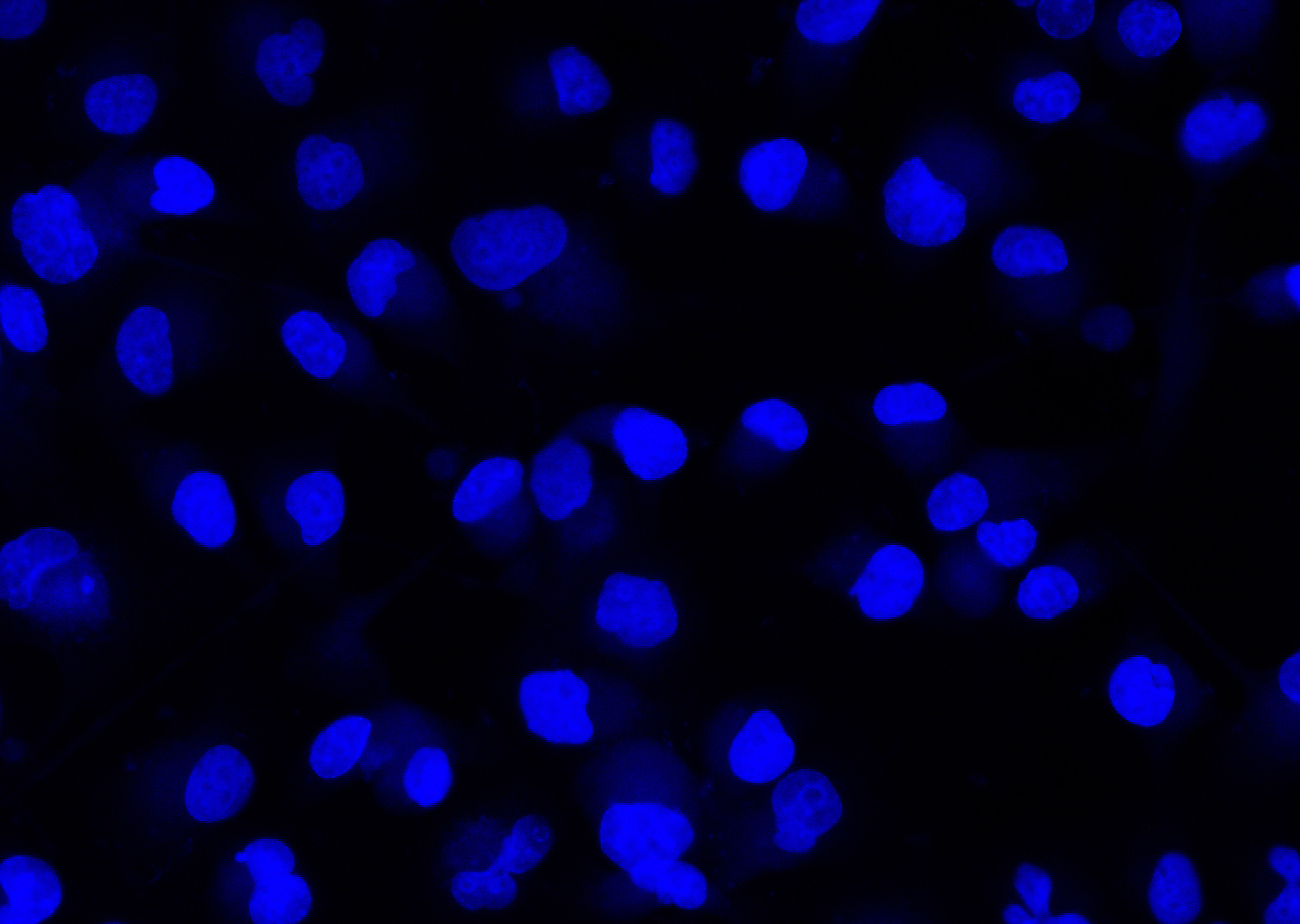

Supplement: Supplementary file 2 [file Data_Sheet_2.ZIP › Immunofluorescence staining/beta-cat/C1-3 400-3.jpg]

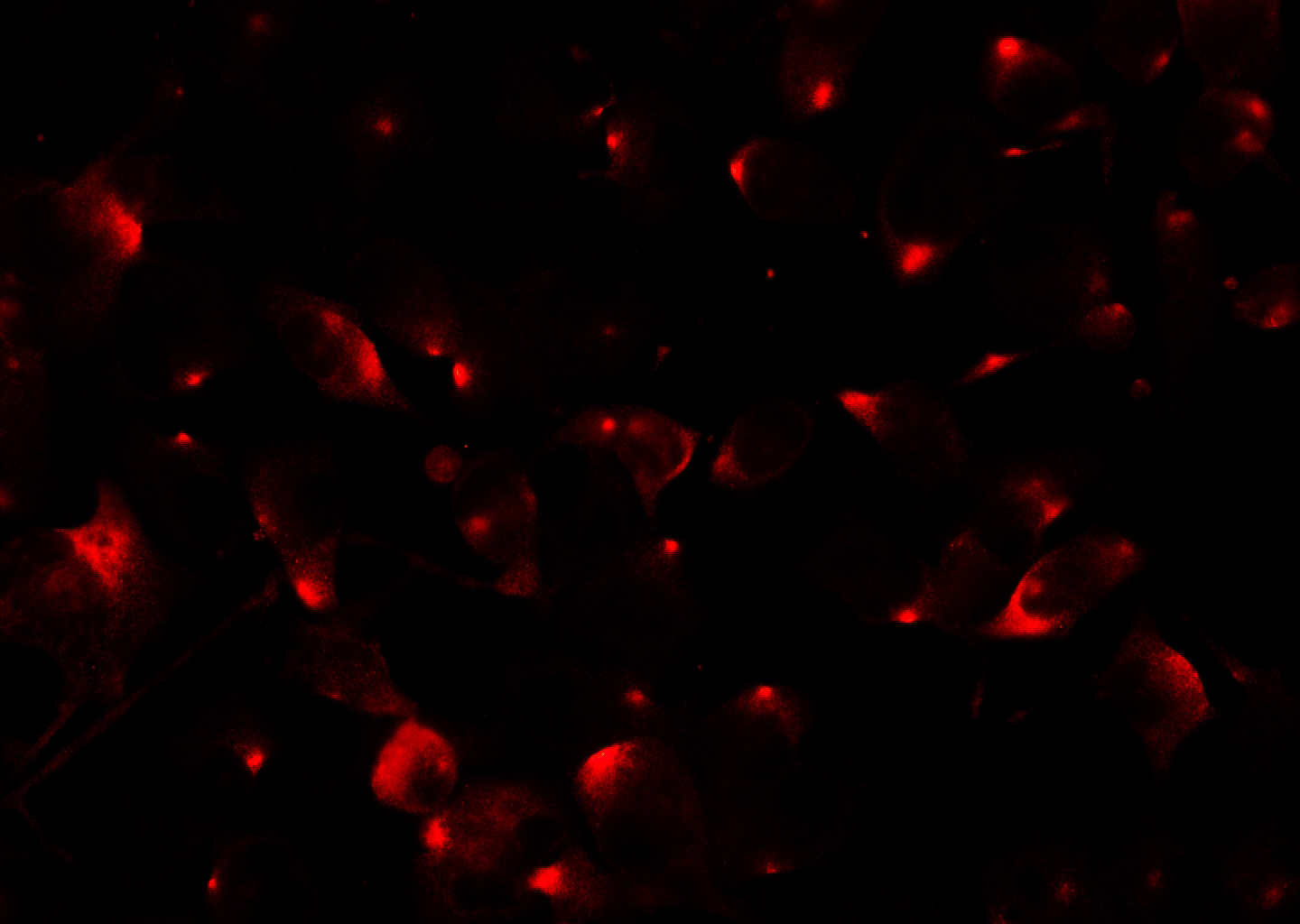

Supplement: Supplementary file 2 [file Data_Sheet_2.ZIP › Immunofluorescence staining/beta-cat/C1-3 400-4.jpg]

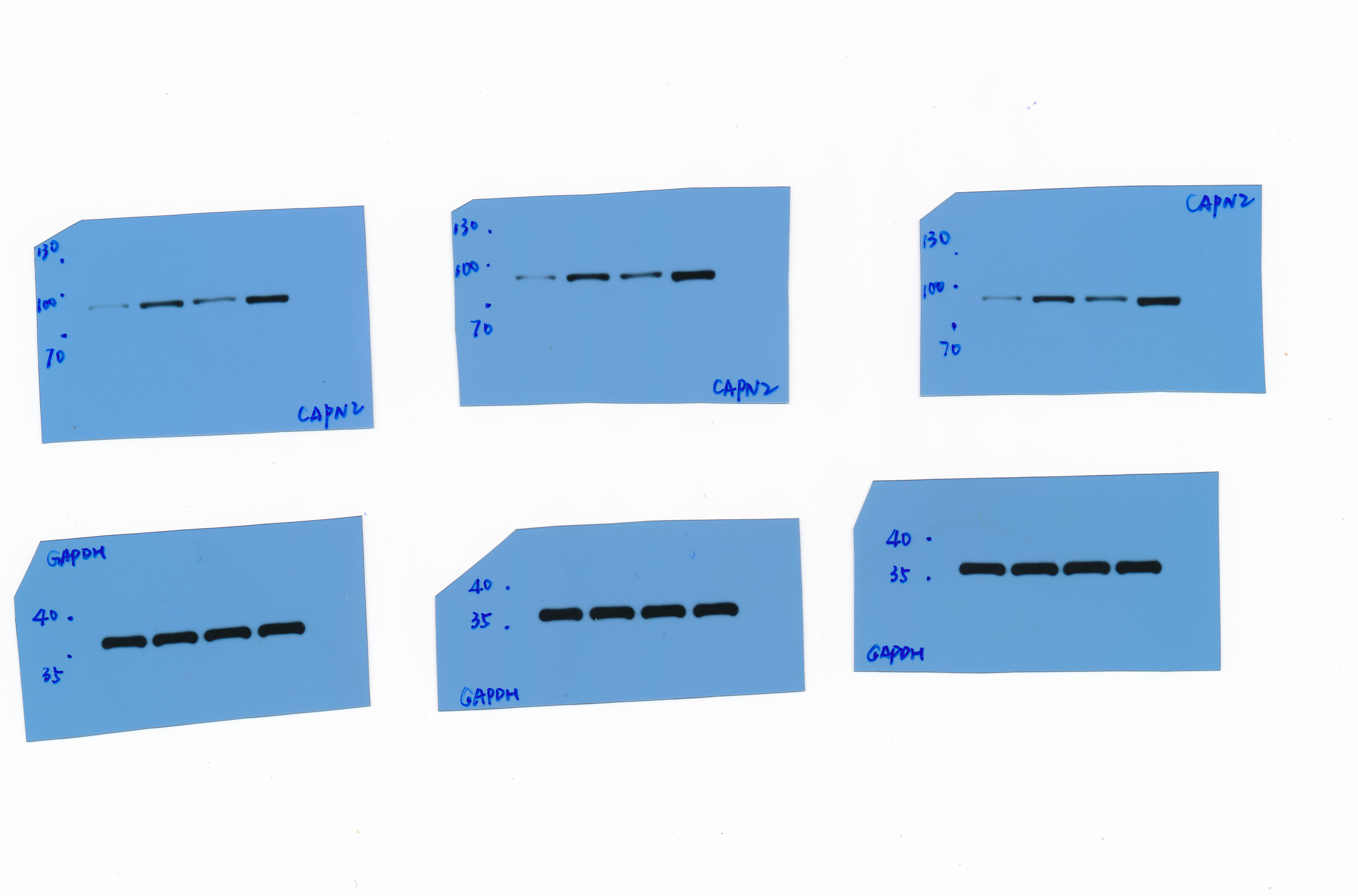

Supplement: Supplementary file 3 [file Data_Sheet_3.ZIP › Western blot1/WB_CAPN2 ╡░░╫▒φ┤∩.tif]

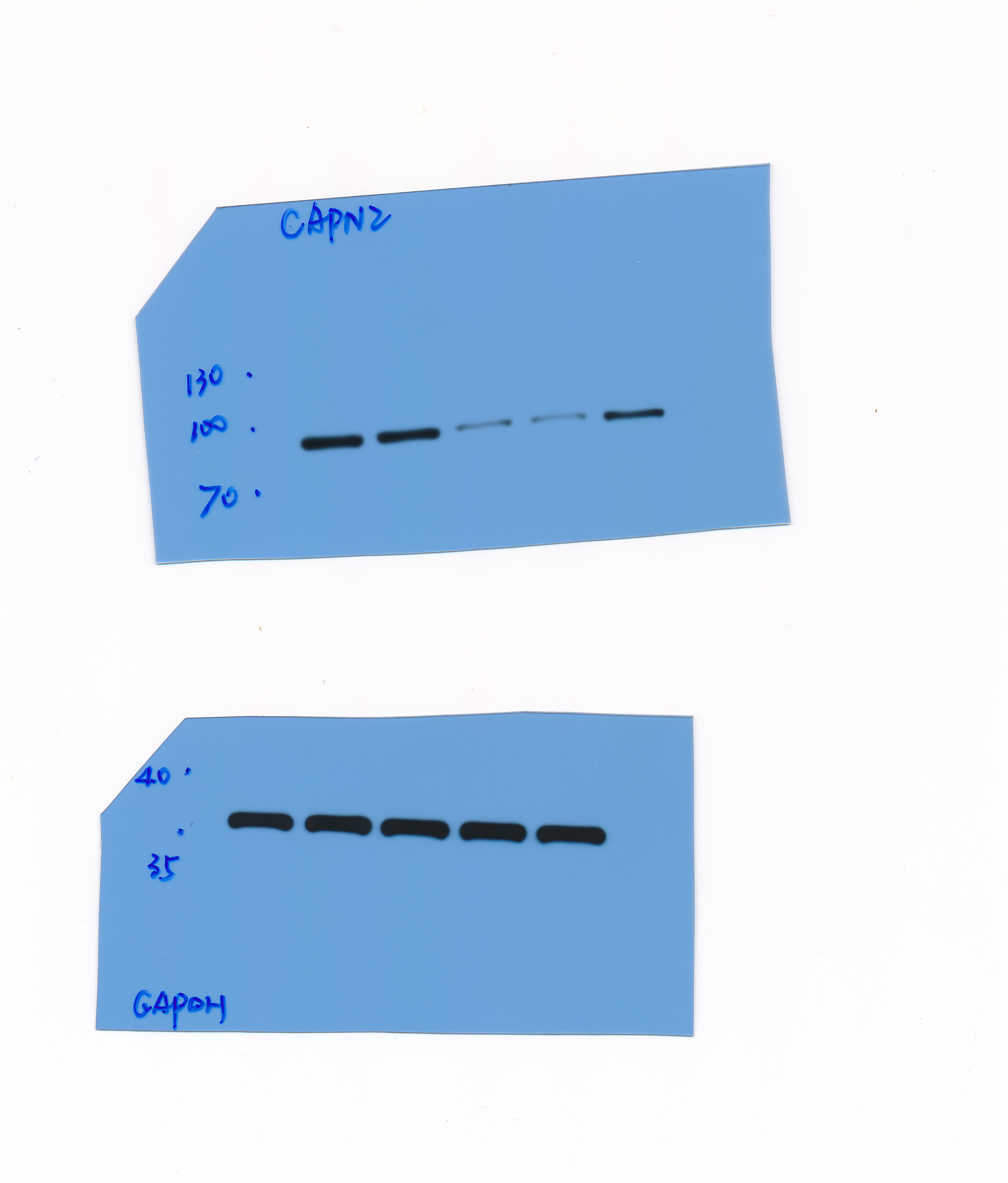

Supplement: Supplementary file 3 [file Data_Sheet_3.ZIP › Western blot1/WB_CAPN2-╫¬╚╛╨o╣√.tif]

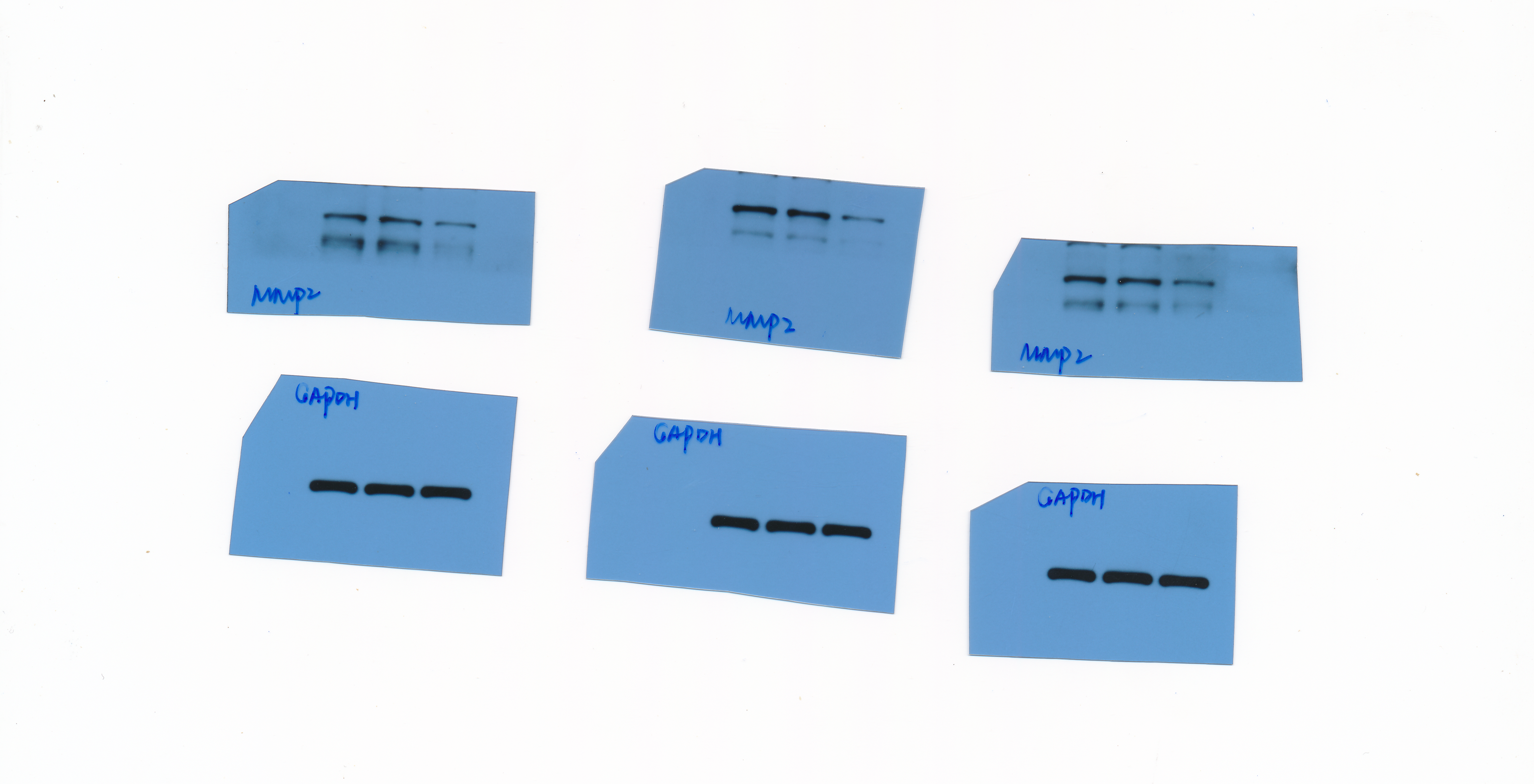

Supplement: Supplementary file 4 [file Data_Sheet_4.ZIP › Western blot2/WB-MMP2.tif]

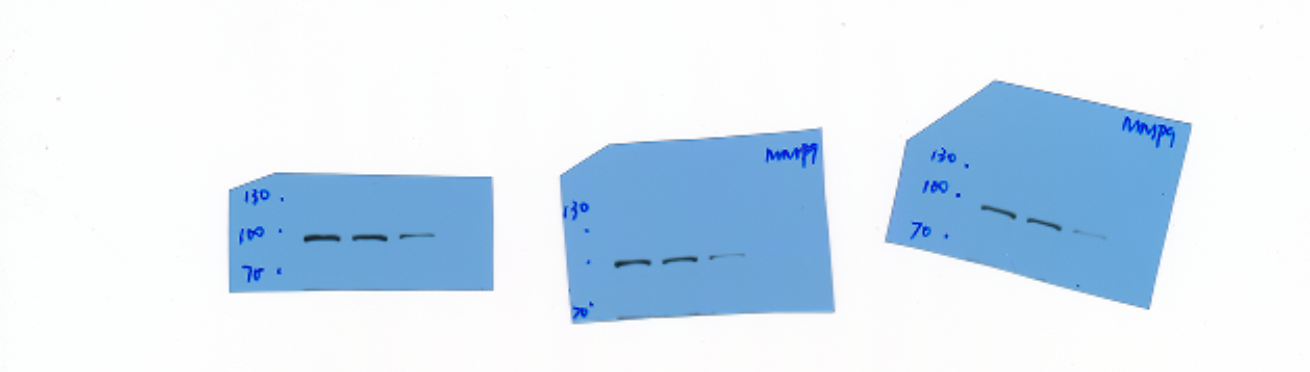

Supplement: Supplementary file 4 [file Data_Sheet_4.ZIP › Western blot2/WB-MMP9.tif]

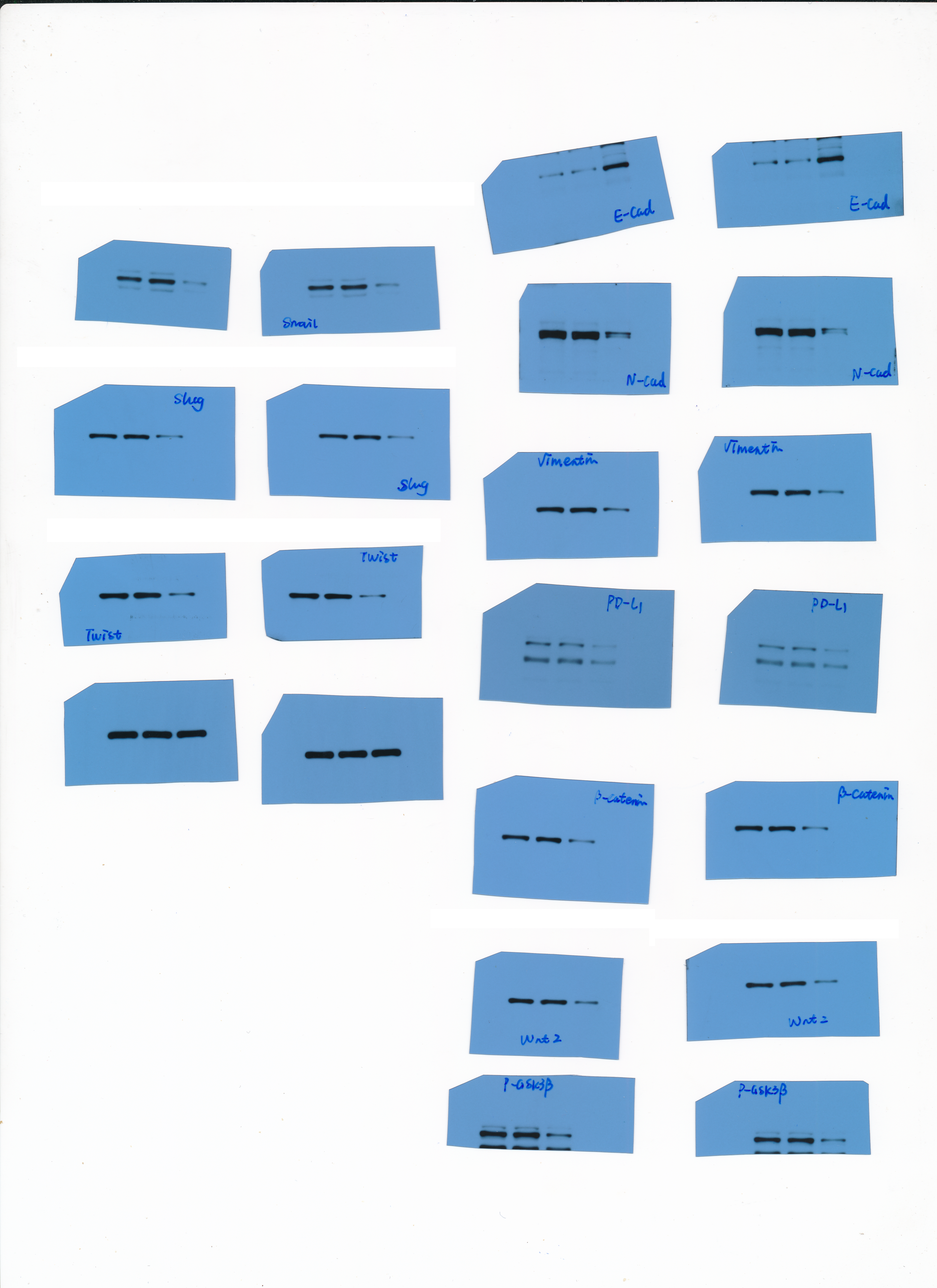

Supplement: Supplementary file 4 [file Data_Sheet_4.ZIP › Western blot2/WB-Wnt.tif]
